# Photoradiolabeling of onartuzumab with ^99m^Tc and ^188^Re-tricarbonyl for radiotheranostics of gastric cancer†

**DOI:** 10.1039/d4sc08089k

**Published:** 2025-03-13

**Authors:** Jonas Genz, Cesare Berton, Samy Kichou, Simon Klingler, Mirja C. Nolff, Henrik Braband, Jason P. Holland

**Affiliations:** a University of Zurich, Department of Chemistry Winterthurerstrasse 190 CH-8057 Zurich Switzerland jason.holland@chem.uzh.ch +41 44 63 53 990 www.hollandlab.org; b Klinik für Kleintierchirurgie, Vetsuisse-Fakultät, University of Zurich Winterthurerstrasse 260 CH-8057 Zurich Switzerland

## Abstract

The clinically relevant nuclear isomer of technetium-99 (^99m^Tc) and the radionuclides rhenium-186/188 (^186^Re and ^188^Re) represent an almost ideal match for the development of radiotracers for applications in diagnostic imaging and molecularly targeted radionuclide therapy. Although the chemistry of Tc and Re is similar, important differences arise in both the synthesis and properties of their complexes. Here, we report the synthesis and characterization of ^99m^Tc- and ^188^Re-onartuzumab by labeling of the cancer-specific mAb onartuzumab (MetMAb) with the corresponding metal-tricarbonyl complexes derived from a novel photoactivatable ligand. The acyclic *tris*-amine ligand L1, featuring a photoactive aryl azide (ArN_3_) group, was synthesized from *N*^1^-(2-aminoethyl)ethane-1,2-diamine in 5 steps with an overall yield of 32%. Radiosynthesis of the [M(CO)_3_L1]^+^ (M = ^99m^Tc or ^188^Re) photoactivatable complexes was accomplished *via* reduction of the [M^VII^O_4_]^−^ species to give the intermediate ^99m^Tc^I^- and ^188^Re^I^-tricarbonyl-triaquo followed by ligand substitution with L1. The light-induced photoradiosynthesis of [M(CO)_3_L1-azepin]-onartuzumab (M-onartuzumab; M = ^99m^Tc or ^188^Re) was achieved by irradiating the [M(CO)_3_L1]^+^ complexes in the presence of onartuzumab (formulated as MetMAb), with 395 nm light for 15 minutes at room temperature. Photoradiolabeling reactions produced M-onartuzumab radioimmunoconjugates in decay-corrected radiochemical yields of 20–30%, high radiochemical purities (RCP > 95%), and in molar activities of 1.026–4.146 MBq nmol^−1^. Cellular binding assays confirmed the specificity of radiotracer binding toward human hepatocyte growth-factor receptor (c-MET) expression on the surface of MNK-45 gastric adenocarcinoma cells. Subsequent planar γ-ray scintigraphy imaging and *ex vivo* biodistribution experiments in mouse models bearing subcutaneous MKN-45 xenografts revealed specific tumor targeting compared against competitive inhibition (blocking) controls performed at 24 hours (^99m^Tc and ^188^Re) and 72 hours (^188^Re). Tumor uptake reached 20.20 ± 4.05 %ID g^−1^ for ^99m^Tc-onartuzumab and 22.13 ± 3.11 %ID g^−1^ for ^188^Re-onartuzumab after 24 hours. Blocking experiments confirmed tumor specificity, with a reduction in tumor uptake of approximately 70% for both ^99m^Tc-onartuzumab and ^188^Re-onartuzumab. Experimental data also revealed the biochemical equivalence of ^99m^Tc-onartuzumab and ^188^Re-onartuzumab in terms of stability and pharmacokinetics *in vivo*. For ^188^Re-onartuzumab, activity was retained in the tumor for over 72 hours, with uptake levels at 20.21 ± 1.47 %ID g^−1^. Overall, the experiments demonstrated that photoradiosynthesis can be employed to develop a variety of rhenium based radioimmunoconjugates for future applications in tumor targeted radioimmunotherapy. Furthermore, these results underline the high potential of rhenium and technetium radioconjugates as theranostic platforms.

## Abstract 2

Light-induced bioconjugation and radiolabeling of the cancer-specific monoclonal antibody onartuzumab was accomplished using the tricarbonyl chemistry of the radiotheranostic matched pair of nuclides ^99m^Tc and ^188^Re.

## Graphical abstract

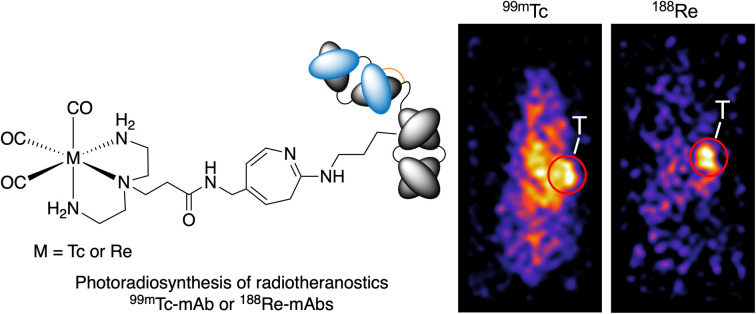

## Introduction

Technetium-99m (^99m^Tc) and rhenium-188 (^188^Re) are radionuclides of great interest in the field of nuclear medicine for applications in diagnostic imaging and molecularly targeted radiotherapy. Their decay properties, ^99m^Tc (*t*_1/2_ = 6.01 h, γ-ray energy *E*_γ_ = 141 keV [intensity, *I*_γ_ = 89%]) and ^188^Re (*t*_1/2_ = 17.01 h, beta particle energy *E*(*β*_max_) = 2.12 MeV and *E*_γ_ = 155 keV [*I*_γ_ = 15.5%]), make them ideal candidates in developing radiotracers for single-photon emission computed tomography (SPECT) and beta-radiotherapy, respectively.^1–4^

The origins of ^99m^Tc-tricarbonyl chemistry date back to the late 1990s when researchers sought to develop ^99m^Tc complexes with well-defined and predictable coordination environments to improve the stability and versatility of ^99m^Tc-based radiopharmaceuticals.^5,6^ The tricarbonyl core, {^99m^Tc(CO)_3_}^+^, was first synthesized in 1995 by Alberto *et al.*, by using a novel approach that allowed for the generation of a stable and easily functionalized [^99m^Tc(H_2_O)_3_(CO)_3_]^+^ complex.^7^ This discovery was significant because the tricarbonyl moiety not only provided a stable framework for ^99m^Tc coordination but also enabled the facile attachment of a wide range of ligands and biomolecules, thus expanding the utility of ^99m^Tc in molecular imaging.^8–10^

Building on the success of ^99m^Tc tricarbonyl chemistry, researchers turned their attention to ^188^Re (β-emitter), a generator radionuclide with radio-therapeutic potential.^11–14^ It is often stated that the chemical properties of Tc- and Re-based radiotracers are essentially identical but the differences in reactivity, and thermodynamic trends between these two elements and the corresponding complexes, often impinge on the successful adaptation of Tc-radiochemistry for applications with ^186/188^Re.^15,16^ A striking difference of the chemical properties is visible in the different kit formulations for ^99m^Tc- and ^186/188^Re-tricarbonyl.^17,18^ The Isolink® kit by Mallinckrodt Medical B.V. contains besides Na_2_(H_3_BCO_2_), which is also present in kit formulation for ^186/188^Re, the additives sodium borate and sodium tartrate.^19^ For the Tc-radiochemistry the pH of the reaction is ∼12. In contrast, the radiosynthesis of {^188^Re(CO)_3_}^+^ is performed at pH 6.5–6.8 and contains neither sodium borate or sodium tartrate. BH_3_NH_3_ is added as an additional reducing agent and H_3_PO_4_ to adjust the pH of the kit. The reducing power of Na_2_(H_3_BCO_2_) is insufficient for the reduction of ^186/188^Re^VII^ to ^186/188^Re^I^.^14^ Blower *et al.* observed that the formation of ^186/188^Re(v)-DMSA needs an increase from 23 °C to 100 °C and prolongation of the reaction time from 15 min for the ^99m^Tc to 30 min for the ^186/188^Re compound while using the same kit formulation.^11,20^ Once isolated, the biological behavior of the ^188^Re^V^-DMSA compounds resembles ^99m^Tc^V^-DMSA.^21,22^ Recently, Cardinale *et al.* performed a human trial with ^99m^Tc/^188^Re-PSMA-GCK01.^23^ Differences in switching between ^99m^Tc and ^188^Re radiochemistry were evident and include: (i) a change in radiolabeling reaction solution pH from pH8.0–8.5 (^99m^Tc) to pH2.0–3.5 (^188^Re), (ii) a 4-fold increase in the ligand concentration for successful ^188^Re-radiolabeling, and (iii) longer reaction time (^99m^Tc: 10 min *vs.*^188^Re: 60 min). Despite the different preparative aspects, the biological behavior of both compounds was comparable.

In the domain of radiolabeled proteins, the monoclonal antibody (mAb) rituximab has been directly radiolabeled by using the ^99m^Tc- and ^188^Re-tricarbonyl cores with different approaches; the labeling experiment was attempted both on the native and on the reduced form of the mAb, yielding very different results.^24^ The {^188^Re(CO)_3_}^+^ core was less reactive on the native mAb (60% RCY at 24 h) than the corresponding ^99m^Tc complex (>95% RCY at 3 h). However, once formed, challenge assays using cysteine and histidine as competing agents revealed that the ^188^Re-rituximab remained 90% intact and biochemically active after 24 h, whereas the ^99m^Tc-rituximab adduct was unstable (<5% intact at 24 h) with most activity found unbound from the protein fraction.

These radiochemical, preclinical and clinical studies exemplify how the purported “similarities” in the chemistry of Tc and Re can, in reality, be dramatically different.

Regarding the labeling of mAbs, Ogawa *et al.* also reported direct labeling of an IgG_1_ murine mAb with the {^186/188^Re(CO)_3_}^+^ core.^25^ After heating for 2 h at 43 °C they obtained the radiolabeled mAb in RCYs from 23% to 28%. Another direct labeling approach for ^99m^Tc and ^186/188^Re utilizes the disulfide bonds of mAbs which can be reduced to the free thiol and then labeled with the radiometals.^26–28^ Antibodies can also be labeled with ^99m^Tc and ^186/188^Re by using a multistep radiosynthesis involving rhenium complexation by the mercapto acetyl triglycine (MAG_3_) ligand to give the [^186/188^ReO-(MAG_3_)]^−^ complex bearing a free carboxylic acid.^29–31^ After solvent exchange to an organic solvent and purification, the free carboxylic acid group of the [^186/188^ReO-(MAG_3_)]^−^ complex can be converted to an active ester. The activated ester was repurified, the solvent exchanged for a biocompatible buffer for the protein labeling and then the bioconjugation performed. Overall RCYs for this lengthy method showed a high variability.

In this work we aimed to adapt ^99m^Tc^I^-tricarbonyl chemistry for applications with ^188^Re and combine this tricarbonyl core with our recent development of light-induced labeling of mAbs.^32,33^ Previously, we explored light-activated protein bioconjugation with redox-inert (under biocompatible conditions) metals ions such as Ga^3+^, Zr^4+^, Lu^3+^, and In^3+^ ions.^33–37^ The use of photochemistry for radiolabeling reactions with ^89^Zr, ^11^C, and ^18^F is under investigation.^38,39^ To adapt this chemistry for use with Tc and Re, a major challenge that was overcome here was to adapt the redox-sensitive chemistry of the ArN_3_ moiety with reductive complexation methods that are required for complexation of Tc^I^ and Re^I^ metal ions. We also report detailed spectroscopic studies on the chemical speciation of the {^nat^Re(CO)_3_}^+^ in aqueous conditions, resolving a longstanding mystery in Re-based complexation chemistry.

## Results and discussion

### ^188^Re-tricarbonyl radiochemistry

**Fig. 1:**
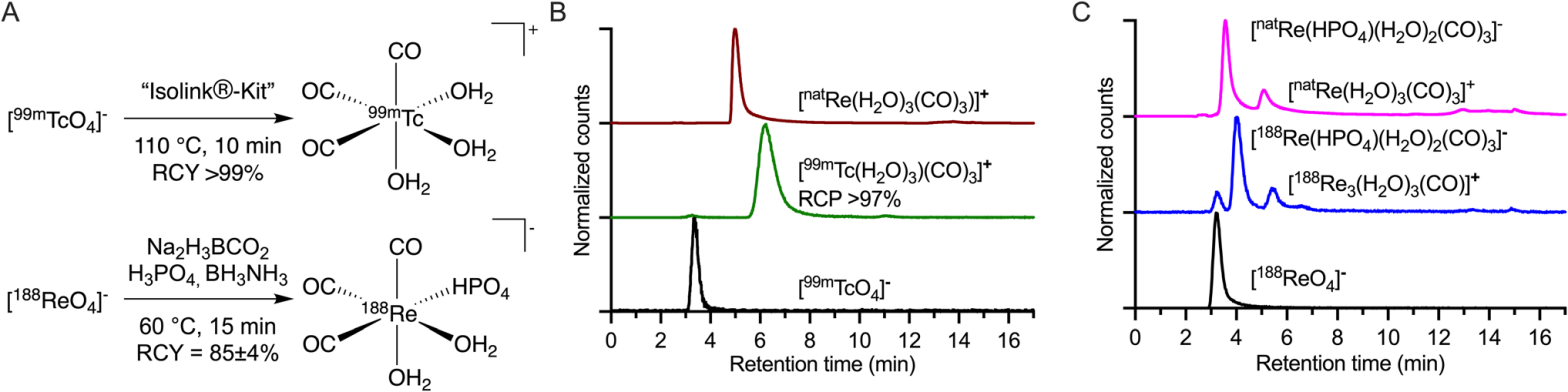
Chemical reactions for the preparation and chromatographic characterization of {M(CO)_3_}^+^ (M = ^99m^Tc, ^188^Re or ^nat^Re) species. (A) Radiosynthesis of [^99m^Tc(H_2_O)_3_(CO)_3_]^+^ and [^188^Re(HPO_4_)(H_2_O)_2_(CO)_3_]^−^ (please see ESI Schemes S1 and S2 and Fig. S1 and S2 for further details†); (B) radio-HPLC chromatograms of [^99m^TcO_4_]^−^ (black radiotrace), [^99m^Tc(H_2_O)_3_(CO)_3_]^+^ (green radiotrace), and [^nat^Re(H_2_O)_3_(CO)_3_]^+^ (red, electronic absorption trace 254 nm). (C) Equivalent radio-HPLC chromatograms obtained during the synthesis of the {^nat^Re(CO)_3_}^+^ core showing the retention times of the parent [^188^ReO_4_]^−^ (black radiotrace), the ^188^Re-reaction mixture (blue radiotrace), and the mixture formed by using [^nat^Re(H_2_O)_3_(CO)_3_]^+^ (magenta electronic absorption trace 254 nm) placed under the experimental conditions used in the ^188^Re radiochemistry. Discrete peaks for [^188^Re(HPO_4_)(H_2_O)_2_(CO)_3_]^−^ and [^188^Re(H_2_O)_3_(CO)_3_]^+^ were observed at 4.0 min and 5.5 min, respectively.

The current study focused on creating and evaluating a radiotheranostic pair based on the congener elements ^99m^Tc and ^188^Re. First, the [M^I^(H_2_O)_3_(CO)_3_]^+^ (M = ^99m^Tc or ^188^Re) precursors were prepared by starting from the [M^VII^O_4_]^−^ permetallate oxoanions (Fig. 1) following procedures adapted from Alberto and co-workers.^17,18^ Na_2_(H_3_BCO_3_) acts as the CO source for both metals. Due to the higher redox stability of ^188^Re^VII^ compared with ^99m^Tc^VII^, different reducing agents are required.^40–42^ With Tc, the CO releasing molecule Na_2_(H_3_BCO_3_) also acts as the reductant, while for ^188^Re, the stronger reductant BH_3_NH_3_ is needed. The reduction potential of BH_3_NH_3_ in alkaline media is reported as −1.216 V *vs.* SHE.^43^ For Na_2_(H_3_BCO_2_), no reduction potential is reported but as it decomposes during the reaction to [B(OH)_4_]^−^, HCOO^−^ (−0.2 V *vs.* SHE), CO (−0.11 V *vs.* SHE), and H_2_ (0 V *vs.* SHE),^44^ the redox potentials of these products can be used as a guide. The different synthetic conditions employed between Tc and Re in the preparation of {M^I^(CO)_3_}^+^ (*M* = ^99m^Tc or ^188^Re) from [M^VII^O_4_]^−^ led to the formation of different solution phase species (Fig. 1A and B). For instance, ^99m^Tc has been proven to form the *tris*-aquo complex [^99m^Tc(H_2_O)_3_(CO)_3_]^+^ identified in the reverse-phase HPLC with a retention time of 6.26 min (Fig. 1B, green radiotrace) and confirmed by standard co-elution methods against the authenticated sample of [^nat^Re(H_2_O)_3_(CO)_3_]^+^ (Fig. 1B, red trace). The standard reaction temperature and time for the ^99m^Tc synthesis of heating at 90 °C for 30 min were adjusted to 110 °C for 10 min for a shorter reaction time with identical yields.^18^ [^99m^Tc(H_2_O)_3_(CO)_3_]^+^ is a stable and versatile radiosynthetic intermediate used in the Isolink^®^ kit for the preparation of many ^99m^Tc-based radiotracers.^2,45^ In the case of ^188^Re (Fig. 1C), a mixture of two species was identified with retention times of 4.0 min (∼77%) and 5.5 min (∼23%).^17^ The minor peak eluting with a retention time of 5.5 min was previously identified as [^188^Re(H_2_O)_3_(CO)_3_]^+^ by co-injection with the ^nat^Re complex (Fig. 1C). On the other hand, the major compound found in the ^188/nat^Re-tricarbonyl synthesis has not previously been assigned.^17^ One hypothesis was that the molecule is the NH_3_ coordinated complex with unknown stoichiometry of formula [^188^Re(NH_3_)_x_(H_2_O)_3−x_(CO)_3_]^+^ but definitive characterization has remained elusive.^14^

A recent study by Williams *et al.* used gel-electrophoresis to show that the chemical properties of the carbonyl species depend on the buffer system used.^46^ The use of citrate and phosphate buffer led to a negatively charged complex while Tris buffer led to a positively charged one. Here, we assigned the species at 4.0 min (Fig. 1C) as [^188/nat^Re(HPO_4_)(H_2_O)_2_(CO)_3_]^−^ by using a combination of (radio-)HPLC, mass spectrometry, and ^31^P NMR analysis (ESI Fig. S2–S6†).

**Fig. 2:**
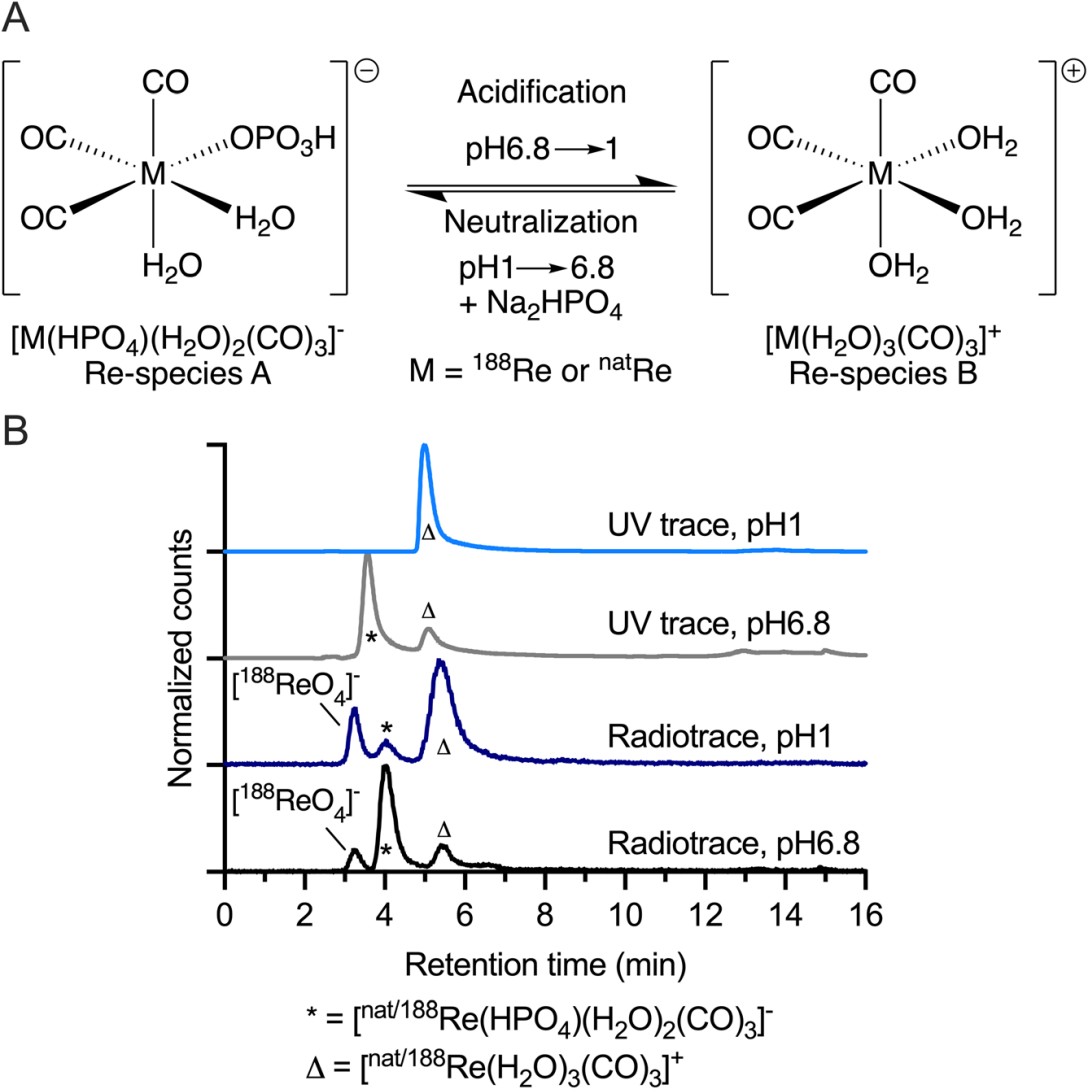
Experimental data on the aqueous-phase speciation of {^nat/188^Re(CO)_3_}^+^ showing (A) the conversion of [^nat/188^Re(HPO_4_)(H_2_O)_2_(CO)_3_]^−^ into [^nat/188^Re(H_2_O)_3_(CO)_3_]^+^ by acidification, and the reverse transformation of [^nat/188^Re(H_2_O)_3_(CO)_3_]^+^ to [^nat/188^Re(HPO_4_)(H_2_O)_2_(CO)_3_]^−^ by addition of phosphate and adjustment to pH 6.8 by using aq. NaOH. (B) Radio-HPLC chromatograms of [^188^Re(HPO_4_)(H_2_O)_2_(CO)_3_]^−^ (*, 4.0 min) and the minor species [^188^Re(H_2_O)_3_(CO)_3_]^+^ (Δ, 5.5 min) at pH 6.8 (black trace), radio-HPLC chromatograms of [^188^Re(H_2_O)_3_(CO)_3_]^+^ (5.5 min) and the minor species in [^188^Re(HPO_4_)(H_2_O)_2_(CO)_3_]^−^ (*, 4.0 min) at pH 1 in (dark blue trace), the corresponding electronic absorption (254 nm) HPLC chromatograms of ^nat^Re species placed under the radiosynthesis conditions at pH 6.8 (grey trace) showing the presence of both [^nat^Re(HPO_4_)(H_2_O)_2_(CO)_3_]^−^ (*, 3.6 min) and [^nat^Re(H_2_O)_3_(CO)_3_]^+^ (Δ, 5.1 min), and the reversible formation of [^nat^Re(H_2_O)_3_(CO)_3_]^+^ (Δ, 5.1 min) on acidification to pH 1 (light blue trace).

Our experimental data are consistent with the formation of a pH-dependent equilibrium between two identified ^nat/188^Re species in aqueous conditions in the presence of HPO_4_^2−^ anions (Fig. 2A). The ratio between [^188^ReO_4_]^−^ (10%), [^188^Re(HPO_4_)(H_2_O)_2_(CO)_3_]^−^ (70%) and [^188^Re(H_2_O)_3_(CO)_3_]^+^ (20%) after radiosynthesis is shown in Fig. 2B (black radiotrace, pH 6.8), indicating that under conditions shown in Fig. 1A, [^188^Re(HPO_4_)(H_2_O)_2_(CO)_3_]^−^ is the major species present in solution but that the reaction does not go to completion – consistent with previous observations.^17,47,48^ The composition of the mixture can be changed by acidification to pH1 (Fig. 2B, grey trace) whereby the percentage of [^188^ReO_4_]^−^ increases from ∼10% to ∼17%, and the dominant species is now [^188^Re(H_2_O)_3_(CO)_3_]^+^ at 73%. The ^nat^Re complex [^nat^Re(HPO_4_)(H_2_O)_2_(CO)_3_]^−^ was prepared by addition of 10 equivalents of NaH_2_PO_4_ to [^nat^Re(H_2_O)_3_(CO)_3_]^+^ followed by a pH adjustment to 6.5–6.8, and HPLC analysis using electronic absorption detection at 254 nm (Fig. 2B, light grey trace). Under these conditions, [^nat^Re(HPO_4_)(H_2_O)_2_(CO)_3_]^−^ was the dominant peak (retention time 3.6 min). The coordinating phosphate molecule in [^nat^Re(HPO_4_)(H_2_O)_2_(CO)_3_]^−^ is spontaneously eliminated by adjusting the pH of the reaction mixture to 1 (Fig. 2B, light blue trace), whereby only the peak assigned to [^nat^Re(H_2_O)_3_(CO)_3_]^+^ was observed at a retention time of 5.5 min. After raising the pH back to 6.5–6.8 of an aliquots of [^nat^Re(H_2_O)_3_(CO)_3_]^+^, the monophosphate coordination complex formed again with the chromatogram appearing identical to the original composition (Fig. 2B, light grey trace). It should also be noted that the radiosynthesis of the ^188^Re-tricarbonyl complex can be performed in the absence of H_3_PO_4_ by using HCl to adjust the pH of the reaction, which yields solely in the formation of the [^188^Re(H_2_O)_3_(CO)_3_]^+^ complex (ESI Scheme S3 and Fig. S7†). Analysis by high-resolution electrospray ionization mass spectrometry (HR-MS) in the negative mode provided further evidence whereby the mass of the [^nat^Re(HPO_4_)(CO)_3_]^−^ anion was detected as the sole phosphate coordinated species (ESI Fig. S3†). It is assumed that during the ionization step of the mass spectrometry analysis, two weakly bound aquo ligands can readily dissociate but in solution phase, the octahedral complex [^nat^Re(HPO_4_)(H_2_O)_2_(CO)_3_]^−^ is likely to persist.

^31^P-NMR analysis of the mixture produced with the {^nat^Re(CO)_3_}^+^ core measured in the presence of phosphate (Fig. 2B, light gray trace) revealed a multitude of species with chemical shifts in the range of 6.28 to 2.41 ppm (ESI Fig. S4A†). Similar observations were made by Williams *et al*.^46^ The addition of 200 equivalents of phosphate at pH 6.5–6.8 to an aliquot of [^nat^Re(HPO_4_)(H_2_O)_2_(CO)_3_]^−^ led the solution phase mixture to converge towards one main species with a resonance peak at 5.05 ppm, and two minor ones with resonance peaks at 5.78 ppm and 3.71 ppm (ESI Fig. S4B†). By acidifying an aliquot of this mixture with aq. 1 M HCl (ESI Fig. S4C†) or measuring the ^31^P-NMR of an aliquot at 60 °C (ESI Fig. S4D†), the previously observed species vanished and only the resonance of free phosphate remained. The temperature dependent ^31^P-NMR suggests an exothermic binding of the phosphate ligands. By mixing the [^nat^Re(H_2_O)_3_(CO)_3_]^+^ complex (Fig. 2B, light blue trace) with 200 equivalents NaH_2_PO_4_ at pH 4.4, two new resonances in the ^31^P-NMR spectrum at 3.92 and 3.60 ppm were visible, whereas by mixing with Na_2_HPO_4_ at pH9.6 two resonances remain, a sharp one at 4.70 ppm and a broad one at 2.41 ppm, in addition to free phosphate, were visible (ESI Fig. S4E and S4F,† respectively). The ^31^P-NMR data indicate a complex speciation pattern exists under the aqueous-phase conditions tested. It is not clear how the observations of a singular species in the (radio)-HPLC chromatogram can be matched with the observations in the NMR experiments but it is plausible that under the chromatographic conditions either one species predominates, or that the multiple Re-phosphate species observed in NMR co-elute.

**Fig. 3:**
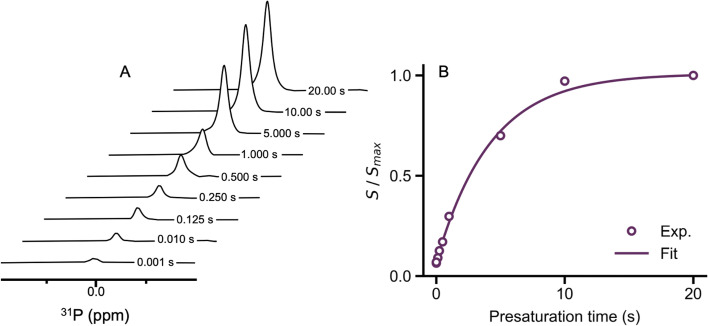
Time evolution of the ^31^P-NMR spectra (202 MHz, H_2_O : D_2_O, phosphate buffers) measuring the exchange rate of PO_4_^3−^ with presaturated [^nat^Re(HPO_4_)(H_2_O)_2_(CO)_3_]^−^. (A) Difference spectra highlighting the change in the intensity of the free phosphate peak found at 0.0 ppm with respect to presaturation time. (B) Integral of the signals found in (A) plotted against the presaturation time of the bound phosphate (in seconds). The solid line represents the best fit to model given by ESI eqn (5).†

Motivated by the new findings in the ^31^P-NMR spectroscopy, we investigated the phosphate binding reaction by using spin saturation transfer difference NMR (SSTD NMR) experiments – similar to the methods reported by Muñoz *et al.*^49,50^ First, we determined the binding equilibrium constant at 298 K (*K*_eq_ = 1.41) measured by virtue of the slow NMR exchange regime at 202 MHz for ^31^P, then we measured the exchange kinetics between bound and free phosphate by using a dynamic experiment. In brief, the singlet of the bound phosphate at 5.83 ppm (ESI Fig. S5†) was saturated for a given amount of time and the peak of the free phosphate was detected immediately after presaturation (Fig. 3A). The time evolution of the area under the signal is indicative of the magnetization residing on the free phosphate, which is directly connected to the exchange rate constant between free and bound phosphate. Fitting of these data to a pseudo first-order rate equation and accounting for the concentration of the reactive partners gave an observed rate constant for phosphate exchange of *k*_obs_ = 0.24 ± 0.02 M^−1^ s^−1^ (Fig. 3B).

The *k*_obs_ parameter is a convolution between the forward (*k*_1_ = 0.133 ± 0.013 M^−1^ s^−1^) and reverse (*k*_−1_ = 0.107 ± 0.012 M^−1^ s^−1^) rate constants. These parameters are calculated from the law of mass action applied on *K*_eq_ together with the definition of *k*_obs_ (ESI eqn (6) and (7)†) and are comparable with previous works on ligand exchange kinetics on [^nat^Re(H_2_O)_3_(CO)_3_]^+^ carried out by Alberto and co-workers, and summarized by Helm.^51,52^

### Synthesis of a bifunctional photoactivatable ligand for {M(CO)_3_}^+^ (M = ^99m^Tc, ^188/nat^Re) complexation

**Scheme 1:**
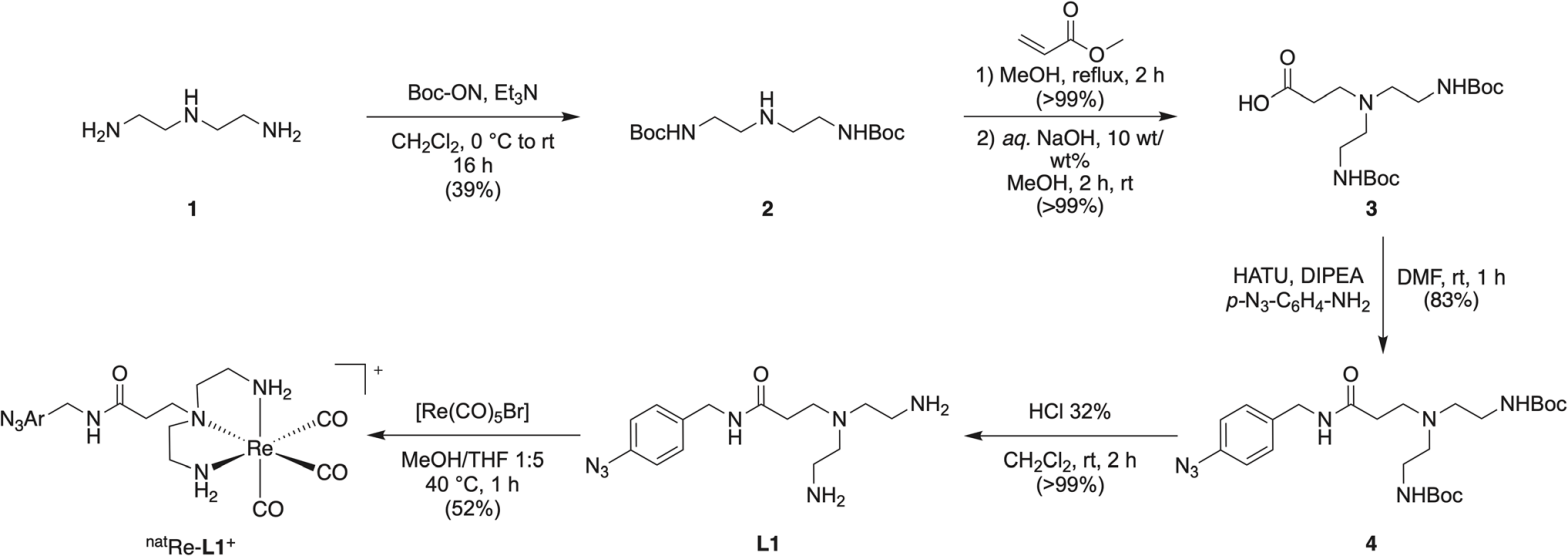
Synthesis scheme of the ligand L1 and the complex ^nat^Re-L1^+^ starting from diethylenetriamine 1.

The bifunctional ligand DETA-ArN_3_ (L1) was synthesized starting from *N*^1^-(2-aminoethyl)ethane-1,2-diamine (compound 1; Scheme 1). The synthesis is shown in detail in the ESI Schemes S4–S10 and analytics Fig. S11–S32.† The triamine-binding scaffold was chosen based on the research of Rattat *et al.* who compared various N, O, and S donor sets.^53^ First, compound 1 was Boc protected at the primary amine groups to yield compound 2. Michael-addition on the central nitrogen atom of compound 2 using methyl acrylate, followed by saponification of the methyl ester, gave compound 3 in >99% yield. Amide bond formation by using 4-azidobenzylamine (compound 9) and a HATU coupling introduced the photoactivatable aryl azide into our ligand scaffold yielding compound 4 in 83% yield. Removal of the Boc protecting groups with conc. HCl revealed the free primary amines, furnishing the desired *tris*-amine ligand L1 in 5 steps, with an overall yield of 32% and a purity over 99% as determined by HPLC and ^1^H-NMR spectroscopy.

**Fig. 4:**
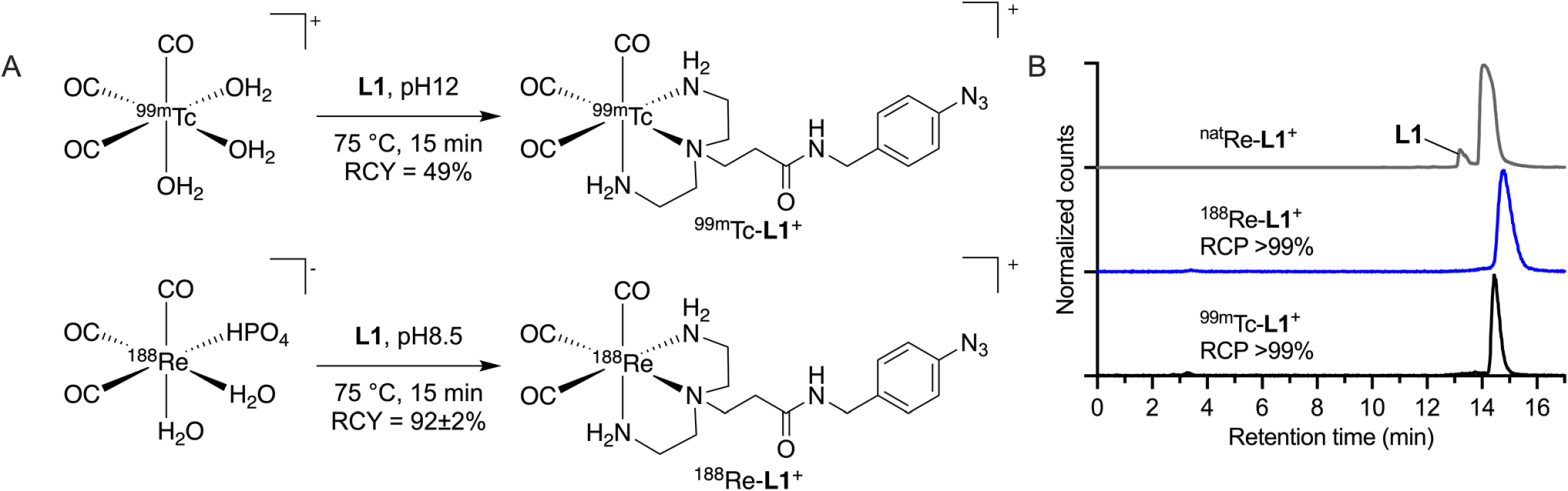
Radiosynthesis of the M-L1^+^ complexes (M = ^99m^Tc, ^188/nat^Re) showing (A) the synthetic procedures employed and (B) (radio-)HPLC chromatograms of ^99m^Tc-L1^+^, ^188^Re-L1^+^, and ^nat^Re-L1^+^ (UV trace, 254 nm).

The non-radioactive reference complex [^nat^Re(CO)_3_(DETA-ArN_3_)]^+^ (^nat^Re-L1^+^) was synthesized by reacting the readily available starting material [^nat^Re(CO)_5_Br] with ligand L1 in a 1 : 5 v/v mixture of MeOH and THF at 40 °C for 1 hour (Scheme 1 and ESI Scheme S11†). The identity of the ^nat^Re complex was confirmed by NMR, HPLC, and HR-MS (ESI Fig. S33–S37†). Radiolabeling L1 with [^99m^Tc(H_2_O)_3_(CO)_3_]^+^ was performed with a ligand concentration of 0.3 mM at pH 12 (Fig. 4A) yielding the radiolabeled complex [^99m^Tc(CO)_3_(DETA-ArN_3_)]^+^ (^99m^Tc-L1^+^) in high radiochemical conversion (RCC, >99%) and radiochemical purity (RCP, >99%) as analyzed by radio-HPLC (Fig. 4B, black trace). For further details, see ESI Scheme S12 and Fig. S38.† Upon purification by a C8-cartridge which is described in detail in the ESI Scheme S12,† the complex ^99m^Tc-L1^+^ was obtained in an RCP > 99% and RCY of 49%. The retention time of ^99m^Tc-L1^+^ measured under the same conditions as the precursor, shifted to 14.55 min, indicating an increase in lipophilicity due to the ligand. Co-injection with the authenticated ^nat^Re-L1^+^ complex confirmed the identity of the radioactive species (Fig. 4B, grey trace).

Radiosynthesis of the corresponding ^188^Re-L1^+^ complex started from the mixture of [^188^Re(HPO_4_)(H_2_O)_2_(CO)_3_]^−^ and [^188^Re(H_2_O)_3_(CO)_3_]^+^ produced at pH 6.8 (see ESI Scheme S13 and Fig. S39†) and used the ligand L1 at 10 mM concentration (Fig. 4A). The reaction yielded a single species, identified as the desired complex ^188^Re-L1^+^ (Fig. 4B, blue trace). Similar to the ^99m^Tc labeled complex, the retention time of ^188^Re-L1^+^ shifted to 14.85 min. Identity of the radioactive complex was confirmed by co-injection with ^nat^Re-L1^+^ (Fig. 4B, grey trace). The shift in the retention times of the ^nat^Re and ^188^Re labelled complex is due to the serial arrangement of the UV- and radio-detector. Upon purification by a C8-cartridge eluted with a 1 : 4 v/v mixture of acetone/ethanol (ESI Fig. S40†), the ^188^Re-L1^+^ complex was obtained in an RCP > 99% and RCY of 92 ± 2% (*n* = 2). Here we note that the varying reactivities of the Tc and Re metal ions are evident leading to an increased ligand requirement, *viz.* 30-fold increase for the 5d metal compared with the 4d metal, to drive the ^188^Re-radiochemical conversion toward completion.

### Radiochemical stability studies on the [^99m^Tc/^188^Re-L1]^+^

The radiochemical stability with respect to ligand dissociation or complex degradation of purified samples of ^99m^Tc-L1^+^ and ^188^Re-L1^+^, free of excess ligand (as proven by HPLC measurements, ESI Fig. S40†), were tested in the following two challenge conditions: (i) PBS at pH 7.4 at 37 °C for 24 h, and (ii) a mixture containing 1 mM cysteine and 1 mM histidine at 37 °C, pH 7.4 for 24 h (ESI Fig. S41 and S42†). Note, for ^188^Re the measurements were extended to 72 h. Cysteine and histidine were chosen as challengers since both offer a suitable donor set for the tricarbonyl core, are abundant *in vivo*, and may also be present in formulation buffers or as reactive amino acid residue on protein.^54^ Analysis by radio-HPLC of ^99m^Tc-L1^+^ and ^188^Re-L1^+^ showed no oxidative/hydrolytic degradation in PBS as confirmed by the absence of [^99m^TcO_4_]^−^ or [^188^ReO_4_]^−^. In addition, no evidence for ligand exchange was observed with histidine or cysteine indicating that both the ^99m^Tc-L1^+^ and ^188^Re-L1^+^ complexes are of suitable stability for further use in the development of radiolabeled proteins.

### Electronic absorption (UV/vis) analysis and photodegradation studies

The electronic absorption characteristics and photodegradation kinetics of the ligand L1, the photoactive complex ^nat^Re-L1^+^, and the non-photoactive complex [^nat^Re(CO)_3_(diethylenetriamine)]^+^ (used as a control; for the synthesis and characterization see ESI Scheme S14 and Fig. S43 and S44†) were analyzed by UV/vis spectroscopy (ESI Fig. S45†). The coordination of the {Re(CO)_3_}^+^ core to the ligand L1 induces a hyperchromic shift with an increase in molar absorption coefficient (*ε*/M^−1^ cm^−1^) at 255 nm from 7.6 × 10^3^ to 10.8 × 10^3^ M^−1^ cm^−1^. The molar absorption coefficients, determined by a dilution series (ESI Fig. S46 and S47†), for the ligand L1 and the ^nat^Re-L1^+^ complex were determined at 255, 283 and 290 nm and are 7.6 × 10^3^, 1.8 × 10^3^ and 1.4 × 10^3^ M^−1^ cm^−1^ for the ligand and 10.8 × 10^3^, 2.7 × 10^3^ and 2.0 × 10^3^ M^−1^ cm^−1^ for the complex (ESI Fig. S48 and S49†). The ^nat^Re-L1^+^ complex showed an overall hyperchromic shift between 200–350 nm compared with the electronic absorption spectrum of the non-photoactive complex [^nat^Re(CO)_3_(diethylenetriamine)]^+^, indicating that the spectroscopic features in this wavelength range are likely associated with the ArN_3_ moiety.

Subsequently, the rate constants of the photolysis at 395 nm of L1 and ^nat^Re-L1^+^ were determined by comparing the relative change of concentration of the starting reagent *versus* time (ESI Fig. S50†). The photolysis was done at a concentration of 0.1 mM, 25 °C and in water. Data obtained were fitted with a first-order exponential and the rate constant of the photodegradation at 395 nm was determined as *k* = 0.76 ± 015 min^−1^ for ^nat^Re-L1^+^ and 0.63 ± 0.15 min^−1^ for the ligand L1. The increase in photolysis rate constant observed for the ^nat^Re-L1^+^*versus*L1 (∼1.21 increase) is consistent with the difference in molar absorption coefficients observed on complexation of Re^I^ ions (∼1.45 increase).

As metal-carbonyl bonds are possibly susceptible to photolysis under UV-light irradiation,^55^ we also investigated the photolytic stability of the ^nat/188^Re-tricarbonyl core by exposing the model complex [^nat/188^Re(CO)_3_(diethylenetriamine)]^+^ (for the synthesis and characterization of the ^188^Re-labeled complex see: ESI Scheme S15 and Fig. S51†) to 395 nm light. No photodegradation was observed (ESI Fig. S52 and S53†).

### Photoradiosynthesis of labeled proteins using ^99m^Tc/^188^Re-L1^+^

**Fig. 5:**
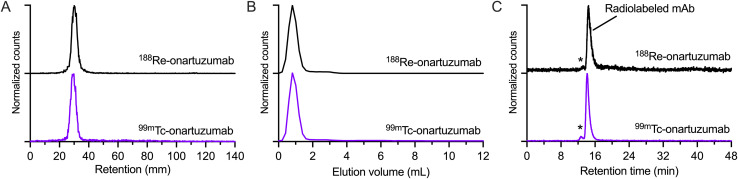
Chromatographic data on the photoradiosynthesis of ^99m^Tc-onartuzumab (purple traces) and ^188^Re-onartuzumab (black traces) showing (A) iTLC chromatograms, (B) elution profiles recorded from manual elution of a PD-10 size-exclusion gel column, and (C) SEC-HPLC radiochromatograms for the purified samples of each radiotracer. Note ‘*’ indicates the elution of high molecular weight protein aggregates (dimers and higher order multimeric species). Equivalent chromatograms of the crude reaction mixtures are presented in ESI (Fig. S62–S64 and S65–S67†).

The photochemical protein ligation using ArN_3_-based ligands was performed as previously reported in our group (Fig. 5).^32^ Thorough optimization of the photoradiolabeling conditions has been performed previously in our group.^32^ With this procedure, the ArN_3_ group is photoactivated and forms an electrophile which reacts chemoselectively with the abundant lysine residues that are available on the protein surface.^56^ The major byproduct of the photoreaction results from hydrolysis of the ketenimine ring to form the azepin-2-ol, which is removed efficiently from the radiolabeled antibody fraction by manual SEC.^57^ Extensive mechanistic data, theoretical calculations and data can be found in our previous publications.^33,56^ A photograph of the reaction setup is shown in ESI Photograph S1.†

The photoradiolabeling was first tested on the model protein human serum albumin (HSA) with ^188^Re-L1^+^ (ESI Scheme S16†). HSA (100 μL) with an initial protein concentration of 60 mg mL^−1^ in water was mixed with ^188^Re-L1^+^ (∼10 MBq) in 0.025 M borate buffer (pH 8–8.1, 250 μL) and irradiated with 395 nm light at 23 °C for 15 min. The crude reaction mixture was analyzed by instant thin-layer chromatography (iTLC) which showed a peak at *R*_f_ = 0 indicating protein bound activity (ESI Fig. S54†). Protein binding was further evidenced by analysis of the crude reaction mixture with manual size-exclusion PD-10 chromatography which gave a peak in the 0.0 to 1.6 mL fraction giving an RCC ∼ 65% (ESI Fig. S55†). To fully elute the byproducts which formed during the reaction an elution with 22 mL was necessary (ESI Fig. S56†). In addition, analysis by automated size-exclusion chromatography (SEC-HPLC) showed a signal that coincided with the retention time of HSA at about 15 min (ESI Fig. S57†). A dark control reaction of ^188^Re-L1^+^ with HSA which was analysed by PD-10 SEC showed no unspecific binding or non-light mediated binding (ESI Fig. S58†). As a further control, an analytical PD-10 SEC chromatogram was obtained for the complex and the photolysis products of ^188^Re-L1^+^ and for [^99m^TcO_4_]^−^ and [^188^ReO_4_]^−^ (ESI Fig. S59 and S60†). The tested small molecules elute from 3 mL upwards.

The isolated decay-corrected radiochemical yield (RCY) of ^188^Re-HSA was 63 ± 3% (*n* = 3) and the lower limit of the molar activity of the product (estimated by assuming no protein losses during the reaction and purification steps) was ∼0.073 MBq nmol^−1^ of protein, with an activity concentration of 4.109 MBq mL^−1^. The radiochemical purity of the purified samples of ^188^Re-HSA was estimated to be >99% (measured independently by PD-10 SEC and SEC-HPLC). The stability of ^188^Re-HSA with regard to loss of protein bound activity was evaluated in PBS at 37 °C for up to 72 h and was found to 71 ± 2% (*n* = 3) stable (ESI Fig. S61A†). Analysis of the degradation products in ESI Fig. S61B† showed various small molecules with shorter retention times than the parent ^188^Re-L1^+^ complex. The major photolytic byproduct observed gave a retention time of *t* = 13.80 min compared to the un-photolyzed ^188^Re-L1^+^, complex, *t* = 14.85 min. This major byproduct from the photolysed reaction is tentatively assigned to the hydrolysis of the ketenimine intermediate to give the azepin-2-ol derivative, consistent with previously observed data using related radiotracers featuring aryl azides as reactive handles.^33,57^ The procedure was then adapted for the labeling of the monovalent (one-armed) engineered monoclonal antibody onartuzumab.

**Scheme 2:**
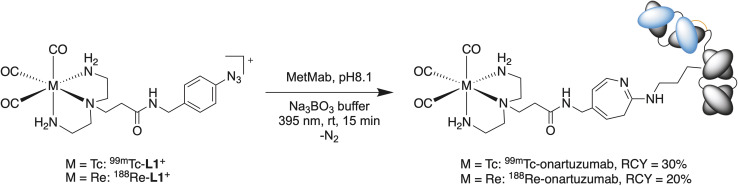
Photoradiosynthesis of ^99m^Tc- and ^188^Re-onartuzumab from ^99m^Tc- or ^188^Re-L1^+^, respectively.

Onartuzumab (formulated in the clinical-grade mixture as MetMAb, Genentech/Roche)^58^ was functionalized directly with the ^99m^Tc/^188^Re-L1^+^ complexes by irradiation with 395 nm light for 15 min at room temperature and a pH 8.1 in the presence of 0.1 M sodium borate buffer (Scheme 2). The protein concentration in the separate reaction mixtures was 15 and 14 mg mL^−1^ for ^99m^Tc-onartuzumab and ^188^Re-onartuzumab, respectively. Analysis by iTLC showed a peak at *R*_f_ = 0 for both ^99m^Tc-onartuzumab and ^188^Re-onartuzumab indicating successful protein radiolabeling (Fig. 5A). Further chromatographic analysis of the crude photolabeling reactions confirmed protein-bound activity as evidenced by a peak in the manual size-exclusion PD-10 chromatograms in the 0.0 to 1.6 mL range (Fig. 5B), and by a signal in the SEC-HPLC radiotrace that coincided with the retention time of onartuzumab at about 14.1 min (RCC: ∼30% for ^99m^Tc, ∼20% for ^188^Re). The stated fast reaction under mild conditions prevent aggregation (<5% for both nuclides, Fig. 5C marked with *).

Purification of the radiolabeled antibody was achieved by passing the crude solution through PD-10 columns, eluting with sterile PBS and collecting the first 1.6 mL (high molecular weight). Aliquots of the crude and purified samples were retained and quality control was performed by iTLC, analytical PD-10 SEC and SEC-HPLC (Fig. 5A, B, and C, respectively). From these experiments, we observed that ^99m^Tc-onartuzumab and ^188^Re-onartuzumab behave the same in these chromatographic separations. The photochemical bioconjugation gave higher yields for ^99m^Tc compared with ^188^Re (30% and 20%, respectively). The lower limit of the molar activity of ^99m^Tc- and ^188^Re-onartuzumab (estimated by assuming no protein losses during the reaction and purification steps) was ∼4.146 MBq nmol^−1^ and ∼1.026 MBq nmol^−1^ of protein, with an activity concentration of 77.74 MBq mL^−1^ and 11.54 MBq mL^−1^ for ^99m^Tc-onartuzumab and ^188^Re-onartuzumab, respectively. Next, the biological behavior of the radioimmunoconjugates was evaluated *in vitro*, and *in vivo* by using xenograft models of human gastric adenocarcinoma with onartuzumab.

### Radiochemical stability and cellular binding of ^99m^Tc/^188^Re-onartuzumab

Purified samples of ^99m^Tc-onartuzumab and ^188^Re-onartuzumab were incubated in PBS at pH 7.4 at 37 °C for up to 24 h and 72 h, respectively (ESI Fig. S68 and S69†). Both radiolabeled mAbs were found to be over 70% stable with respect to loss of the radionuclide from the protein fraction during the observation window. Analysis of the degradation products of ^99m^Tc-onartuzumab by radio-HPLC, ESI Fig. S68B,† showed a small molecule with a shorter retention time of *t* = 13.68 min compared to the un-photolyzed parent complex ^99m^Tc-L1^+^ which had a retention time of *t* = 14.55 min. As discussed above, we believe the most likely product formed after photolysis involved hydrolysis of the ketenimine electrophile to give the azepin-2-ol species which has been identified in previous studies on related aryl azide radiotracers.^33,57^

**Fig. 6:**
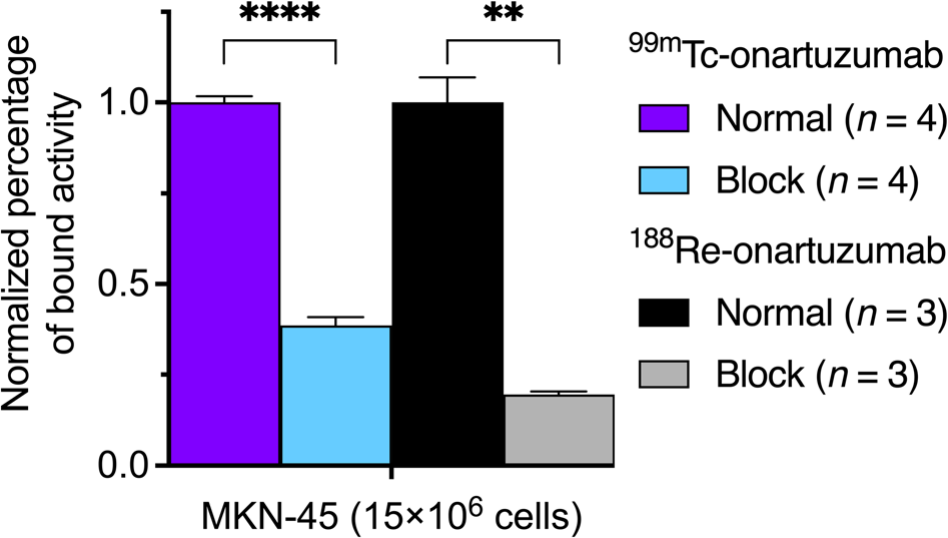
Comparison of the specific cell-bound fraction of ^99m^Tc- and ^188^Re-onartuzumab on MKN-45 cells.

The binding and specificity of ^99m^Tc-onartuzumab and ^188^Re-onartuzumab to the target protein was evaluated *in vitro* by using intact cell binding assays with human hepatocyte growth-factor receptor (c-MET) positive and overexpressing MKN-45 gastric adenocarcinoma cells (Fig. 6). Cellular binding studies confirmed that both ^99m^Tc-onartuzumab and ^188^Re-onartuzumab retained their biochemical activity and displayed specificity towards c-MET expression with 38 ± 1% (*n* = 4) and 35 ± 2% (*n* = 3) fractional binding (ESI Fig. S70 and S71†). The comparatively low values are associated with the monovalent structure of the onartuzumab and with the use of an aged ex-clinical sample of the protein, but are consistent with previous experimental studies.^35,59^ Blocking was possible by using a 100-fold excess of non-radioactive mAb, and the assay yielded a 2.5-fold and 5-fold decrease in binding for the ^99m^Tc- and ^188^Re-onartuzumab immunoconjugates, respectively. These data confirmed the retention of bioactivity and specificity following radiolabeling of the protein and indicated that the radiotracers are suitable for further evaluation using animal models of human c-MET positive tumors.

### Imaging and biodistribution studies of ^99m^Tc- and ^188^Re-onartuzumab in animal models

Time-dependent γ-scintigraphy imaging was used to measure the distribution of ^99m^Tc-onartuzumab and ^188^Re-onartuzumab in female athymic nude mice bearing subcutaneous MKN-45 xenografts on the right flank/shoulder. In addition, two groups of animals received a low molar activity formulation of the same batch of photoradiolabeled ^99m^Tc-onartuzumab or ^188^Re-onartuzumab doped in each case with a 100-fold molar excess of non-radioactive MetMAb. These low molar activity formulations were used as control blocking groups to measure the specificity of tumor uptake *in vivo*.

In the normal groups, the mice received an activity dose of 1.750–1.811 MBq for ^99m^Tc for the 24 h time point, and activity doses of 0.556–0.562 MBq or 0.621–0.712 MBq for ^188^Re for the 24 h and 72 h time points, respectively. The normal doses contained a total protein mass of 50 μg in 150 μL of sterile PBS which was administered by intravenous (i.v.) tail-vein injection. For the blocking groups, the mice received an activity dose of 1.686–1.762 MBq for ^99m^Tc for the 24 h time point, and activity doses of 0.571–0.629 MBq or 0.601–0.699 MBq for ^188^Re for the 24 h and 72 h time points, respectively. The block doses with reduced molar activity contained a total protein mass of ∼1.05 mg, also administered by i.v. tail-vein injection as a bolus injection in 150 μL of sterile PBS.

**Fig. 7:**
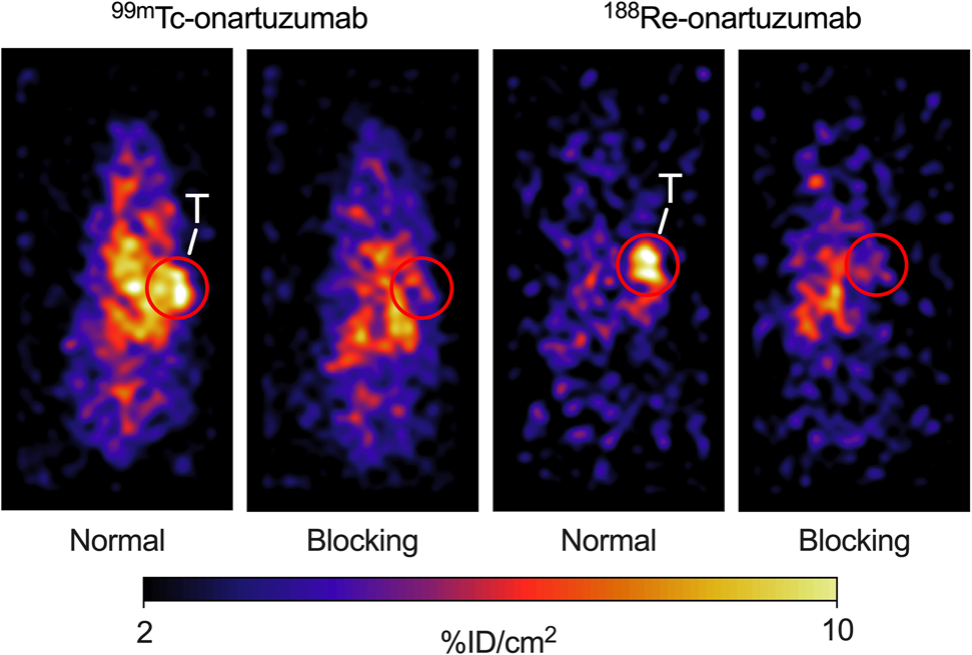
Representative, planar γ-scintigraphy images recorded in athymic nude mice bearing MKN-45 tumors on the right flank at 24 h post-administration of the radiotracers for (left) ^99m^Tc-onartuzumab (normal: 1.750–1.811 MBq, blocking: 1.686–1.762 MBq) and (right) ^188^Re-onartuzumab (normal: 0.556–0.562 MBq, blocking: 0.621–0.721 MBq). The mice received 50 μg or ∼1.05 mg of protein in the normal or blocking groups respectively. T = tumor.

Planar γ-ray scintigraphy images were recorded 24 h post-administration (Fig. 7). Radiotracer localization in the tumor is evident for the normal groups for ^99m^Tc- and ^188^Re-onartuzumab. The difference in contrast shown by these two-dimensional images is presumably due to the different geometry of the tumors (distance to the detector), low counting statistics for ^188^Re (*I*_γ_ = 15%) compared with ^99m^Tc (*I*_γ_ = 89%), and does not represent a real difference as confirmed with the following biodistribution data for ^99m^Tc- and ^188^Re-onartuzumab (*vide infra*). Nevertheless, tumor specificity was demonstrated by the reduction in signal intensity observed between the images recorded for animals assigned to the normal and blocking groups. The time progression of the distribution of the radioactive mAb between 0 and 24 h was acquired for one mouse belonging to each group (ESI Fig. S72 and S73†).

**Fig. 8:**
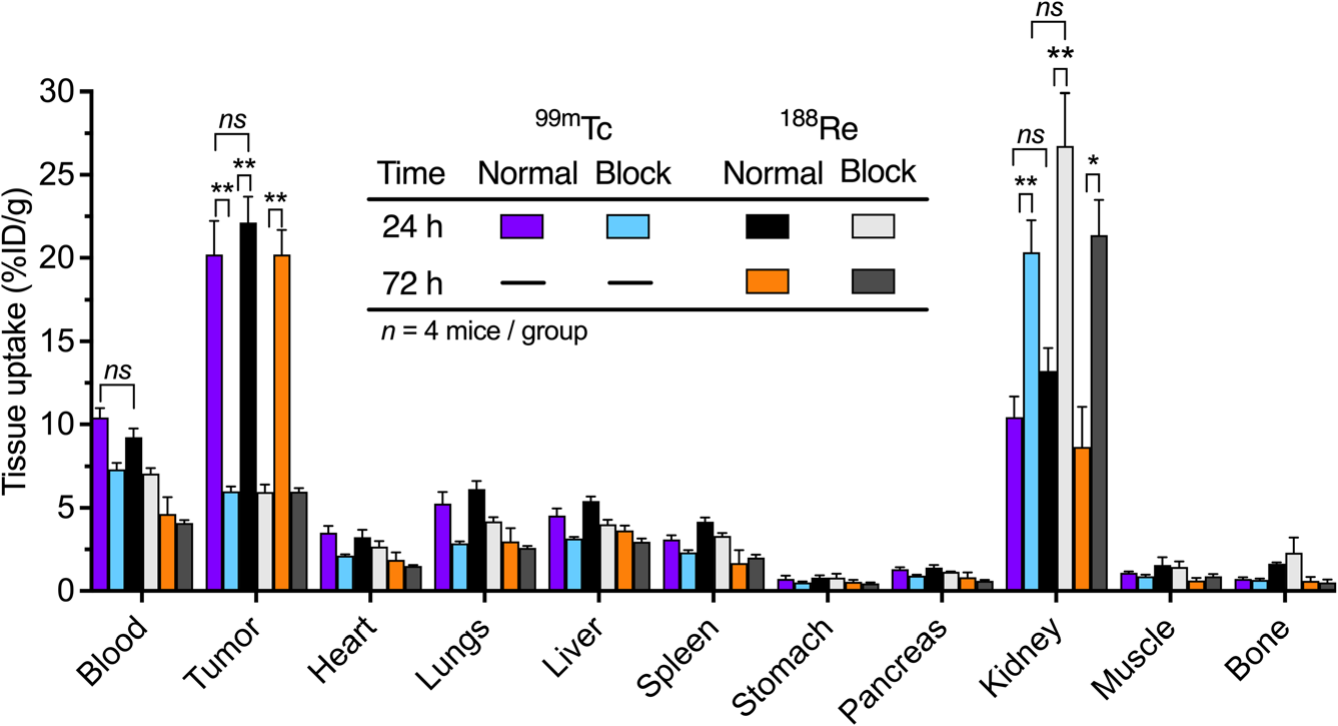
Bar chart showing *ex vivo* biodistribution data (% ID g^−1^) for the tissue uptake of ^99m^Tc-onartuzumab (normal and blocking groups, purple and light-blue grey bars [24 h], respectively) and ^188^Re-onartuzumab (normal and blocking groups, black and white bars [24 h]; orange and dark grey bars [72 h], respectively) in mice bearing MKN-45 tumors at 24 h and 72 h after injection. ESI Fig. S74 and S75† provides additional information. Student's *t*-test: *P < 0.05, **P < 0.01, ns = not significant.

The first time point for biodistribution analysis was acquired at 24 h to facilitate a direct comparison between the distribution profiles of ^99m^Tc-onartuzumab and ^188^Re-onartuzumab. In addition, a later time point of 72 h was used to study temporal changes in the tracer uptake/retention of the longer-lived ^188^Re-onartuzumab. In each case, normal and blocking groups were measured. At the specified time points, animals were euthanized after 24 h for the ^99m^Tc and ^188^Re groups, and after 72 h for the ^188^Re group by exsanguination *via* cardiac puncture after isoflurane overdose and 15 tissues were harvested for activity quantification of both ^99m^Tc and ^188^Re (Fig. 8 and ESI Fig. S74 and Tables S2 and S3†). A comparison between the normal groups for ^99m^Tc/^188^Re at 24 h post-radiotracer administration showed no significant difference (two groups of *n* = 4, *P* = 0.7) in tumor uptake. These values were compared with the corresponding blocked groups at 24 h for ^99m^Tc and ^188^Re showing a significant difference (*P* = 0.005 ^99m^Tc, 0.003 ^188^Re) which confirmed the specificity of tumor targeting *in vivo*. The difference between tumor uptake between the ^99m^Tc and ^188^Re blocked groups at 24 h was not significant (*P* = 0.9). Comparison between normal and blocked group for ^188^Re at the 72 h time point highlighted the retention of both tumor-associated activity and target specificity for ^188^Re-onartuzumab (*P* = 0.002 normal *versus* blocking groups).

No statistically significant differences were observed in the measured tumor-to-tissue contrast ratios at 24 h between ^99m^Tc-onartuzumab and ^188^Re-onartuzumab for the normal groups (ESI Fig. S76†). Kidney uptake at 24 h for ^99m^Tc/^188^Re, and at 72 h for ^188^Re, between the normal and blocking groups increased. This is a known dose-dependent phenomenon associated with the monovalent onartuzumab engineered antibody and has been observed with many different radionuclides, chelates, and conjugation strategies in the same MKN-45 xenograft models.^32,35,59^ Uptake in the liver and spleen was low (∼5%) for the normal and blocking groups at all time points, and for both radionuclides, indicative of the high chemical stability of the radiotracers *in vivo* and the low degree of protein aggregates formed by the photoradiolabeling methods used.

The effective and biological half-life modeled with a one-phase decay showed high similiarty for the normal and blocking groups of ^99m^Tc-/^188^Re-onartuzumab (ESI Fig. S77 and S78†). The half-life measurements for ^188^Re-onartuzumab were also modeled with a two phase decay, corresponding to a faster wash-out phase within the first 22 h after administration, and a second slower phase observed from 17 h up to 72 h (ESI Fig. S77A and B†).

Overall, the biochemical data collected from cellular and animal experiments showed the biochemical equivalence of the photoradiolabeled ^99m^Tc-onartuzumab and ^188^Re-onartuzumab radiotracers. These observations support the further development of ^188^Re-based radiolabeled mAbs for applications in radioimmunotherapy, and of the concurrent use of ^99m^Tc-mAb radiotracers as diagnostic SPECT agents for establishing dosimetry profiles.

In this work, the differences in reactivity for ^99m^Tc and ^188^Re are most striking in the synthesis of the tricarbonyl precursors as they yield different species, [^99m^Tc(H_2_O)_3_(CO)_3_]^+^ and [^188^Re(HPO_4_)(H_2_O)_2_(CO)_3_]^−^ for ^99m^Tc and ^188^Re, respectively. Nevertheless, upon introduction of the *tris*-amine ligand L1, both metal complexes transform to the analogous complexes [M(CO)_3_L1]^+^ (*M* = ^99m^Tc or ^188^Re). The different reactivities are again evident as a 30-fold increase in ligand concentration was necessary for ^188^Re complexation compared with ^99m^Tc.

In summary, we conclude that only marginal differences were observed between the performance of the ^99m^Tc- and ^188^Re-onartuzumab radiotracers *in vivo*. Provided that the radiometal ion complexes remain stable to challenge studies, and the synthetic methods used do not reduce the biological integrity of the protein, it appears that {M(CO)_3_}^+^ chemistry is suitable for developing antibody-based ^99m^Tc- and ^188/186^Re-radioimmunoconjugates a theranostics pairs.

## Conclusion

We introduce new methods for the effective complexation and photoradiosynthesis of ^99m^Tc- and ^188^Re-proteins using the {M(CO)_3_}^+^ core. The identity of the previously unknown aqueous-phase tricarbonyl complex of ^188^Re was determined which elevates the understanding of the ^188^Re chemistry. The novel photoactivatable complexes ^99m^Tc-L1^+^ and ^188^Re-L1^+^ were synthesized in two steps from [^99m^TcO_4_]^−^ and [^188^ReO_4_]^−^. ^99m^Tc- and ^188^Re-onartuzumab were produced *via* a light-induced photochemical process using the aryl azide (ArN_3_) chemistry. The light-activated bond formation with the protein marks an important step for the radiolabeling of complex biomolecules like mAbs, where previously, the harsh reaction conditions required for labeling with ^188^Re did not allow the use of redox-sensitive groups for bioconjugation such as maleimides, isothiocyanates, or activated esters.^25,26,29,60^ The photochemical protein ligation reaction allows a simple and efficient conjugation of the ^99m^Tc/^188^Re-tricarbonyl complexes to the mAb starting from the fully formulated antibody mixture, which is practical for clinical use. The data presented provide encouraging evidence that support the further development of ^99m^Tc/^188^Re-radiolabeled proteins as radiotheranostics.

## Materials and methods

Full details of all experimental methods, syntheses, characterization data, and equipment used are presented in the ESI.†

## Ethical statement

All experiments involving mice were conducted in accordance with an animal experimentation license approved by the Zurich Canton Veterinary Office, Switzerland (Jason P. Holland).

## Data availability

The data supporting this article have been included as part of the ESI.†

## Author contributions

The study was conceptualized by JG, CSB, and JPH. JG conducted the majority of experiments including the synthesis, characterization, radiochemistry, cellular studies and animal experiments. CSB and JG generated the ^31^P NMR data. JG and SaK generated the UV/vis data. SiK and JPH assisted with the animal experiments. The first draft of the manuscript was written by JG. CSB and JPH contributed to the manuscript review, editing, and data presentation. All authors approved the final submission.

## Conflicts of interest

No potential conflicts of interest relevant to this article exist.

## Supplementary Material

SC-OLF-D4SC08089K-s001
