# Supplementary material for: Photoradiolabeling of onartuzumab with 99mTc and 188Re-tricarbonyl for radiotheranostics of gastric cancer
Source: Chem Sci. 2025 Mar 13;16(15):6219–30. doi: 10.1039/d4sc08089k (PMC11924949; doi:10.1039/d4sc08089k)
Supplement: SC-016-D4SC08089K-s001 [file SC-016-D4SC08089K-s001.pdf]

Supplementary Information (SI) for Chemical Science.
This journal is © The Royal Society of Chemistry 2025

Supporting Information

Photoradiolabeling of onartuzumab with ^{99m}Tc and ^{188}Re-tricarbonyl for radiotheranostics of gastric cancer

Jonas Genz,[1] Cesare Berton,[1] Samy Kichou,[1] Simon Klingler,[1] Mirja C. Nolff,[2] Henrik Braband,[1] Jason P. Holland[1]*

[1]University of Zurich, Department of Chemistry, Winterthurerstrasse 190, CH-8057, Zurich, Switzerland

[2] Klinik für Kleintierchirurgie, Vetsuisse-Fakultät, University of Zurich, Winterthurerstrasse 260, CH-8057, Zurich, Switzerland

* Corresponding Author:

Prof. Dr Jason P. Holland

Tel: +41.44.63.53.990

E-mail: jason.holland@chem.uzh.ch

Website: www.hollandlab.org

First Author:

Jonas Genz

ORCID: orcid.org/0000-0002-3477-3648

E-mail: jonas.genz@chem.uzh.ch

Table of Contents

Methods ... *6*

Chemicals and solvents ... 6

NMR spectroscopy ... 6

Mass spectrometry ... 6

Electronic absorption (UV/vis) spectroscopy: ... 6

High-performance liquid chromatography ... 7

Flash chromatography ... 7

Size-exclusion chromatography (SEC) ... 7

Radioactivity ... 7

Quantification of radioactivity ... 8

Photochemistry ... 9

Cell culture ... 9

Cell binding ... 9

Animals and xenograft models ... 10

Planar γ-ray scintigraphy imaging ... 10

Biodistribution studies (*ex vivo*) ... 11

Effective half-life measurements ... 11

Statistical analysis ... 11

Radiosynthesis of $[^{99m}Tc(H_2O)_3(CO)_3]^+$ ... 11

Scheme S1. Radiosynthesis of $[^{99m}Tc(H_2O)_3(CO)_3]^+$. ... 11

Figure S1. Radio-HPLC chromatograms for $[^{99m}Tc(H_2O)_3(CO)_3]^+$ and $[^{99m}TcO_4]^-$ ... 12

Radiosynthesis of $[^{188}Re(HPO_4)(H_2O)_2(CO)_3]^-$ ... 13

Scheme S2. Radiosynthesis of $[^{188}Re(HPO_4)(H_2O)_2(CO)_3]^-$ ... 13

Figure S2. Radio-HPLC chromatograms for $[^{188}Re(HPO_4)(H_2O)_2(CO)_3]^-$ and $[^{188}ReO_4]^-$. ... 13

Figure S3. HR-MS (ESI-) spectrum of $[^{188}ReO_4]^-$ and $[^{nat}Re(HPO_4)(H_2O)_2(CO)_3]^-$ (Marked with arrow). ... 14

Figure S4. $^{31}P\{^1H\}$-NMR of the reactions of $[^{nat}Re(H_2O)_3(CO)_3]^+$ with phosphate. ... 15

Radiosynthesis of $[^{188}Re(H_2O)_3(CO)_3]^+$ ... 19

Scheme S3. Radiosynthesis of $[^{188}Re(H_2O)_3(CO)_3]^+$. ... 19

Figure S7. Radio-HPLC chromatograms for $[^{188}Re(H_2O)_3(CO)_3]^+$ and $[^{188}ReO_4]^-$. ... 19

Synthesis and Characterization ... *20*

Scheme S4. Overall sequence for the synthesis of photoactivatable ligand L1. ... 20

Scheme S5. Synthesis of compound 7 ... 20

Compound 7 ... 20

Figure S8. ^1H (400 MHz, DMSO-*d6*, 298 K) NMR spectrum of compound 7. ... 21

Figure S9. $^{13}C\{^1H\}$ (101 MHz, DMSO-d_6, 298 K) NMR spectrum of compound 7. ... 21

Figure S10. $^{13}C\{^1H\}$-DEPT-135 (101 MHz, DMSO-d_6, 298 K) NMR spectrum of compound 7. ... 21

Figure S11. HRMS (ESI+) spectrum of compound 7. ... 21

Scheme S6. Synthesis of compound 2. ... 22

Figure S12. ^1H (400 MHz, $CDCl_3$, 298 K) NMR spectrum of compound 2. ... 22

Figure S13. $^{13}C\{^1H\}$ (101 MHz, $CDCl_3$, 298 K) NMR spectrum of compound 2. 23
Figure S14. $^{13}C\{^1H\}$-DEPT-135 (101 MHz, $CDCl_3$, 298 K) NMR spectrum of compound 2. 23
Figure S15. HRMS (ESI+) spectrum of compound 2. 23
Scheme S7. Synthesis of compound 3. 24
Compound 3 24
Figure S16. ^1H (400 MHz, $CDCl_3$, 298 K) NMR spectrum of compound 3. 24
Figure S17. $^{13}C\{^1H\}$ (101 MHz, $CDCl_3$, 298 K) NMR spectrum of compound 3. 24
Figure S18. $^{13}C\{^1H\}$-DEPT-135 (101 MHz, $CDCl_3$, 298 K) NMR spectrum of compound 3. 25
Figure S19. HRMS (ESI+) spectrum of compound 3. 25
Scheme S8. Synthesis of compound 4. 26
Compound 4 26
Figure S20. ^1H (400 MHz, D_2O, 298 K) NMR spectrum of compound 4. 26
Figure S21. $^{13}C\{^1H\}$ (101 MHz, D_2O, 298 K) NMR spectrum of compound 4. 26
Figure S22. $^{13}C\{^1H\}$-DEPT-135 (101 MHz, D_2O, 298 K) NMR spectrum of compound 4. 27
Figure S23. HRMS (ESI+) spectrum of compound 4. 27
Scheme S9. Synthesis of compound 5. 27
Compound 5 27
Figure S24. ^1H (400 MHz, $CDCl_3$, 298 K) NMR spectrum of compound 5. 28
Figure S25. $^{13}C\{^1H\}$ (101 MHz, CD_3OD, 298 K) NMR spectrum of compound 5. 28
Figure S26. $^{13}C\{^1H\}$-DEPT-135 (101 MHz, CD_3OD, 298 K) NMR spectrum of compound 5. 28
Figure S27. HRMS (ESI+) spectrum of compound 5. 29
Scheme S10. Synthesis of compound L1. 29
Compound L1 29
Figure S28. ^1H (400 MHz, D_2O, 298 K) NMR spectrum of compound L1. 30
Figure S29. $^{13}C\{^1H\}$ (101 MHz, D_2O, 298 K) NMR spectrum of compound L1. 30
Figure S30. $^{13}C\{^1H\}$-DEPT-135 (101 MHz, D_2O, 298 K) NMR spectrum of compound L1. 30
Figure S31. HRMS (ESI+) spectrum of compound L1. 31
Figure S32. Reverse-phase analytical HPLC chromatogram of ligand L1, λ = 254 nm. 31
Scheme S11. Synthesis of $^{nat}Re\text{-}L1^+$. 31
$^{nat}Re\text{-}L1^+$ complex 31
Figure S33. ^1H (400 MHz, CD_3OD, 298 K) NMR spectrum of $^{nat}Re\text{-}L1^+$. 32
Figure S34. $^{13}C\{^1H\}$ (400 MHz, CD_3OD, 298 K) NMR spectrum of $^{nat}Re\text{-}L1^+$. 32
Figure S35. $^{13}C\{^1H\}$-DEPT-135 (101 MHz, CD_3OD, 298 K) NMR spectrum of $^{nat}Re\text{-}L1^+$. 32
Figure S37. HRMS (ESI+) spectrum of $^{nat}Re\text{-}L1^+$. 33
Radiosynthesis of $[^{99m}Tc(CO)_3L1]^+$ ($^{99m}Tc\text{-}L1^+$) 33
Scheme S12. Radiosynthesis of $^{99m}Tc\text{-}L1^+$. 33
Figure S38. Radio-HPLC chromatograms for $[^{99m}Tc(H_2O)_3(CO)_3]^+$, $^{99m}Tc\text{-}L1^+$ and $^{nat}Re\text{-}L1^+$. 34
Radiosynthesis of $[^{188}Re(CO)_3L1]^+$ ($^{188}Re\text{-}L1^+$) 34
Scheme S13. Radiosynthesis of $^{188}Re\text{-}L1^+$ 34
Figure S39. Radio-HPLC chromatograms for $^{188}Re\text{-}L1^+$ and the precursor. 35

Figure S42. Stability test of ^{188}Re-L1^{+} in PBS and challenge with 1 mM His/Cys 36

Scheme S14. Synthesis of [^{nat}Re(CO)$_3$(diethylenetriamine)]Br ([^{nat}Re(CO)$_3$(DETA)]Br) 36

Complex [^{nat}Re(CO)$_3$(diethylentriamine)]Br 36

Figure S43. HPLC chromatogram of compound [^{nat}Re(CO)$_3$(diethylenetriamine)]$^{+}$. 37

Figure S44. HRMS (ESI+) spectrum of [^{nat}Re(CO)$_3$(diethylenetriamine)]$^{+}$ 37

Electronic Absorption (UV-VIS) Spectroscopy: 37

Figure S45. Electronic absorption spectra of ligand L1 (green), the non-photoactive complex ^{nat}Re-diethylenetriamine (black) and the complex ^{nat}Re-L1^{+} (blue) (0.1 mM, 30 °C, MeOH) 37

Figure S46. Electronic absorption spectra of a dilution series of ligand L1 (30 °C, MeOH) 38

Figure S47. Electron absorption spectra of a dilution series of the complex ^{nat}Re-L1^{+} (30 °C, MeOH). 38

Radiosynthesis of [^{188}Re(CO)$_3$(diethylenetriamine)]$^{+}$ 39

Scheme S15. Radiosynthesis of [^{188}Re(CO)$_3$(diethylenetriamine)]$^{+}$ 39

Figure S51. Radio-HPLC chromatograms for [^{188}Re(HPO$_4$)(H$_2$O)$_2$(CO)$_3$]$^{-}$, [^{188}Re(CO)$_3$(diethylenetriamine)]$^{+}$ and [^{nat}Re(CO)$_3$(diethylenetriamine)]$^{+}$. 40

Photoradiosynthesis of [^{188}Re(CO)$_3$L1-azepin]-HSA (^{188}Re-HSA) 41

Scheme S16. Radiosynthesis of ^{188}Re-HSA. 41

Figure S54. Radio-iTLC chromatogram of ^{188}Re-HSA. 41

Figure S55. Preparative PD-10 SEC profile for the purification of ^{188}Re-HSA. 41

Figure S56. Preparative PD-10 SEC profile with the full elution of the crude ^{188}Re-HSA. 42

Figure S57. Size-exclusion (HPLC) chromatogram of ^{188}Re-HSA. 42

Figure S58. Preparative PD-10 SEC profile of the dark control reaction of the complex ^{188}Re-L1^{+}, ^{188}Re-L1^{+} with HSA (dark control), and ^{188}Re-HSA after photolysis. 42

Figure S59. Preparative PD-10 SEC profile of ^{188}Re-L1^{+} and the photolyzed complex ^{188}Re-L1-azepin-2-ol. 42

Figure S60. Preparative PD-10 SEC profile of [^{99m}TcO$_4$]$^{-}$ and [^{188}ReO$_4$]$^{-}$. 43

Figure S61. Stability test of ^{188}Re-HSA in (A) PBS, and (B) analysis of the small molecule fraction after 24 h by radio-HPLC. 43

Photograph S1. Reaction setup of the photoradiolabeling reaction. 43

Photoradiosynthesis of [^{99m}Tc(CO)$_3$L1]-azepin-onartuzumab (^{99m}Tc-onartuzumab). 44

Scheme S15. Radiosynthesis of ^{99m}Tc-onartuzumab. 44

Figure S62. iTLC chromatogram of ^{99m}Tc-onartuzumab. 44

Figure S63. Preparative PD-10 SEC profile for the purification of ^{99m}Tc-onartuzumab. 45

Figure S64. Size-exclusion chromatogram of ^{99m}Tc-onartuzumab. 45

Scheme S16. Radiosynthesis of ^{188}Re-onartuzumab. 45

Figure S65. iTLC chromatogram of ^{188}Re-onartuzumab. 46

Figure S66. Preparative PD-10 SEC profile for the purification of ^{188}Re-onartuzumab. 46

Figure S67. Size-exclusion chromatogram of ^{188}Re-onartuzumab. 46

Figure S68. Stability test of ^{99m}Tc-onartuzumab in (A) PBS, and (B) analysis of the small molecule fraction after 24 h by radio-HPLC. 47

Figure S69. Stability test of ^{188}Re-onartuzumab in PBS. 47

Figure S71. Cellular binding assay of ^{188}Re-onartuzumab on 15 × 10^{6} MKN-45 cells. 47

Table S1. Doses for the animal experiments. 48

Planar γ-scintigraph images 49

Figure S72. Maximum intensity projection (MIP) ^{188}Re-onartuzumab planar □-scintigraph images of a representative mouse. T = tumor. 49

Figure S73. Maximum intensity projection (MIP) ^{99m}Tc-onartuzumab planar □-scintigraph images of a representative mouse. T = tumor. 49

Figure S74. Comparison of the biodistribution results expressed as tissue uptake (%ID/g). 50

Figure S75. Comparison of the biodistribution results expressed as tissue uptake (SUV). 50

Figure S76. Comparison of the tumor-to-tissue contrast ratio based on values reported in %ID g^{-1}. 50

Table S2. *Ex vivo* biodistribution data measured for ^{99m}Tc- and ^{188}Re-onartuzumab in the normal groups. 51

Table S3. *Ex vivo* biodistribution data measured for ^{99m}Tc- and ^{188}Re-onartuzumab in the blocking groups...52

Effective and biological half-life measurements 53

Figure S77. One-phase model (Prism 10, 'One-phase' model) for the experimentally measured effective half-life of (A) ^{188}Re-onartuzumab (0 h to 22 h, *n* = 4), and (B) ^{188}Re-onartuzumab (17 h to 72 h, *n* = 4). 53

Figure S78. One-phase model for the experimentally measured effective half-life of ^{99m}Tc-onartuzumab (*n* = 4) 53

Methods

Chemicals and solvents

Unless otherwise stated, all other chemicals were of reagent grade and purchased from Sigma Aldrich (St. Louis, MO), Merck (Darmstadt, Germany), abcr (Karlsruhe, Germany), or CheMatech (Dijon, France). Water (>18.2 MΩ·cm at 25 °C, Milli-Q® Direct 8/16 System, Millipore, Molsheim, France). Solvents for reactions were of reagent grade and where necessary, were dried over molecular sieves. Solvent evaporation was performed under reduced pressure by using a rotary evaporator (Rotavapor R-300, Büchi Labortechnik AG, Flawil, Switzerland).

NMR spectroscopy

^{1}H, $^{13}C\{^{1}H\}$ and $^{31}P\{^{1}H\}$ NMR spectra were measured in deuterated solvents on a Bruker AV-400 (^{1}H: 400 MHz, ^{13}C: 100.6 MHz, ^{31}P: 162 MHz) or a Bruker AV-500 (^{1}H: 500 MHz, ^{13}C: 125.8 MHz, ^{31}P: 202 MHz) spectrometer. Chemical shifts (δ) are expressed in parts per million (ppm) relative to the resonance of the residual solvent peaks, for example, with DMSO δ_H=2.50 ppm and δ_C=39.5 ppm with respect to tetramethylsilane (TMS, δ_H and δ_C = 0.00 ppm). Coupling constants (*J*) are reported in Hz. Peak multiplicities are abbreviated as follows: *s* (singlet), *d* (doublet), *dd* (doublet of doublets), *t* (triplet), *q* (quartet), *m* (multiplet), and *b* (broadened). Two-dimensional NMR experiments including ^{1}H-^{1}H correlation spectroscopy (COSY), ^{13}C-^{1}H heteronuclear single quantum coherence (HSQC) and ^{13}C-^{1}H heteronuclear multiple bond correlation (HMBC) were performed to aid the assignment of the ^{1}H and ^{13}C spectra.

Mass spectrometry

High-resolution electrospray ionization mass spectra, HRMS (ESI) were measured by the mass spectrometry service at the Department of Chemistry, University of Zurich.

Electronic absorption (UV/vis) spectroscopy:

UV/vis spectra were acquired on a *SPECORD® 250* (Analytic Jena, Überlingen, Germany) a Hellma quartz cell for spectroscopy with optical path length of 1.0 cm.

FT-IR

FT-IR spectra were recorded on a *Spectrum Two™ FT-IR Spectrometer* (Perkin Elmmer, Shelton, USA).

b: broad, *w*: weak, *s*: strong

High-performance liquid chromatography

Analytical high-performance liquid chromatography (HPLC) experiments were performed by using Hitachi Chromaster HPLC systems fitted with a reverse EC 250/3 NUCLEOSIL C18 PAH (3 mm ID × 250 mm, 5µm, Macherey-Nagel, Düren, Germany) column. This system was also fitted to a $FlowStar^2$ LB 514 radioactivity detector (Berthold Technologies, Zug, Switzerland) equipped with a 30 µL g cell (BGO-X, Berthold Technologies) for analyzing radiochemical reactions. For all HPLC chromatograms shown, solvent A = 18.2 MΩ·cm H_2O+0.1% TFA and solvent B = MeOH and the method used a flow-rate of 0.5 mL min^{-1} with a step gradient of A: t = 0 min 10% B; t = 3 min 25% B followed by a linear gradient t = 7 min 25% B, t = 10 min 100% B, t = 16 min 10% B, t = 17 min 10% B.

Flash chromatography

Flash chromatography was performed on a Biotage® Isolera™ One (Normal phase) or a Biotage® Selekt Enkel (Reverse phase) system by Biotage (Uppsala, Sweden). RediSep® Flash Coloumns by Teledyne LABS (Thousand Oaks, California, USA) were used. Solvents for normal phase were technical grade and for reverse phase of HPLC grade.

Size-exclusion chromatography (SEC)

Two SEC methods were used. The first method used a size-exclusion column (BioRad Laboratories, ENrich SEC 70, 10±2 µm, 10 mm ID × 300 mm) connected to an automated HPLC device (Rigol L-3000, Contrec AG, Switzerland) equipped with a DAD UV-Vis detector (absorbance measured at 280 nm and/or 430 nm) and an in-line radioactivity detector ($FlowStar^2$ LB 514, Berthold Technologies, Zug, Switzerland). Isocratic elution with phosphate buffered saline (PBS, pH7.4, 1x) was used for all SEC-HPLC analyses (flow-rate = 1 mL min^{-1}). The second method used manual SEC employing a PD-10 desalting column (Sephadex G-25 resin, 85–260 µm, 14.5 mm ID × 50 mm, >30 kDa, GE Healthcare). For analytical procedures, manual PD-10 columns were eluted with sterile PBS. A total of 50×200 µL up to 110x200 mL fractions were collected to a final elution volume of 10 mL up to 22 mL. Manual SEC columns were also used for preparative purification and reformulation of radiolabeled protein samples (sterile PBS, pH7.4).

Radioactivity

Caution: Rhenium-188 ($t_{1/2}$ = 17.001 h, $E_{max}(\beta^-)$ = 2120 keV, $E_{av}(\beta^-)$ = 795 keV, $E\gamma$ = 155.0 keV [I = 15.5%]) emits electrons and low-energy gamma rays. Technetium-99m ($t_{1/2}$ = 6.007 h, $E\gamma$ = 140.5 keV [I = 89%]) emits low-energy gamma rays. All operations must be performed by qualified personnel in an approved facility and following safety guidelines set forth by the local authorities, and the Nuclear Regulatory Commission. Experimental manipulations should first be practiced with non-radioactive samples and researchers should follow the ALARA (As Low As Reasonable Achievable) protocols to minimize exposure to ionizing radiation.

Rhenium-188

Rhenium-188 (^{188}Re) as $Na[^{188}ReO_4]$ was obtained from an alumina-based $^{188}W/^{188}Re$ generator. Tungsten-188 (^{188}W) was produced by neutron irradiation in high flux nuclear reactors. It was fixed into an aluminium oxide (Al_2O_3) column in quartz glass. ^{188}Re, which is formed by b$^-$ decay from ^{188}W ($t_{1/2}$ = 69.78 d, $E_{max}(\beta^-)$ = 349 keV) was obtained from the column by elution with 10 to 20 mL of aqueous 0.9% sodium chloride solution. If necessary, the eluate was concentrated by rotary evaporation. The generator was supplied by OncoBeta GmbH, Garching, Germany. The solution was passed over a Light Alumina N SepPak® cartridge (Waters, Milford, Massachusetts, USA), pre-conditioned with 5 mL EtOH and 10 mL saline solution, to remove potential breakthrough of $Na_2[^{188}WO_4]$.

^{188}Re-labelling reactions were performed under an N_2 atmosphere, at the stated temperature and pH. Radioactive reactions were monitored by using radio-HPLC with the gradient as stated above and by using instant thin-layer chromatography (iTLC). Glass-fiber TLC plates (iTLC-SG, Agilent Technologies) were developed by using acetone as the eluent and were analyzed on a radio-TLC detector (SCAN-RAM, LabLogic Systems Ltd, Sheffield, United Kingdom). Radiochemical conversion yields (RCC) were determined by integrating the data obtained by the radio-HPLC and determining both the percentage of radiolabeled product ($[^{188}ReO_4]^-$ (R_t = 3.25 min), $[^{188}Re(HPO_4)(H_2O)_2(CO)_3]^-$ (R_t = 4.00 min) and $^{188}Re\text{-}L1^+$ (R_t = 14.85 min). ^{188}Re-labeled protein samples were characterized by manual SEC and by automated SEC-HPLC methods.

Technetium-99m

Technetium-99m (^{99m}Tc) as $Na[^{99m}TcO_4]$ was obtained from an alumina-based $^{99}Mo/^{99m}Tc$ generator (Ultra-Techneknow™ FM). Molybdenum-99 (^{99}Mo) was produced by neutron irradiation in high flux nuclear reactors. It was fixed into an aluminum oxide (Al_2O_3) column in quartz glass. ^{99m}Tc, which is formed by β^- decay from ^{99}Mo ($t_{1/2}$ = 65.92 h, $E_{max}(\beta^-)$ = 1357.8 keV) was obtained from the column by elution with between 4 and 10 mL of aqueous 0.9% sodium chloride solution. The generator was supplied by Curium™ (Berlin, Germany).

^{99m}Tc-labeling reactions were performed under an N_2 atmosphere, at the stated temperature and pH. Radioactive reactions were monitored by using radio-HPLC with the gradient elution method as stated above, and by iTLC. Glass-fiber TLC plates were developed by using acetone as the eluent and were analyzed on a radio-TLC detector. RCC was determined by integrating the data obtained by the radio-HPLC and determining both the percentage of radiolabeled product ($[^{99m}TcO_4]^-$ (R_t = 3.25 min), $[^{99m}Tc(H_2O)_3(CO)_3]^+$ (R_t = 6.15 min) and $^{99m}Tc\text{-}L1^+$ (R_t = 14.55 min)). ^{99m}Tc-labeled protein samples were characterized by manual SEC and by automated SEC-HPLC methods.

Quantification of radioactivity

Fractions obtained from manual SEC and tissues collected from the animal experiments were measured on a gamma counter (HIDEX Automatic Gamma Counter, Hidex AMG, Turku, Finland) by using a counting time of 30 s, and an energy window between 15–250 keV for ^{99m}Tc (141 keV emission) and between 15–250 keV for ^{188}Re (155 keV emission). Appropriate background and decay corrections were applied throughout.

Activity measurements were performed by using a dose calibrator (ISOMED 2010 Activimeter, Nuklear-Medizintechnik Dresden GmbH, Germany).

The radiochemical purity (RCP) of labeled protein samples was determined by automated SEC-HPLC (see above) and manual SEC.

Photochemistry

Photochemical conjugation experiments were performed in transparent glass vials using an ultra-violet light-emitting diode (LED; 395 nm). The LED intensity was adjusted by using a digital UV-LED controller (Opsytec Dr. Gröbel GmbH, Ettlingen, Germany), where 100% corresponded to a power of approximately 355 mW at 395 nm. LED intensity was measured by using a S470C Thermal Power Sensor Head Volume Absorber, 0.25–10.6 µm, 0.1 mW–5W, Ø15 mm. The LED (395 nm) had a maximum emission intensity at 389.9 nm (FWHM = 9.1 nm). Photochemical reactions were stirred gently (stirring rate 50 rpm) to avoid potential mechanical damage to the protein. The temperature of all photochemical conjugation reactions was 23 ± 2 ºC.

Cell culture

The human gastric cancer cell line MKN-45 was obtained from the Leibniz Institute DSMZ-German collection of Microorganisms and Cell Cultures (ACC 409). Cells were cultured at 37 °C in a humidified 5% CO_2 atmosphere in RPMI-1640 media (without phenol-red) supplemented with fetal bovine serum (FBS, 10% *v/v*, ThermoFisher Scientific) and penicillin/streptomycin (P/S, 1% *v/v* of penicillin 10000 U/mL and streptomycin 10 mg mL^{-1}). Cells were grown by serial passage and were harvested by using trypsin-EDTA solution (0.025%) and subsequent centrifugation at 100 G for 5 minutes.

Cell binding

Cells were harvested and one concentration of 1:2 dilution (in triplicate or quadruplicate) was prepared in appropriate cell media. The purified radiotracer (~ 5 ng, 100 µL PBS) was added to the cell suspension. To determine the extent of non-specific binding a ~35,000 fold excess of non-radiolabeled sample (3 µL, 60 mg/mL, 0.18 mg) was mixed with stated quantity of the radiolabeled antibody. Cells were shaken at 800 rpm at 37 °C in a thermomixer. The samples were incubated for 4 h. The cells were pelleted by centrifugation (2000 rpm, 4 °C, 4 min), the media was discarded, the pellet was washed twice with ice-cold PBS (2 × 1 mL), centrifuged again by discarding the supernatant, and removal of PBS between each wash. Cell-associated radioactivity of the washed pellet and the washed solution was measured in the gamma counter. The cell binding was determined as the ratio between the bound radioactivity to the cells and the unbound radioactivity in the combined supernatant solutions deriving from the wash steps.

Antibody samples

Onartuzumab (MW = 99.16 kDa; 66 Lys; molar absorption coefficient, ε_{280} = 161,465 M^{-1} cm^{-1}) was supplied by Genentech/Roche as the formulated drug (MetMAb). Stock solutions of MetMAb contained at a protein concentration of 60 mg mL^{-1} in a formulation buffer comprising 10 mmol L^{-1} histidine succinate, 106 mmol L^{-1} trehalose dihydrate, and 0.02% polysorbate 20 at pH5.7. Protein samples were aliquoted and stored at -80 ºC prior to use.[1]

Animals and xenograft models

All experiments involving mice were conducted in accordance with an animal experimentation license approved by the Zurich Canton Veterinary Office, Switzerland (Jason P. Holland). Experimental procedures were planned and conducted in accordance with the ARRIVE 2.0 guidelines. Female athymic nude mice (Crl:NU(NCr)-*Foxn1*^{nu}, 20–31 g, 6–8 weeks old) were obtained from Charles River Laboratories Inc. (Freiburg im Breisgau, Germany), and were allowed to acclimatize at the University of Zurich Laboratory Animal Services Centre vivarium for 1 week prior to experimentation. Mice were provided with food and water *ad libitum*. Tumors were induced on the right shoulder or flank by sub-cutaneous (s.c.) injection of 3.0×10^6 (^{99m}Tc and ^{188}Re, 24 h time point) or 7.0×10^6 (^{188}Re, 72 h time point) MKN-45 cells. The cells were injected in a 200 µL suspension of a 1:1 v/v mixture of sterile PBS (pH7.4) and reconstituted basement membrane (Corning® Matrigel® Basement Membrane Matrix, obtained from VWR International). Palpable tumors developed after a period of 7 days (^{99m}Tc and ^{188}Re, 24 h time point) or 12 days (^{188}Re, 72 h time point) and the average volume of the MKN-45 tumors was 270 ± 95 mm^3 (n = 8 mice, ^{188}Re, 24 h time point), 876 ± 491 mm^3 (n = 10 mice, ^{188}Re, 72 h time point) and 645 ± 391 mm^3 (n = 8 mice, ^{99m}Tc, 24 h time point). Mice were randomized before the study.

Planar γ-ray scintigraphy imaging

Planar γ-ray scintigraphy imaging experiments were conducted on a γ-Eye (BIOEMTECH, Athens, Greece). Approximately 3 minutes prior to recording each γ-ray image, mice were anesthetized by inhalation of between 2–4% isoflurane (Attane^{TM}, Piramal Critical Care, Inc, Bethlehem, PA, USA)/oxygen gas mixture and placed on the scanner bed in the prone position. γ-ray images were recorded at various time points between 1–24 h post-administration of the radiotracer. Anesthesia was maintained by an experienced animal experimenter by controlling the isoflurane dose between 1.5–2.0% and monitoring respiration rate throughout the image acquisition. List-mode data were acquired for 10 min to 60 min for live-animal imaging and 12 h for *ex vivo* imaging by using a γ-ray energy window of 140.51 keV (±20%) for ^{99m}Tc and 155.04 keV (±20%) for ^{188}Re. Detector signals were used to localize an event by employing the Anger logic.[2,3] The reported reconstructed spatial resolution is 1.9 mm and energy resolution of ~19%. System spatial resolution was measured with a ^{99m}Tc capillary-source (40 mm long and a 1.1 mm inner diameter) placed at multiple distances (0 to 10 mm) from the detector above the collimator. The Full Width at Half Maximum (FWHM) of the source profile was calculated by using a Gaussian fit. FWHM value (in mm) was calculated by multiplying the variance of the distribution σ with (2.35 + pixel size [1.45 mm × 1.45 mm]). Position mapping and uniformity correction are common practices for pixelated scintillator-based small field-of-view scintigraph systems. As a part of the gamma-ray detector, PSPMTs often behave in a non-uniform manner. The correction methods involve position mapping, uniformity correction,

and energy correction. Position mapping was realized by placing a 3.7 MBq ^{99m}Tc point source at 1 m above the scintillator without the collimator. Using the obtained raw flood image, a grid that maps each crystal pixel is determined. The counts in each crystal pixel are summed leading to the flood matrix, which corrects the intensity of the raw images. An energy window of ±20 % is applied in each crystal pixel for energy correction. Attenuation correction is not applied. Physical decay to time of injection is applied. The γ-Eye detector is linear for high count rates and activities up to 148 MBq, and therefore, dead-time count losses are not considered. The selection of detector materials and electronics allows the system to work properly for high activities.

Biodistribution studies (*ex vivo*)

Biodistribution studies were conducted after the final imaging time point to evaluate the radiotracer uptake in tumor-bearing mice. Animals (n = 4 or 5 mice per group) were anesthetized individually by isoflurane and euthanized by isoflurane overdose followed by terminal cardiac puncture and exsanguination. A total of 15 tissues (including the tumor) were removed, rinsed in water, dried in air for approx. 2 min, weighed and counted on a calibrated gamma counter for accumulation of activity. The mass of radiotracer formulation injected into each animal was measured and used to determine the total number of counts per minute (cpm) injected into each mouse by comparison to a standard syringe of known activity and mass. Count data were background- and decay-corrected, and the tissue uptake for each sample (determined in units of percentage injected dose per gram [%ID g^{-1}]) was calculated by normalization to the total amount of activity injected for each individual animal.

Effective half-life measurements

The effective half-life $t_{1/2}$(eff) / h was calculated from the measurement of total radioactivity present in the animal over time by using a dose calibrator.

Statistical analysis

Where appropriate, data were analyzed by the unpaired, two-tailed Student's t-test. Differences at the 95% confidence level, (*) P-value <0.05, were considered statistically significant. Note: (**) P-value <0.01; (***) P-value <0.001, (****) P-value <0.0001.

Radiosynthesis of $[^{99m}Tc(H_2O)_3(CO)_3]^+$

Scheme S1. Radiosynthesis of $[^{99m}Tc(H_2O)_3(CO)_3]^+$.

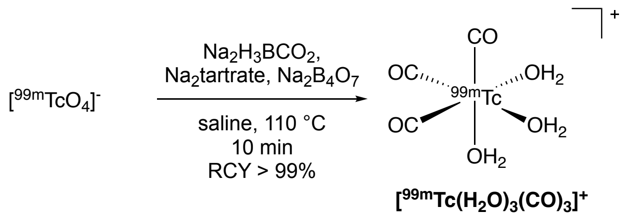

Radiolabeling reactions to prepare $[^{99m}Tc(H_2O)_3(CO)_3]^+$ were accomplished by the addition of 1 mL of $[^{99m}TcO_4]^-$ stock solution to a N_2 flushed vial containing 4 mg $Na_2(H_3BCO_2)$, 7 mg Na_2tatrate dihydrate and 7 mg $Na_2B_4O_7$-10-hydrate with a total reaction volume of 1.00 mL at pH>12. Test reactions were performed with 10 to 50 MBq and reactions for the animal doses with up to 1.648 GBq of $[^{99m}TcO_4]^-$. The reactions were heated to 110 °C with a microwave (Biotage® Initiator) for 10 min. The reactions were monitored by analytical radio-HPLC giving a radiochemical yield (RCY) >99% and a radiochemical purity of (RCP) >97% (R_t = 6.26 min). The total reaction time was 10 min and the product was used immediately without further purification. The product was characterized by analytical HPLC following the method described in the general section.

Figure S1. Radio-HPLC chromatograms for $[^{99m}Tc(H_2O)_3(CO)_3]^+$ and $[^{99m}TcO_4]^-$.

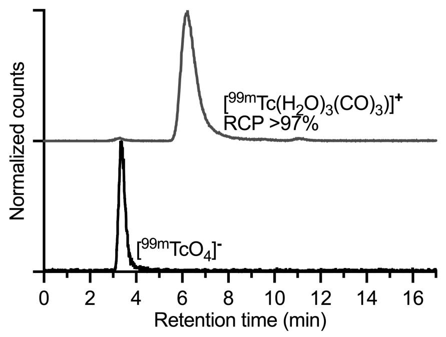

Radiosynthesis of $[^{188}Re(HPO_4)(H_2O)_2(CO)_3]^-$

Scheme S2. Radiosynthesis of $[^{188}Re(HPO_4)(H_2O)_2(CO)_3]^-$.

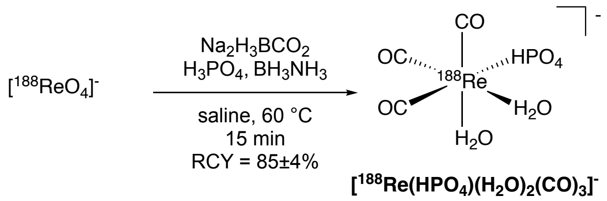

Radiolabeling reactions to prepare $[^{188}Re(HPO_4)(H_2O)_2(CO)_3]^-$ were accomplished by the addition of 1 mL of $[^{188}ReO_4]^-$ stock solution containing 7 mL of 85% H_3PO_4 (104 mmol) to a N_2 flushed vial containing 5 mg BH_3NH_3 and 5 mg $Na_2(H_3BCO_2)$ with a total reaction volume of 1.00 mL. Test reactions were performed with 10 to 50 MBq and reactions for the animal doses with up to 688 MBq of $[^{188}ReO_4]^-$. The reactions were heated to 60 °C for 15 min. The reactions were monitored by analytical radio-HPLC, giving a RCC >90% and an RCY of 85±4% (*n* = 5) and RCP of >95% (R_t = 4.00 min). The product was characterized by analytical HPLC following the method described in the general section. The pH was adjusted by addition of 1 M NaOH for following reactions. The total reaction time including the pH adjustment was 20 min.

Figure S2. Radio-HPLC chromatograms for $[^{188}Re(HPO_4)(H_2O)_2(CO)_3]^-$ and $[^{188}ReO_4]^-$.

Note: The peak marked with the * is $[^{188}Re(H_2O)_3(CO)_3]^+$.

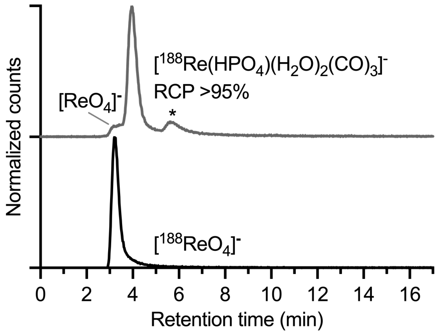

Figure S3. HR-MS (ESI-) spectrum of $[^{nat}ReO_4]^-$ and $[^{nat}Re(HPO_4)(H_2O)_2(CO)_3]^-$ (Marked with arrow).

Note: Two aqua ligands presumably dissociate during the ionization.

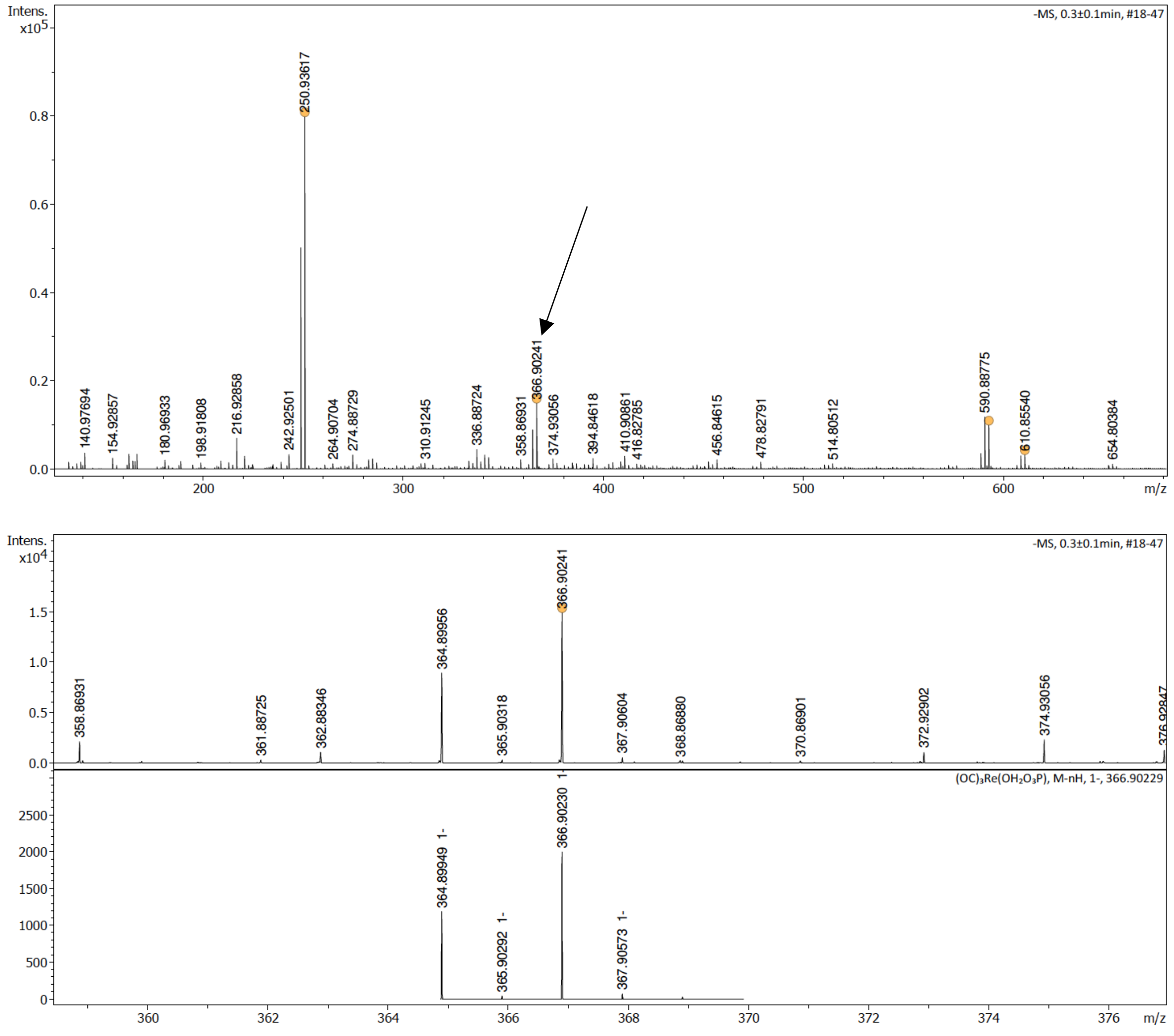

Meas. m/z	#	Ion Formula	Score	m/z	err [mDa]	err [ppm]	mSigma	rdb (neutral)	e^- Conf
250.93617	1	O_4Re	100	250.93596	-0.21	-0.82	19.7	3.0	even
366.90241	1	C_3HO_7PRe	100.00	366.90229	-0.10	-0.28	2.8	7.0	even

Figure S4. $^{31}P\{^{1}H\}$-NMR of the reactions of $[^{nat}Re(H_2O)_3(CO)_3]^+$ with phosphate.

Note: Marked with X is free PO_4^{3-}.

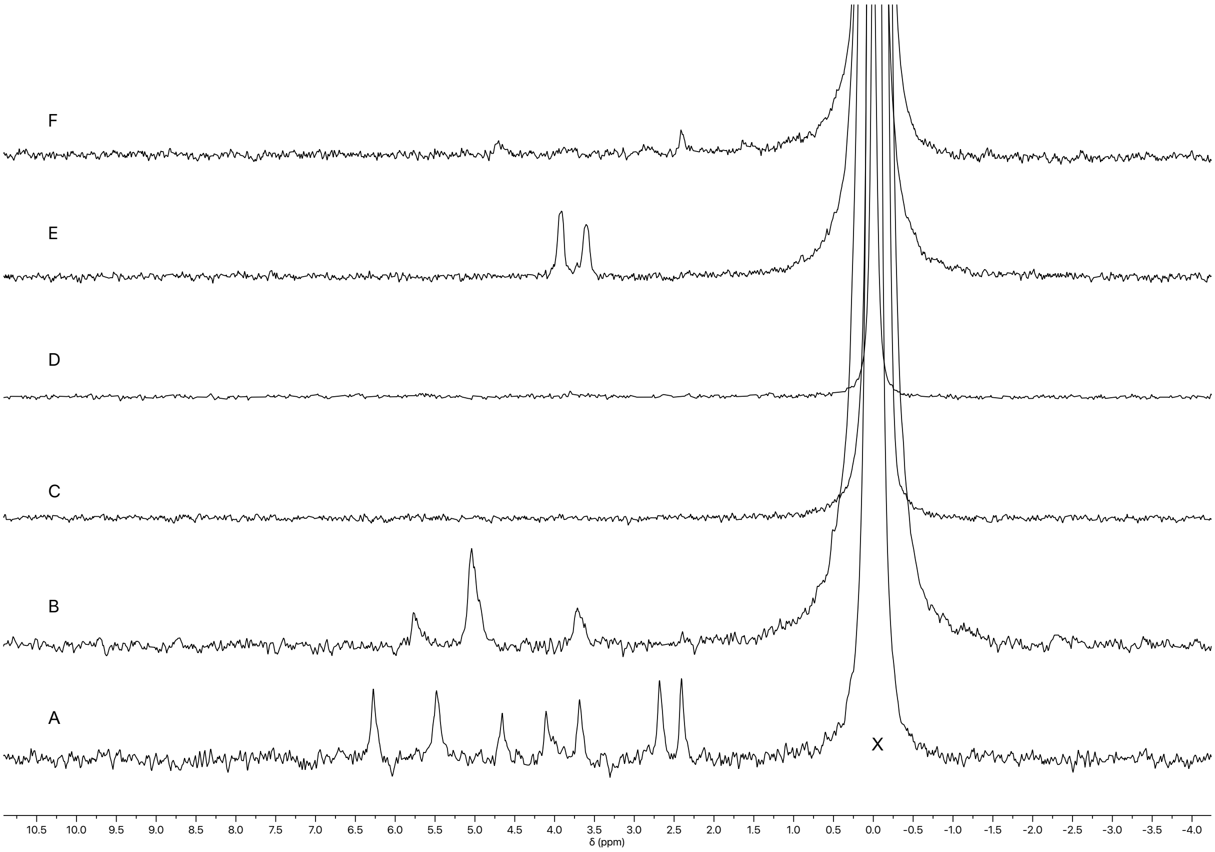

A: $[^{nat}Re(CO)_3(H_2O)_3]^+$ with 10 equiv. NaH_2PO_4 at pH6.8, 25 °C;

B: $[^{nat}Re(CO)_3(H_2O)_3]^+$ with 200 equiv. NaH_2PO_4 at pH6.8, 25 °C.;

C: $[^{nat}Re(CO)_3(H_2O)_3]^+$ with 200 equiv. NaH_2PO_4 at pH1, 25 °C.;

D: $[^{nat}Re(CO)_3(H_2O)_3]^+$ with 10 equiv. NaH_2PO_4 at pH6.8, 60 °C.;

E: $[^{nat}Re(CO)_3(H_2O)_3]^+$ with 200 equiv. NaH_2PO_4 at pH4.4.;

F: $[^{nat}Re(CO)_3(H_2O)_3]^+$ with 200 equiv. Na_2HPO_4 at pH9.6.

Dynamic ^{31}P-NMR experiments

The following conditions were chosen to ensure the best spectral quality and to reduce the complications deriving from the speciation of $[^{nat}Re(H_2O)_3(CO)_3]^+$ in aqueous phosphate solutions as already presented in Figure S4. An NMR tube filled with 500 μL of 100 mM NaH_2PO_4 (doped with 10% D_2O) at pH6.8 and 50 mM $[^{nat}Re(H_2O)_3(CO)_3]Br$ was inserted in the NMR spectrometer and allowed to equilibrate at 298 K for 1 hour. The probe was tuned and matched to the ^{31}P resonance and carefully shimmed using the band of free phosphate found at 0 ppm as linewidth indicator. Lock stability was ensured by using the '*loopadj'* command prior to any further operation. The 90° pulse for ^{31}P was calibrated manually by using the '*paropt*' routine in Topspin. The recycle delay was chosen to be 20 s by inversion recovery experiment using '*t1ir'* pulse program. The receiver gain has been optimized prior to any experiment using the '*rga*' routine, the so-obtained value was left unmodified for the

rest of the experiment. At first, we acquired a standard ^{31}P spectrum (Figure S5) which displays two separate resonances attributed to free phosphate (0 ppm) and to bound phosphate (5.83 ppm).

Figure S5. $^{31}P\{^1H\}$-NMR of [$^{nat}Re(H_2O)_3(CO)_3$]Br (50 mM in H_2O:D_2O 90:10, 202 MHz, 298 K, 100 mM NaH_2PO_4 pH6.8).

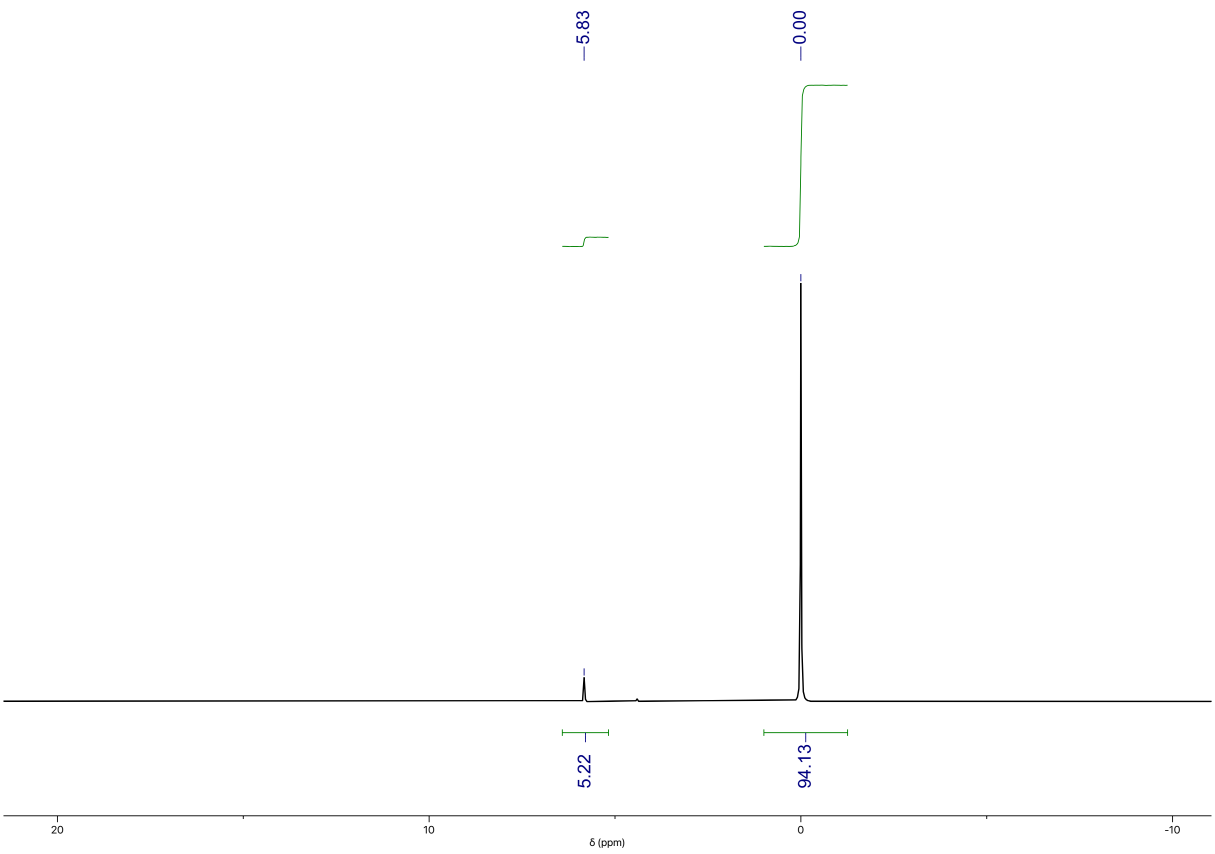

The two signals were integrated and normalized arbitrarily to 100, from the integration it is possible to calculate the molarity of free and bound phosphate which are respectively: 0.094 M and 0.0052 M. Considering a 1:1 equilibrium stoichiometry between [$^{nat}Re(H_2O)_3(CO)_3$]$^+$ and phosphate, we can write the equilibrium equations (Eq. 1 to Eq.4):

$$[\mathrm{Re(CO)_3(H_2O)_3}]^+ + \mathrm{HPO_4^{2-}} \rightleftharpoons [\mathrm{Re(H_2PO_4)(H_2O)_2(CO)_3}]^- + \mathrm{H_2O} \qquad \text{Eq. 1}$$

$$K_{eq} = \frac{[\mathrm{Re(HPO_4)(H_2O)_2(CO)_3}]^-}{[\mathrm{Re(H_2O)_3(CO)_3}]^+\,[\mathrm{HPO_4^{2-}}]} \qquad \text{Eq. 2}$$

$$C_{PO_4} = [\mathrm{HPO_4^{2-}}] + [\mathrm{Re(HPO_4)(H_2O)_2(CO)_3}]^- \qquad \text{Eq. 3}$$

$$C_{Re} = [\mathrm{Re(HPO_4)(H_2O)_2(CO)_3}]^- + [\mathrm{Re(H_2O)_3(CO)_3}]^+ \qquad \text{Eq. 4}$$

Where K_{eq} is the binding equilibrium constant, C_{PO4} is the mass balance for phosphate and C_{Re} is the mass balance for rhenium. From the mass balance of phosphate it is possible to obtain the molarity of bound phosphate, which is 0.0052 M, and the amount of [$^{nat}Re(H_2O)_3(CO)_3$]$^+$, which is 0.045 M. By taking the ratio of the concentrations

calculated (assuming 5 % of error from the NMR integrations and by propagating errors), the equilibrium constant is K_{eq}=1.24 ± 0.17. For the dynamic experiments, presaturation power was optimized manually by using the pulse program '*noediff.2*' available in the standard library of pulse programs for Bruker experiments. Note that this pulse program and the optimal parameters were kept unmodified throughout the remaining part of this experiment. The NOE-difference experiment was conducted by acquiring 32 accumulations alternating between on-resonance and off-resonance. The 16 on-resonance scans were acquired by saturating the signal at 5.83 ppm (bound phosphate) and averaged to yield the final spectra. Similarly, the off-resonance reference spectra were acquired by saturating at 20.0 ppm, a sufficiently distant and signal-free region. The experiment was repeated at different presaturation times spanning between 5 ms and 20 s. Each on-resonance spectrum, associated to a timepoint, was subject to exponential apodization (line broadening=3.0 Hz), phase-corrected, baseline corrected and subtracted from the corresponding reference (off-resonance) spectrum which was elaborated identically. The signal of free phosphate at 0 ppm resulting from the difference-spectra was integrated and the integral value plot against the presaturation time. The time-evolution of the integral of the free phosphate was fit to a first-order rate law of the form:

$$I(t) = I_{sat} - (I_{sat} - I_0)\exp(-k_{obs}t) \qquad \text{Eq. 5}$$

Figure S6. Left: Difference spectra $^{31}P\{^1H\}$-NMR of $[^{nat}Re(H_2O)_3(CO)_3]Br$ (50 mM in H_2O:D_2O 90:10, 202 MHz, 298 K, 100 mM NaH_2PO_4 pH6.8). Right: Integral values (bullets) and best fit to Eq. 5 (dashed line).

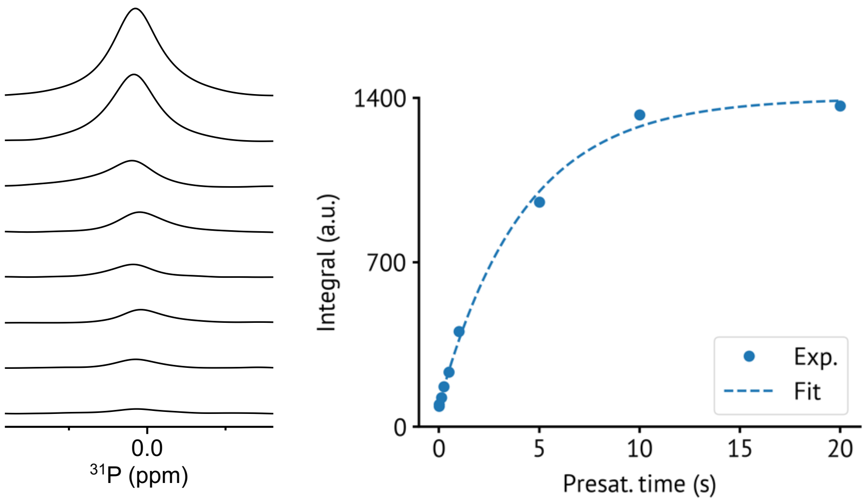

Where $I(t)$ is the observed integral as function of time, I_0, I_{sat} and k_{obs} are fitted parameters indicating respectively: the integral at no presaturation, the integral at infinite presaturation time, and rate constant of phosphate exchange. The best fit parameters are (reported as value ± standard deviation of fitting): $k_{obs} = 0.24 \pm 0.02$ M^{-1} s^{-1}, $I_0 = 93.1 \pm 1.5$, $I_{sat} = 1398 \pm 32$ with $R^2 = 0.997$. By considering that k_{obs} is the convolution of the forward (k_1) and reverse (k_{-1}) binding rate constants, it is possible to extrapolate the kinetic parameters for the equilibrium with the following equations:

$$k_{obs} = k_1 + k_{-1} \quad \text{Eq. 6}$$

$$K_{eq} = \frac{k_1}{k_{-1}} \quad \text{Eq. 7}$$

$$k_1 = \frac{K_{eq} k_{obs}}{K_{eq} + 1} \quad \text{Eq. 8}$$

$$k_{-1} = \frac{k_{obs}}{K_{eq} + 1} \quad \text{Eq. 9}$$

The calculated values are: $k_1 = 0.133 \pm 0.013$ M^{-1} s^{-1} and $k_{-1} = 0.107 \pm 0.012$ M^{-1} s^{-1}.

Radiosynthesis of [$^{188}Re(H_2O)_3(CO)_3]^+$

Scheme S3. Radiosynthesis of [$^{188}Re(H_2O)_3(CO)_3]^+$.

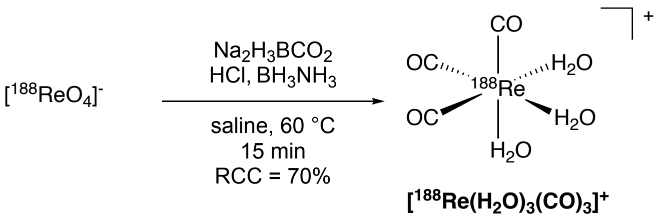

The radiolabeling reaction to prepare [$^{188}Re(H_2O)_3(CO)_3]^+$ was accomplished by the addition of 1 mL of [$^{188}ReO_4]^-$ stock solution containing 20.5 mL of 37% HCl (250 mmol) to a N_2 flushed vial containing 5 mg BH_3NH_3 and 5 mg $Na_2H_3BCO_2$ with a total reaction volume of 1.00 mL. A test reaction was performed with 10 MBq of [$^{188}ReO_4]^-$. The reaction was heated to 60 °C for 15 min. The reaction was monitored by analytical radio-HPLC, giving a radiochemical conversion (RCC) 70% (n = 1) (R_t = 5.50 min). The product was characterized by analytical HPLC following the method described in the general section.

Figure S7. Radio-HPLC chromatograms for [$^{188}Re(H_2O)_3(CO)_3]^+$ and [$^{188}ReO_4]^-$.

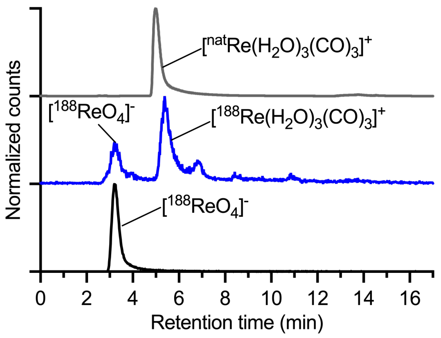

Synthesis and Characterization

Scheme S4. Overall sequence for the synthesis of photoactivatable ligand L1.

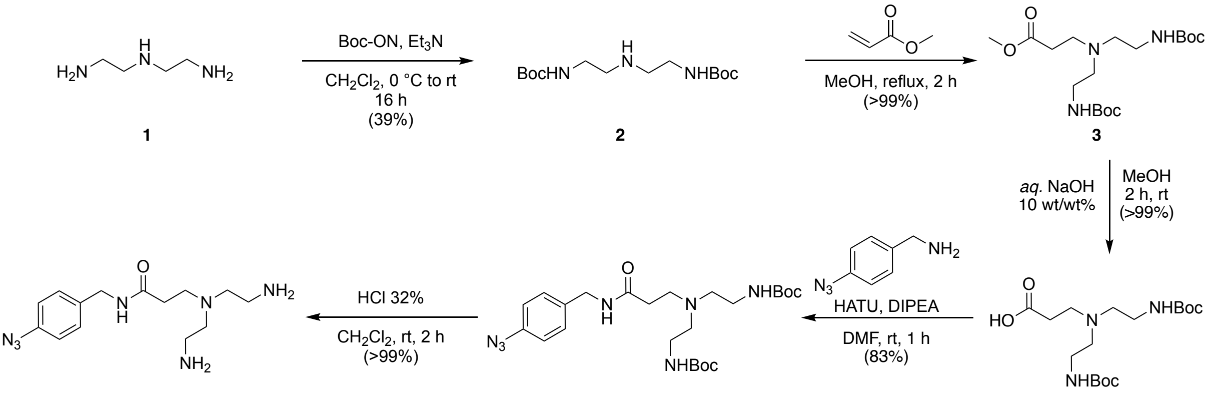

Scheme S5. Synthesis of compound 7

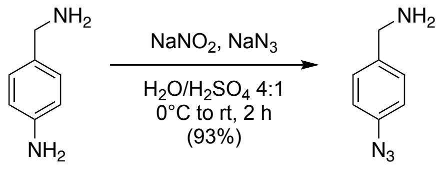

Compound 7

4-aminobenzylamine (6, 5.07 g, 41.5 mmol, 1.0 equiv.) was dissolved in H_2O (100 mL) and cooled to 0 °C and conc. sulfuric acid (22.1 mL, 415 mmol, 10.0 equiv.) was added slowly. $NaNO_3$ (3.13 g, 45.3 mmol, 1.1 equiv., dissolved in 20 mL H_2O) was added and the mixture was stirred at 0 °C for 20 min. Then NaN_3 (2.92 g, 44.7 mmol, 1.1 equiv. dissolved in 30 mL H_2O) was added dropwise, whereby the formation of N_2 from the diazonium salt was observed. When the gas formation ceased, the mixture was warmed to 25 °C and extracted with CH_2Cl_2 (3 × 100 mL) and the solvent was removed at reduced pressure to give 4-azidobenzylamine (5.69 g, 41.3 mmol, 93%) as a yellow oil. The product forms carbamates with CO_2 upon prolonged exposure to air and should be stored in the freezer under N_2. ^{1}H-NMR (400 MHz, DMSO-*d6*, 298 K) δ 7.37 (d, *J* = 8.3 Hz, 10H), 7.04 (d, *J* = 8.4 Hz, 8H), 3.70 (s, 9H) ppm. ^{13}C{^{1}H}-NMR (101 MHz, DMSO, 298 K) d 141.37, 137.11, 128.62, 118.72, 45.01 ppm. HR-ESI-MS: *m/z* calcd. for $[C_7H_8N_4+H]^+$ 149.0822, found 149.0823 (100%). These data match with the literature.[4]

Figure S8. ^{1}H (400 MHz, DMSO-*d6*, 298 K) NMR spectrum of compound 7.

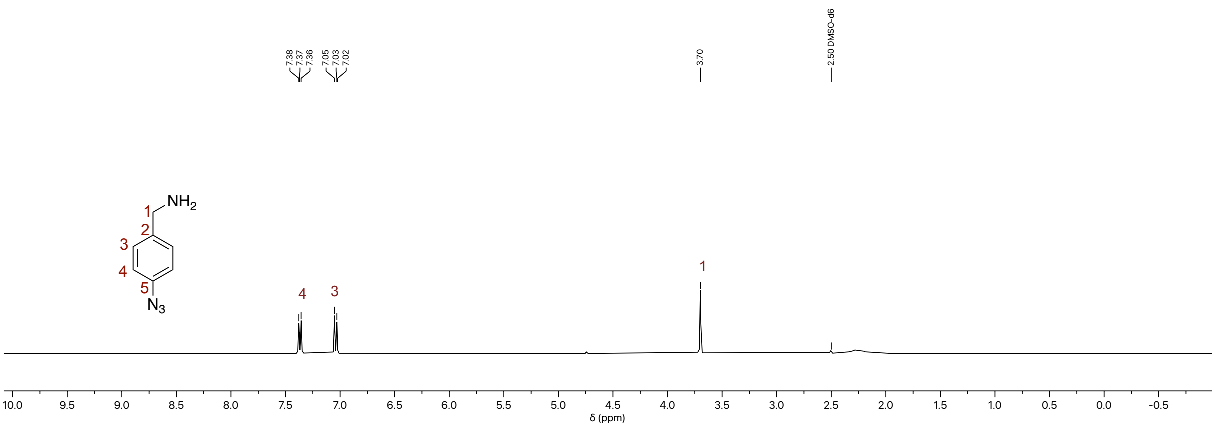

Figure S9. $^{13}C\{^{1}H\}$ (101 MHz, DMSO-d_6, 298 K) NMR spectrum of compound 7.

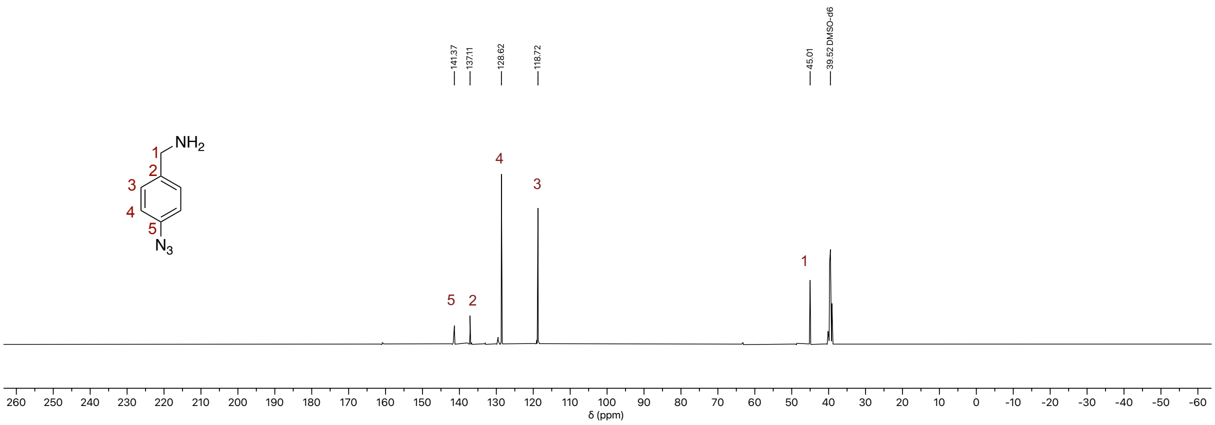

Figure S10. $^{13}C\{^{1}H\}$-DEPT-135 (101 MHz, DMSO-d_6, 298 K) NMR spectrum of compound 7.

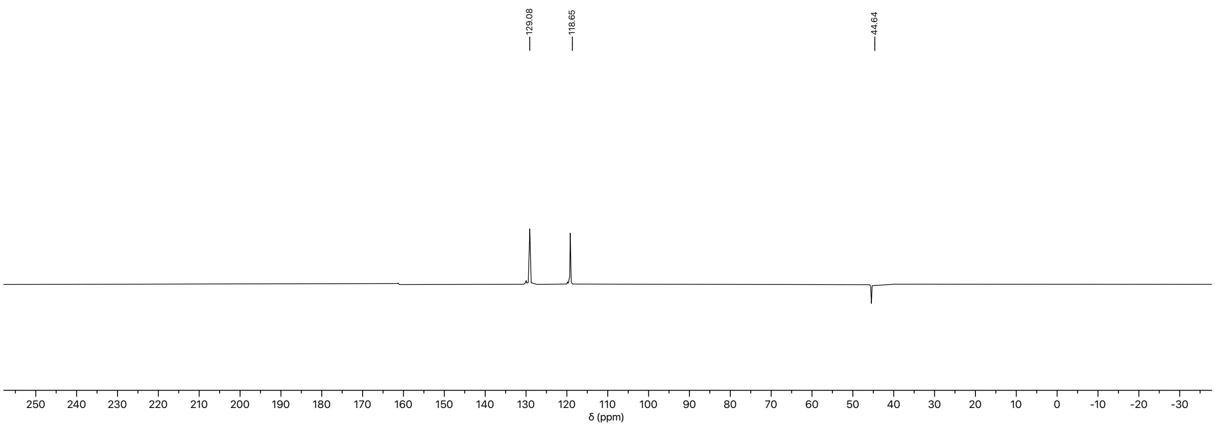

Figure S11. HRMS (ESI+) spectrum of compound 7.

Note: The peak 136.0758 is $[M+H-N_2+H_2O]^+$.

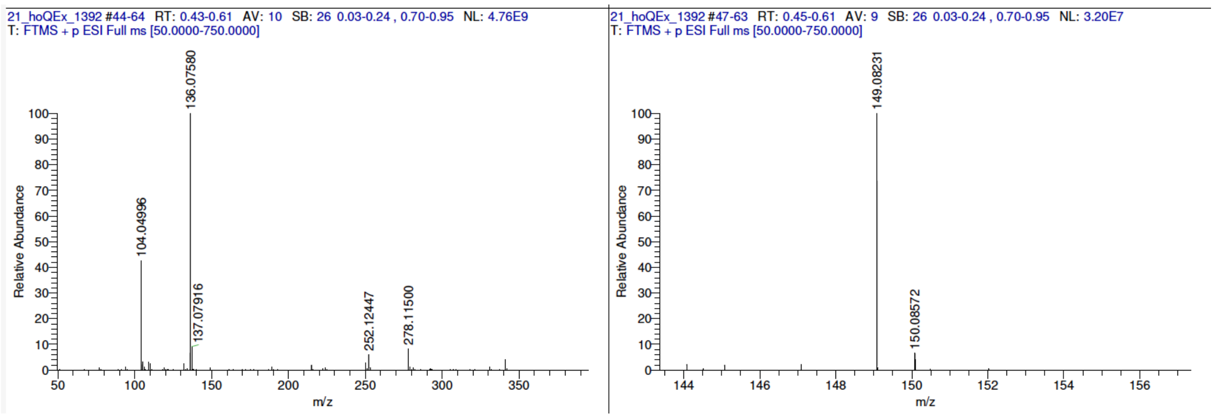

Scheme S6. Synthesis of compound 2.

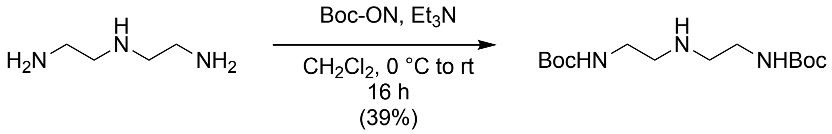

Compound 2

Diethylenetriamine (1.00 g, 9.69 mmol, 1.0 equiv.) was dissolved in THF (20 mL), Et_3N (2.72 mL, 19.4 mmol, 2.0 equiv.) added and cooled to 0°C. Boc-ON (1.96 g, 19.4 mmol, 2.0 equiv.) dissolved in THF (50 mL) was added dropwise. The reaction mixture was stirred at 0 °C upon complete addition of Boc-ON, followed by stirring at rt for 16 h. The solvent was removed under reduced pressure, redissolved in CH_2Cl_2 (200 mL) and the solution was washed with NaOH (aq., 10% wt/wt, 2 × 100 mL), Water (2 x 100 mL) and Brine (1 × 100 mL). The organic phase was dried over Na_2SO_4 and the solvent removed. The crude was purified by flash chromatography (SiO_2, CH_2Cl_2/MeOH 90/10). Fractions containing the desired compound, as determined by spotting test on a TLC plate and staining with the Dragendorff's reagent, were combined and the solvent was removed. Compound 2 was obtained as a clear, colorless oil (39%). ^{1}H NMR: ($CDCl_3$, 400 MHz, 298 K) δ 5.18 (s, 2H), 3.11 (q, J = 6.0, 5.1 Hz, 4H), 2.63 (t, J = 5.9 Hz, 4H), 1.34 (s, 18H) ppm. ^{13}C{^{1}H} NMR: ($CDCl_3$, 101 MHz, 298 K) δ 156.19, 78.99, 48.76, 40.23, 28.37 ppm. HR-ESI-MS: *m/z* calcd. for $[C_{14}H_{29}N_3O_4+H]^+$ 304.2231, found 304.2230 (28%).

Figure S12. ^{1}H (400 MHz, $CDCl_3$, 298 K) NMR spectrum of compound 2.

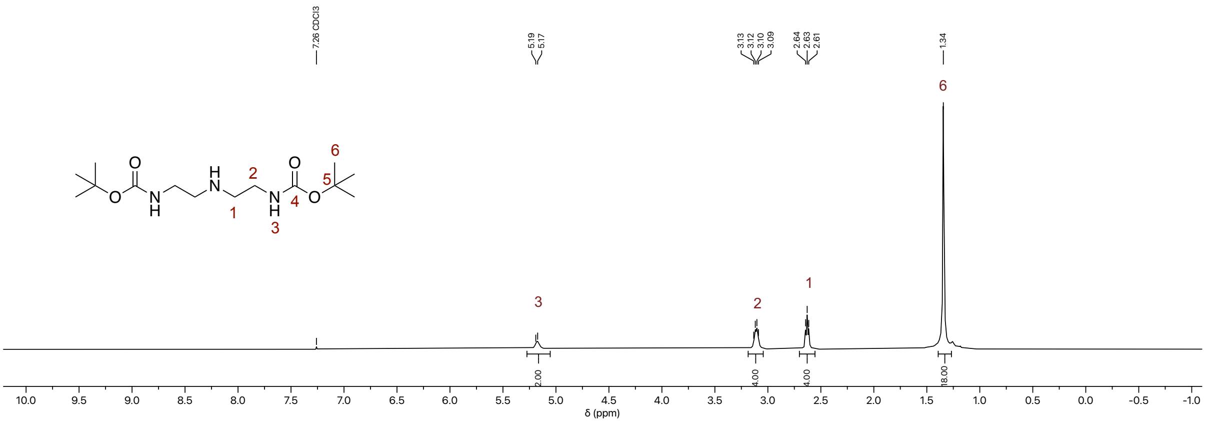

Figure S13. $^{13}C\{^{1}H\}$ (101 MHz, $CDCl_3$, 298 K) NMR spectrum of compound 2.

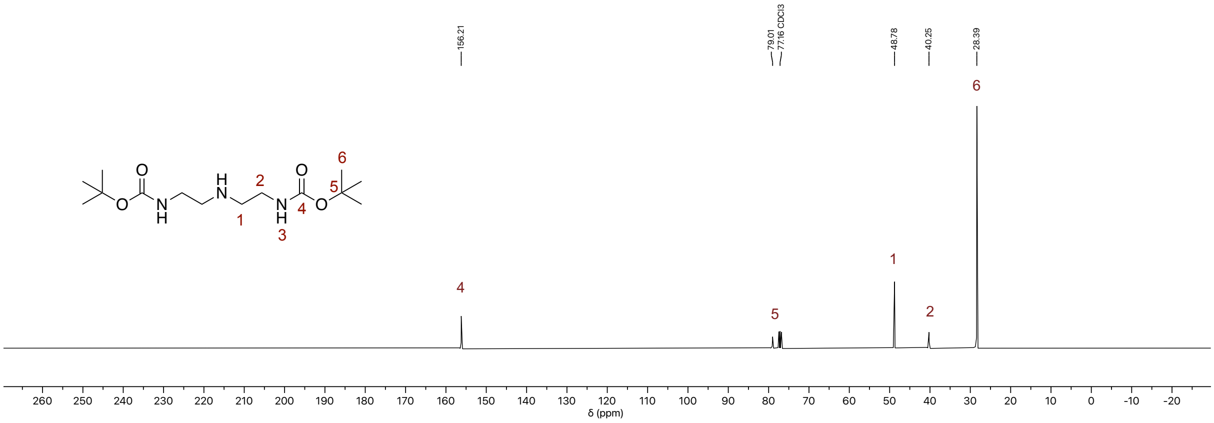

Figure S14. $^{13}C\{^{1}H\}$-DEPT-135 (101 MHz, $CDCl_3$, 298 K) NMR spectrum of compound 2.

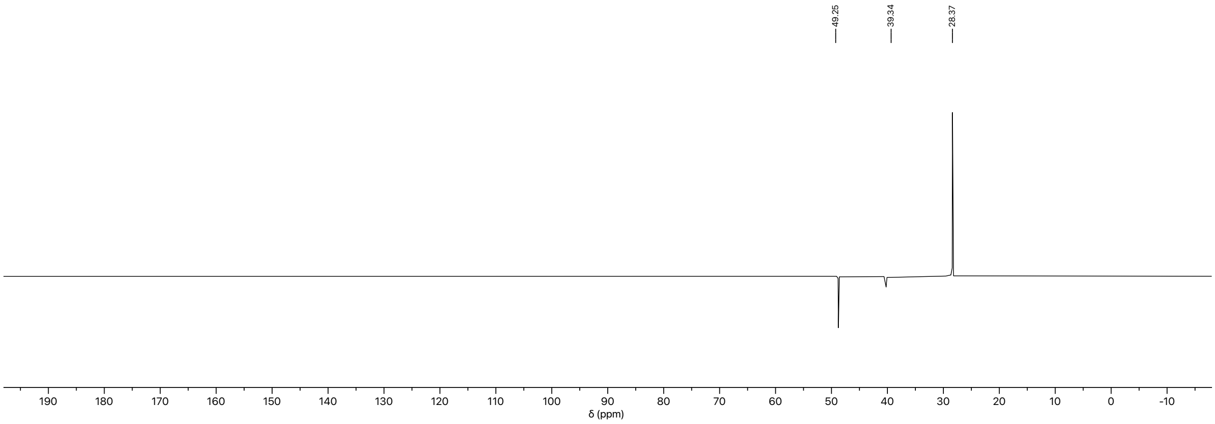

Figure S15. HRMS (ESI+) spectrum of compound 2.

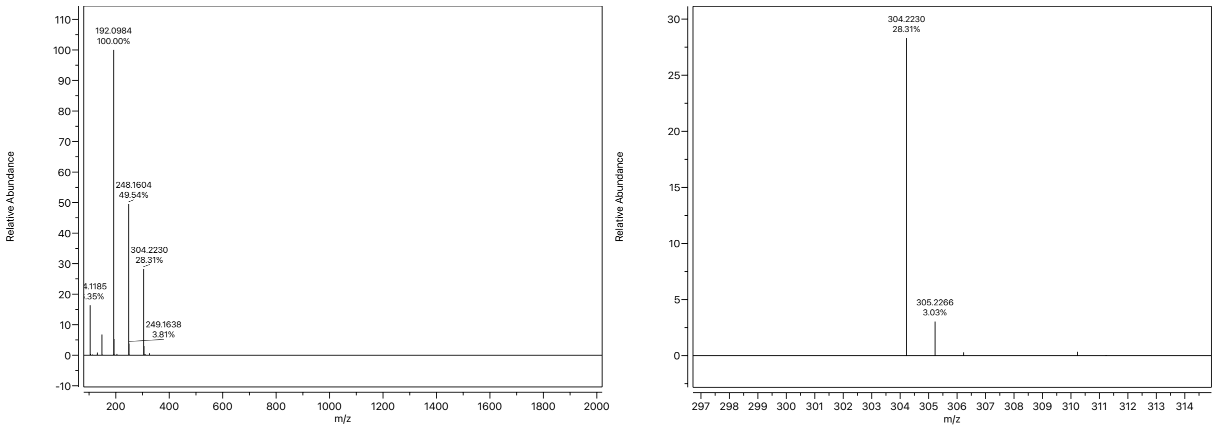

Scheme S7. Synthesis of compound 3.

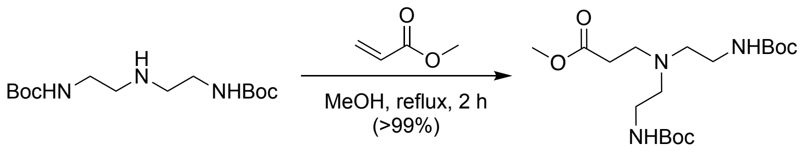

2 **3**

Compound 3

Compound 2 (801 mg, 2.64 mmol, 1.0 equiv.) was dissolved in MeOH (26 mL) and methyl acrylate (9.51 mL, 106 mmol, 40 equiv.) added. The solution was heated under reflux for 2 h, and then the reaction was cooled before removing the solvent and excess methyl acrylate under reduced pressure to obtain compound 3 (1028 mg, 2.64 mmol, >99%) as a clear colorless oil which was used without further purification. ^{1}H NMR: ($CDCl_3$, 400 MHz, 298 K) δ 5.01 (s, 2H), 3.72 (s, 3H), 3.15 (q, *J* = 5.8 Hz, 4H), 2.75 (t, *J* = 6.5 Hz, 2H), 2.52 (t, *J* = 5.8 Hz, 4H), 2.44 (t, *J* = 6.5 Hz, 2H), 1.45 (s, 18H) ppm. ^{13}C{^{1}H} NMR: ($CDCl_3$, 101 MHz, 298 K) δ 156.21, 79.01, 48.78, 40.25, 28.39 ppm. HR-ESI-MS: *m/z* calcd. for $[C_{18}H_{35}N_3O_6+H]^+$ 390.2599, found 390.2601 (29%).

Figure S16. ^{1}H (400 MHz, $CDCl_3$, 298 K) NMR spectrum of compound 3.

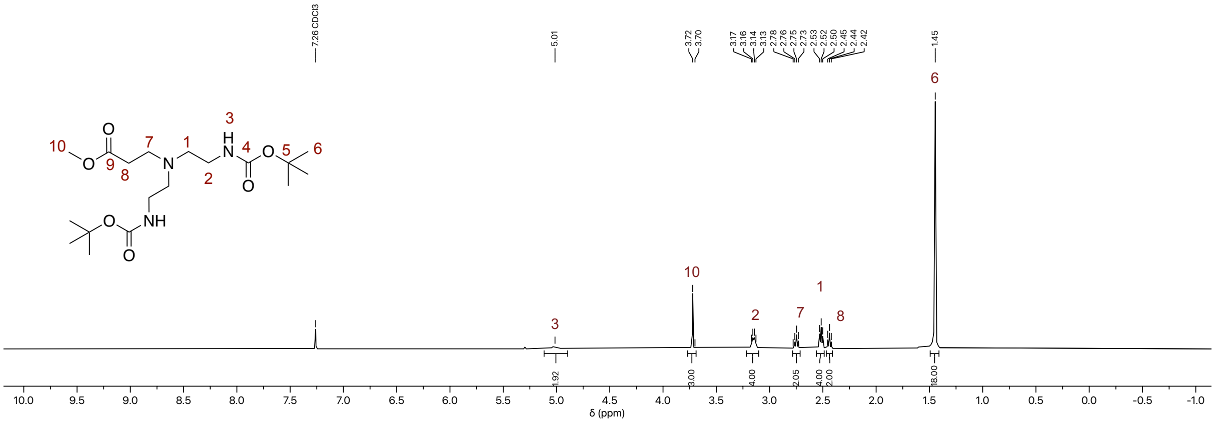

Figure S17. ^{13}C{^{1}H} (101 MHz, $CDCl_3$, 298 K) NMR spectrum of compound 3.

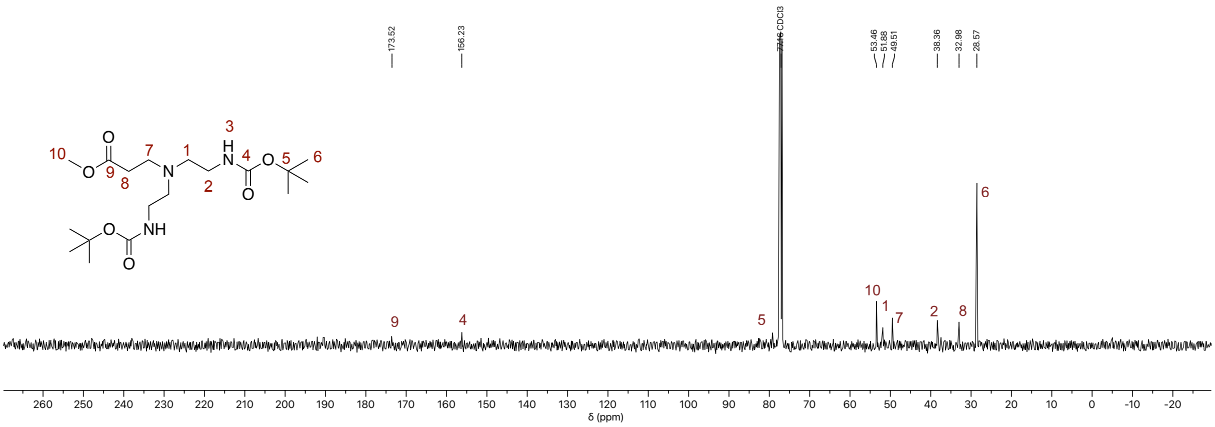

Figure S18. $^{13}C\{^1H\}$-DEPT-135 (101 MHz, $CDCl_3$, 298 K) NMR spectrum of compound 3.

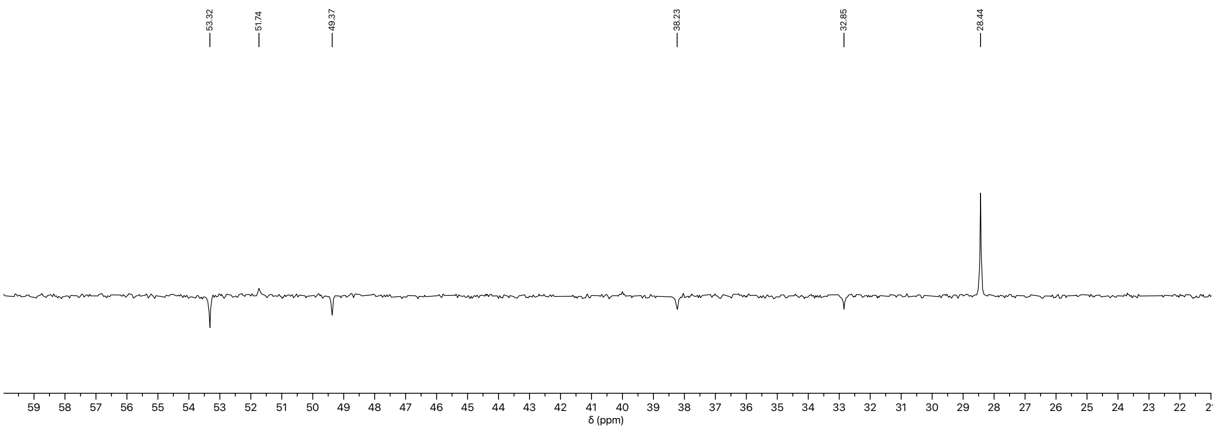

Figure S19. HRMS (ESI+) spectrum of compound 3.

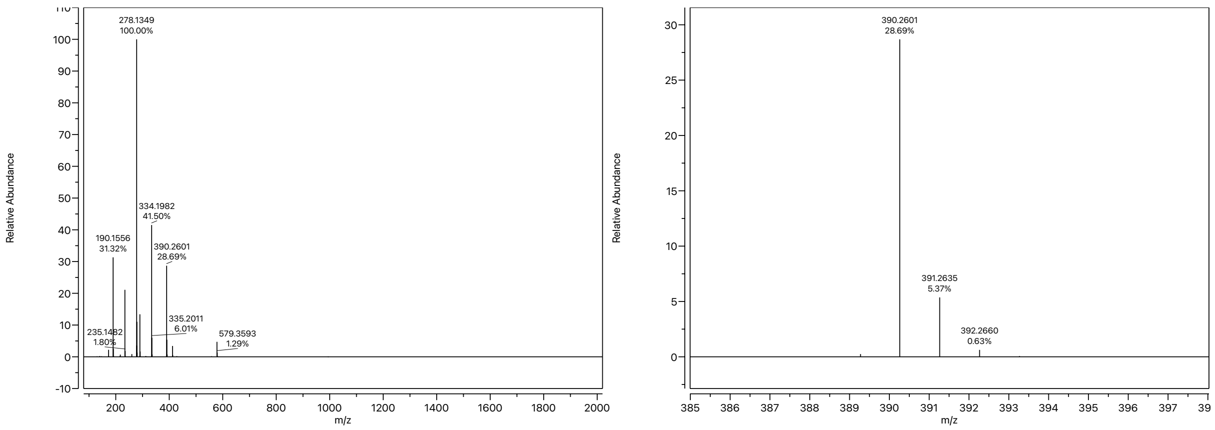

Scheme S8. Synthesis of compound 4.

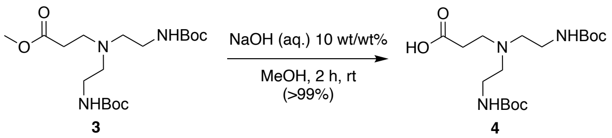

Compound 4

Compound 3 (1.03 g, 2.64 mmol, 1.0 equiv.) was dissolved in MeOH (10 mL) and NaOH aq. (10% wt/wt, 3 mL) was added. The solution was stirred for 2 h and the solvent removed. The crude was purified by flash chromatography (C18, H_2O/MeOH 95/5 to 0/100). Fractions containing the desired compound were combined and the solvent was removed under reduced pressure. Compound 4 was obtained as a clear, colorless oil (990 mg, 2.64 mmol, >99%). ^{1}H NMR: ($CDCl_3$, 400 MHz, 298 K) δ 3.12 (t, J = 6.8 Hz, 4H), 2.80 – 2.71 (m, 2H), 2.57 (t, J = 6.8 Hz, 4H), 2.34 – 2.26 (m, 2H), 1.38 (s, 18H) ppm. $^{13}C\{^{1}H\}$ NMR: ($CDCl_3$, 101 MHz, 298 K) δ 181.24, 157.98, 80.83, 52.22, 50.38, 37.53, 34.36, 27.72 ppm. HR-ESI-MS: *m/z* calcd. for $[C_{17}H_{33}N_3O_6+H]^+$ 376.2442, found 376.2450 (39%).

Figure S20. ^{1}H (400 MHz, D_2O, 298 K) NMR spectrum of compound 4.

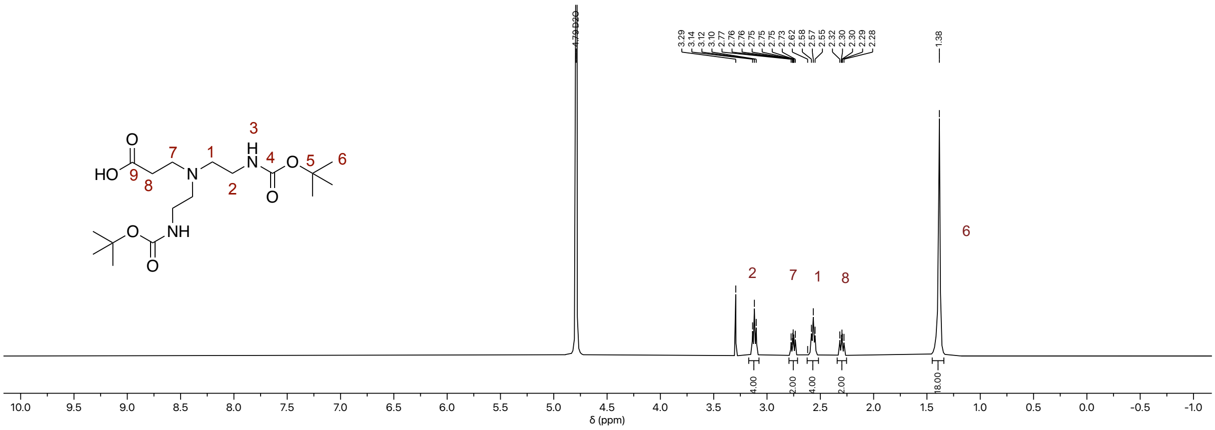

Figure S21. $^{13}C\{^{1}H\}$ (101 MHz, D_2O, 298 K) NMR spectrum of compound 4.

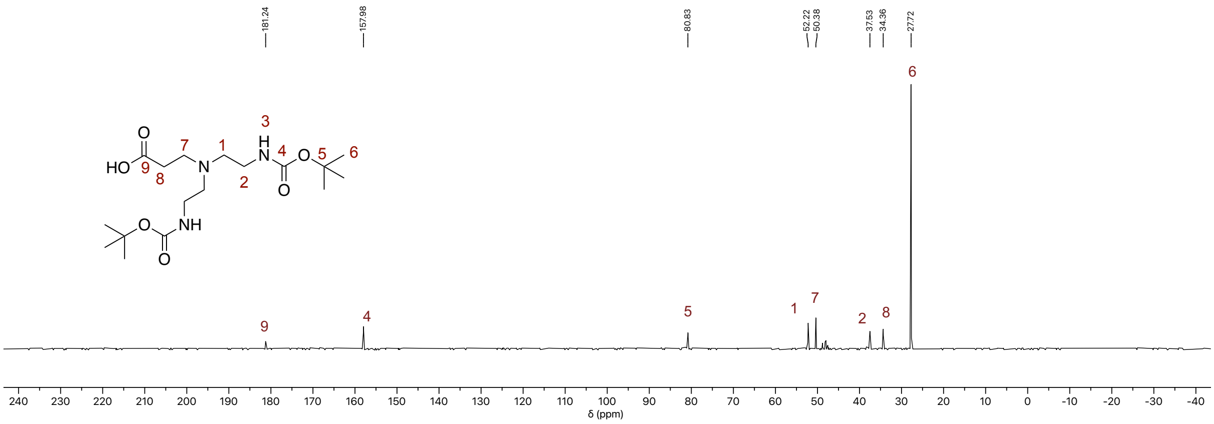

Figure S22. $^{13}C\{^1H\}$-DEPT-135 (101 MHz, D_2O, 298 K) NMR spectrum of compound 4.

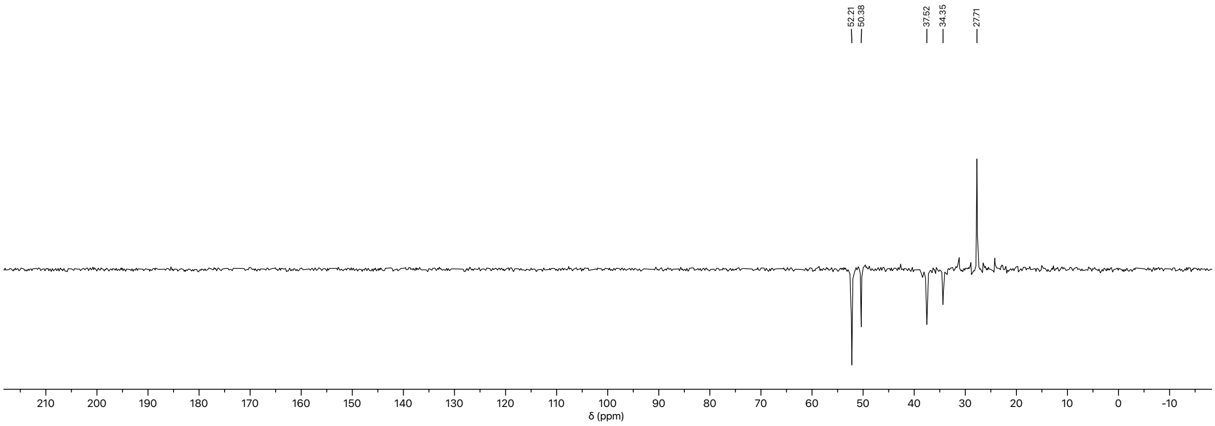

Figure S23. HRMS (ESI+) spectrum of compound 4.

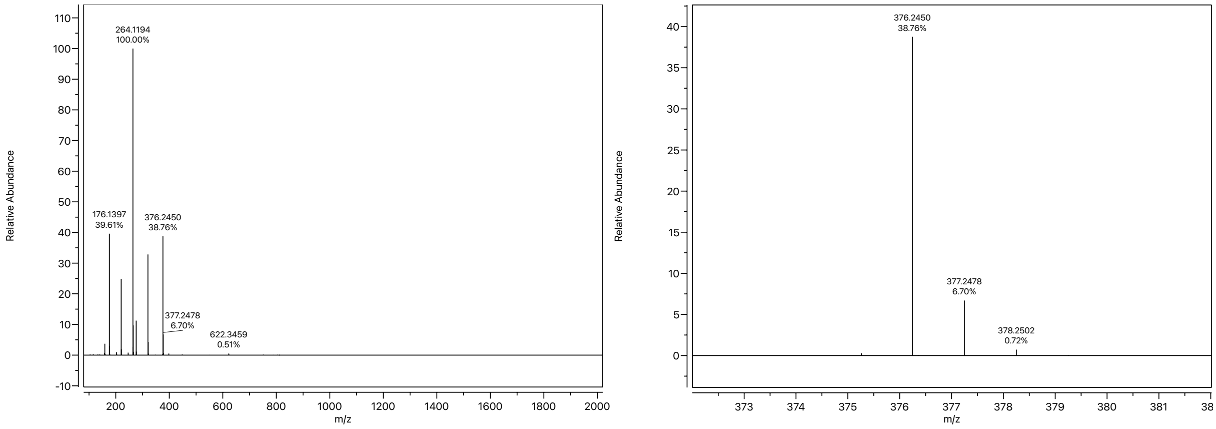

Scheme S9. Synthesis of compound 5.

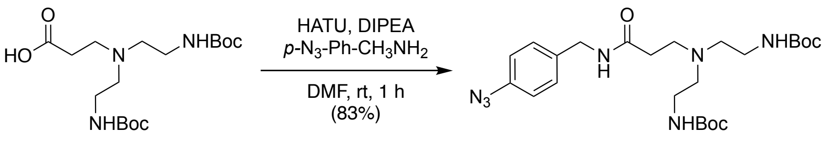

4 **5**

Compound 5

Compound 4 (384 mg, 1.02 mmol, 1.0 equiv.) was dissolved in DMF (5 mL) and HATU (428 mg, 1.13 mmol, 1.1 equiv.), HOBt (152 mg, 1.13 mmol, 1.1 equiv.) followed by DIPEA (0.71 mL, 4.09 mmol, 4.0 equiv.) added and the mixture stirred for 10 min. 4-Azidobenzylamine (182 mg, 1.23 mmol, 1.2 equiv.) was added dropwise in DMF (1 mL) and stirred for 1 h. Water (5 mL) and NaOH aq. (10% wt/wt, 5 mL) was added to the reaction mixture and stirred for 5 min. The mixture was extracted with CH_2Cl_2 (3 x 30 mL). The combined organic phases were dried over Na_2SO_4 and the solvent removed under reduced pressure. The crude was purified by flash chromatography (C18, H_2O/MeOH 95/5 to 0/100). Fractions containing the desired compound were combined and the solvent was removed under reduced pressure. Compound 5 was obtained as a yellowish solid (430 mg, 0.85 mmol, 83%). ^1H NMR: ($CDCl_3$, 400 MHz, 298 K) δ 7.41 (t, J = 6.0 Hz, 1H), 7.23 (d, J = 8.1 Hz, 2H), 6.91 (d, J = 8.4 Hz, 2H), 5.13 (s, 1H4.35 (d, J = 5.9 Hz, 2H), 3.05 (d, J = 6.1 Hz, 4H), 2.70 (t, J = 6.2 Hz, 2H), 2.47 (t, J = 6.0 Hz, 4H),

2.27 (t, J = 6.5 Hz, 2H), 1.36 (s, 19H) ppm. $^{13}C\{^1H\}$ NMR: (CD_3OD, 101 MHz, 298 K) δ 171.14, 157.62, 139.00, 135.14, 128.99, 118.82, 79.91, 53.88, 50.77, 42.20, 35.25, 27.42 ppm. HR-ESI-MS: *m/z* calcd. for $[C_{24}H_{39}N_7O_5+H]^+$ 506.3085, found 506.3076 (100%).

Figure S24. ^1H (400 MHz, $CDCl_3$, 298 K) NMR spectrum of compound 5.

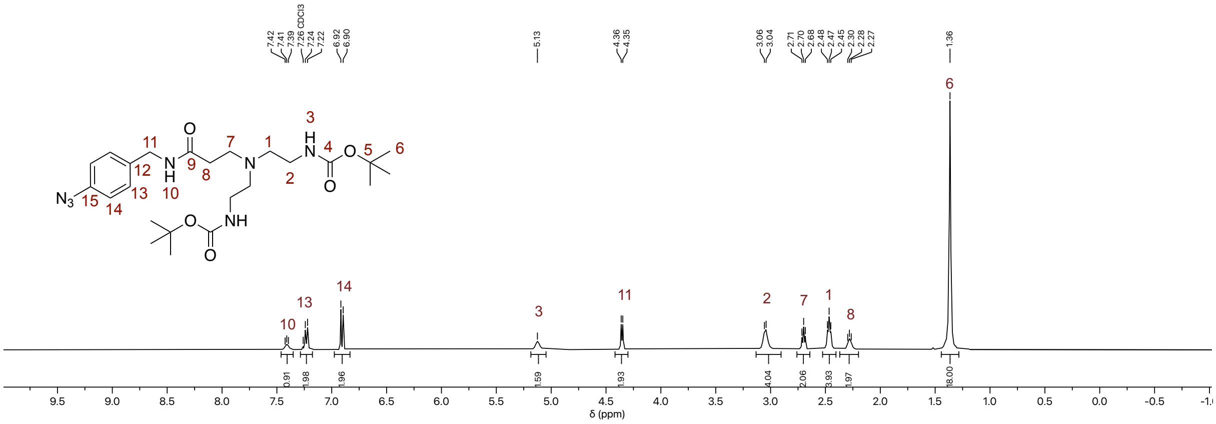

Figure S25. $^{13}C\{^1H\}$ (101 MHz, CD_3OD, 298 K) NMR spectrum of compound 5.

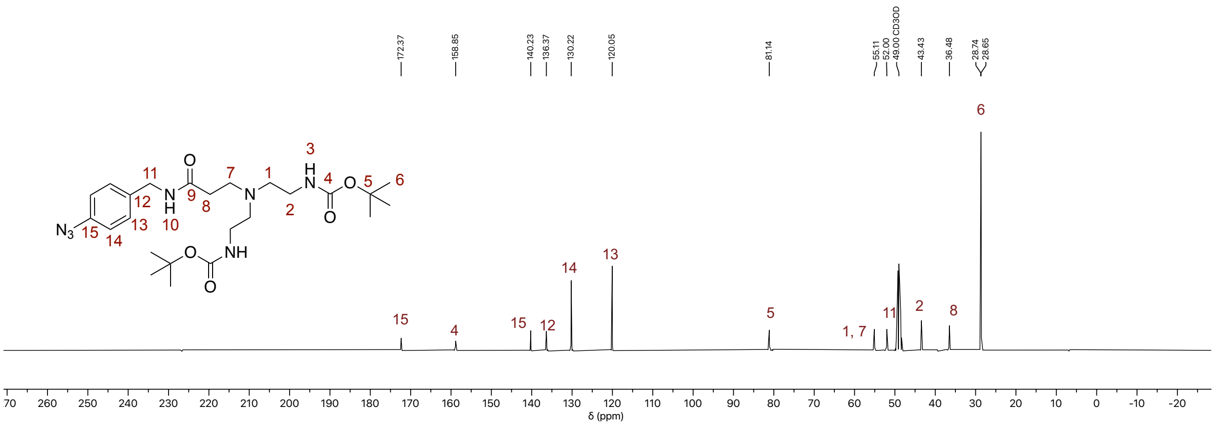

Figure S26. $^{13}C\{^1H\}$-DEPT-135 (101 MHz, CD_3OD, 298 K) NMR spectrum of compound 5.

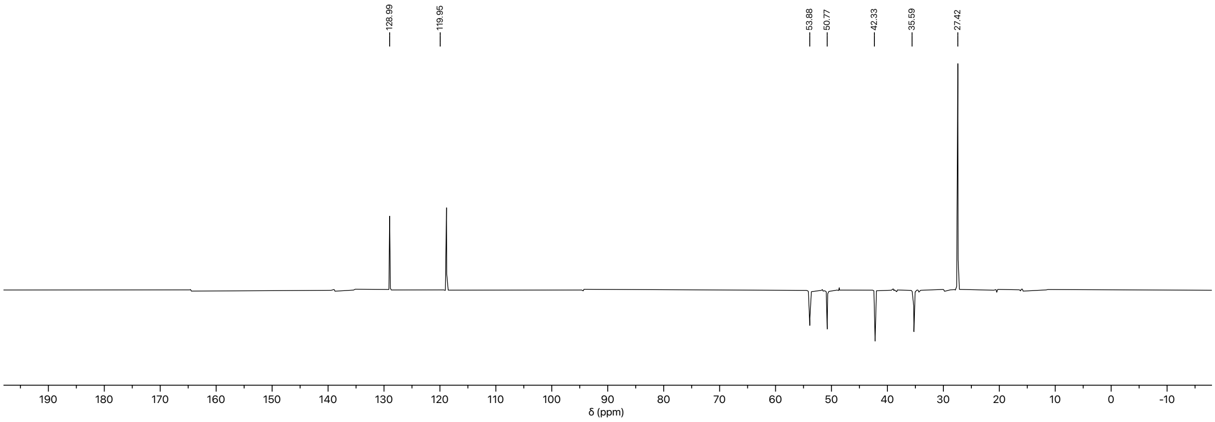

Figure S27. HRMS (ESI+) spectrum of compound 5.

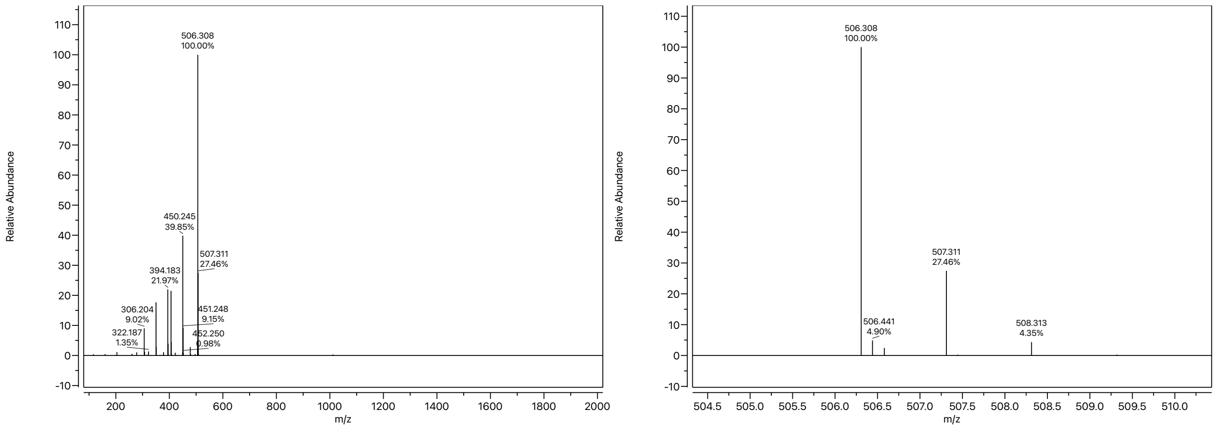

Scheme S10. Synthesis of compound L1.

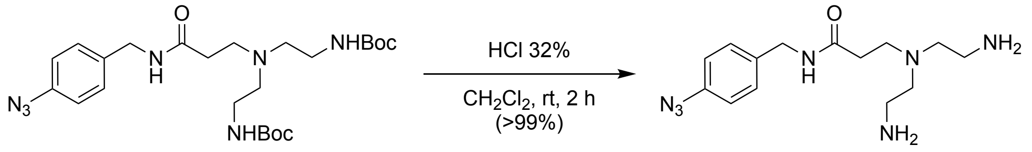

5 **L1**

Compound L1

Compound 5 (430 mg, 0.85 mmol, 1.0 equiv.) was dissolved in CH_2Cl_2 (2 mL) and 32% HCl (1 mL) was added. The mixture was vigorously stirred at rt for 2 h and the solvent was carefully removed under reduced pressure. The residue was dissolved in H_2O, the pH adjusted to 10, and then the mixture was purified by flash chromatography (C18, H_2O/MeOH 100/0 to 0/100). Fractions containing the desired compound were combined and the solvent was removed under reduced pressure. The product L1 was obtained as a yellowish solid (260 mg, 0.85 mmol, >99%). The purity was determined by HPLC and ^{1}H NMR: (D_2O, 400 MHz, 298 K) δ 7.18 (d, *J* = 8.5 Hz, 2H), 6.94 (d, *J* = 8.5 Hz, 2H), 4.19 (s, 2H), 2.91 (t, *J* = 6.3 Hz, 4H), 2.71 (t, *J* = 6.8 Hz, 2H), 2.63 (t, *J* = 6.3 Hz, 4H), 2.32 (t, *J* = 6.8 Hz, 2H) ppm. $^{13}C\{^1H\}$ NMR: (D_2O, 101 MHz, 298 K) δ 171.85, 139.00, 134.34, 128.93, 119.16, 50.07, 49.92, 42.65, 34.26, 29.47 ppm. HR-ESI-MS: *m/z* calcd. for $[C_{14}H_{23}N_7O+H]^+$ 506.2037, found 506.2040 (100%).

Figure S28. ^{1}H (400 MHz, D_2O, 298 K) NMR spectrum of compound L1.

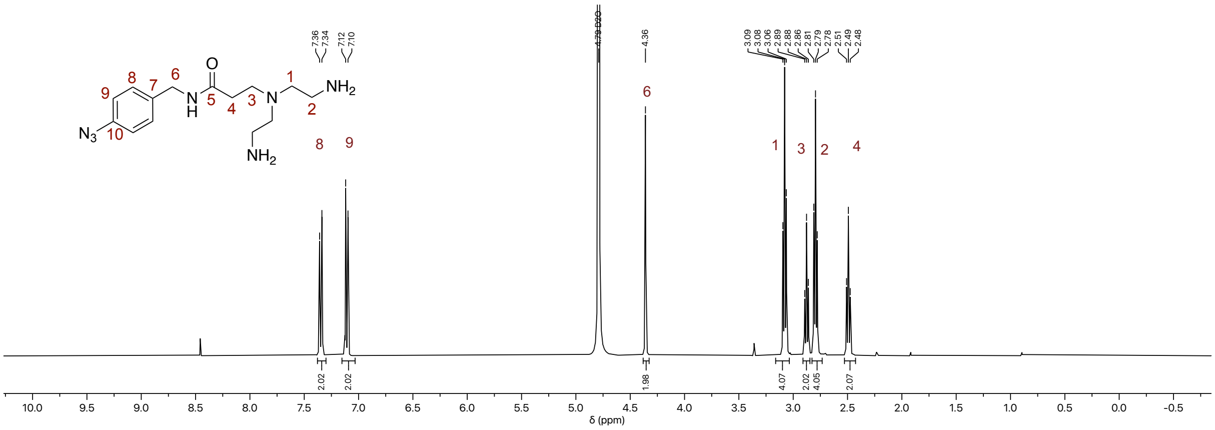

Figure S29. $^{13}C\{^{1}H\}$ (101 MHz, D_2O, 298 K) NMR spectrum of compound L1.

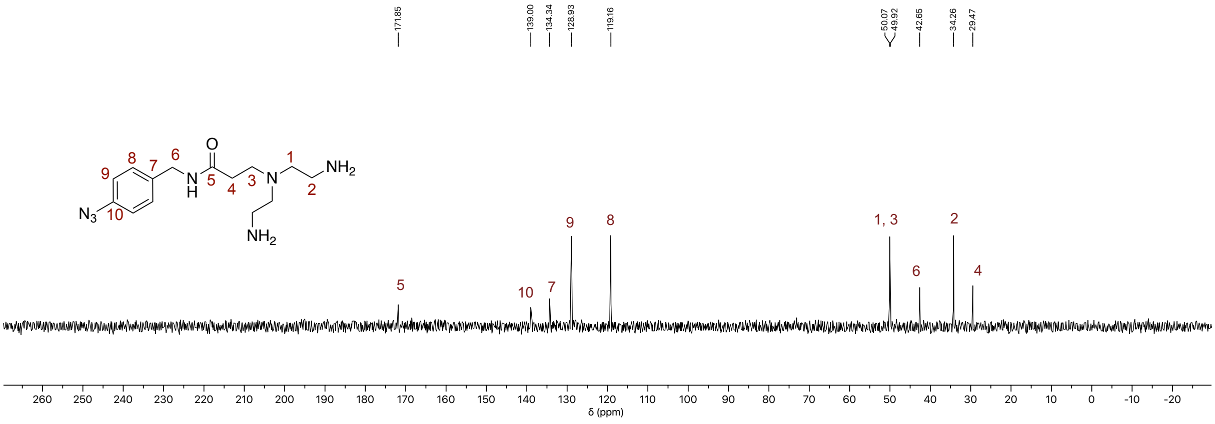

Figure S30. $^{13}C\{^{1}H\}$-DEPT-135 (101 MHz, D_2O, 298 K) NMR spectrum of compound L1.

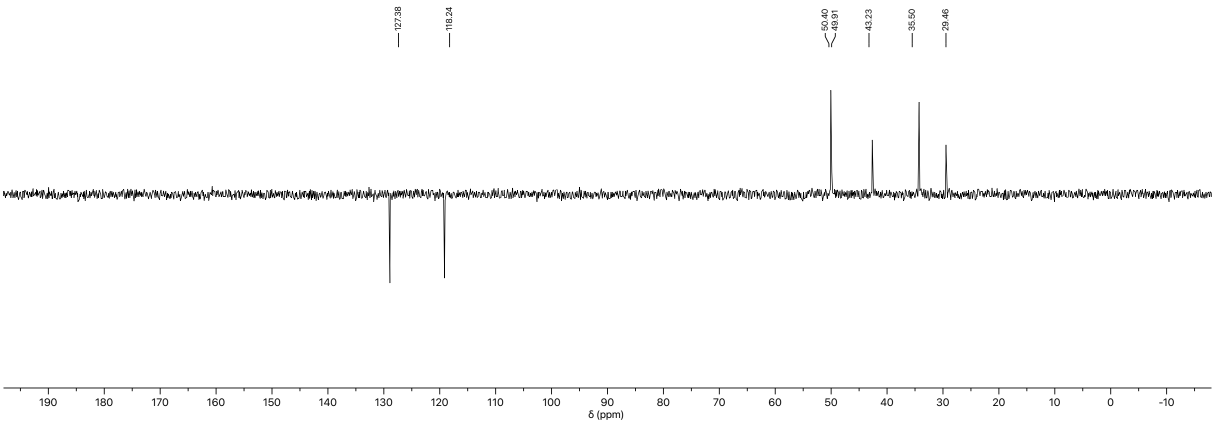

Figure S31. HRMS (ESI+) spectrum of compound L1.

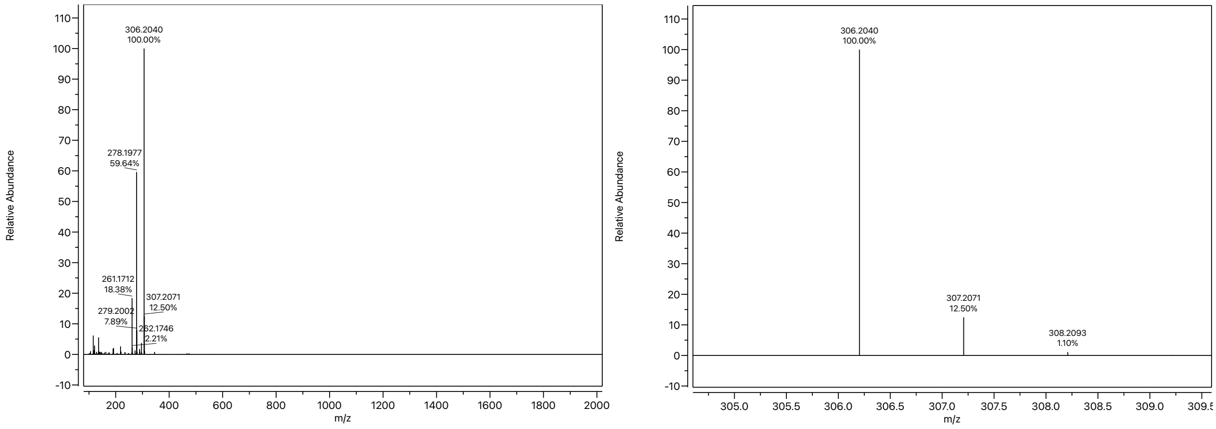

Figure S32. Reverse-phase analytical HPLC chromatogram of ligand L1, λ = 254 nm.

Retention time = 13.28 min.

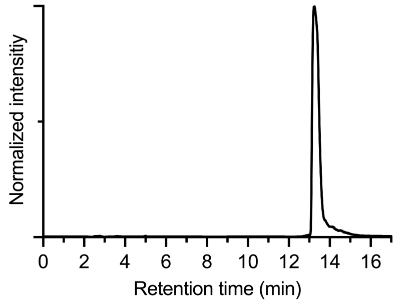

Scheme S11. Synthesis of ^{nat}Re-L1$^+$.

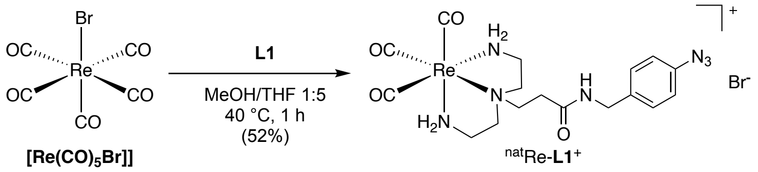

^{nat}Re-L1$^+$ complex

[Re(CO)$_5$Br] (30.0 mg, 73.9 mmol, 1.0 equiv.) was dissolved in THF (0.5 mL) and L1 (22.6 mg, 73.9 mmol, 1.0 equiv.) in MeOH (0.1 mL) was added. The solution was stirred at 40 °C for 1 h. The solvent was removed under reduced pressure and the crude was purified by flash column chromatography (C18, H_2O/MeOH 100/0 to 0/100). Fractions containing the desired compound were combined and the solvent was removed under reduced pressure. The product was obtained as a white solid (25.0 mg, 38.1 mmol, 52%) with a purity of 91% as determined by HPLC (λ = 254 nm). ^{1}H NMR: (CD_3OD , 400 MHz, 298 K) δ 7.35 (d, J = 8.5 Hz, 2H), 7.05 (d, J = 8.5 Hz, 2H), 4.38 (s, 2H), 3.81 (t, J = 7.5 Hz, 2H), 3.27 – 3.05 (m, 4H), 3.05 – 2.86 (m, 4H), 2.75 (t, J = 7.3 Hz, 2H) ppm. ^{13}C{^{1}H} NMR: (CD_3OD, 101 MHz, 298 K) δ 159.73, 154.41, 130.44, 120.15, 63.21, 58.35, 45.06, 43.70 ppm. HR-ESI-MS: m/z calcd. for $[ReC_{17}H_{23}N_7O_4]^+$ {M^+} 576.1369, found 576.1374 (100%). FT-IR (ATR): 2114 (N_3), 2020 (CO), 1884 (CO).

Figure S33. ^{1}H (400 MHz, CD_3OD, 298 K) NMR spectrum of $^{nat}Re-L1^{+}$.

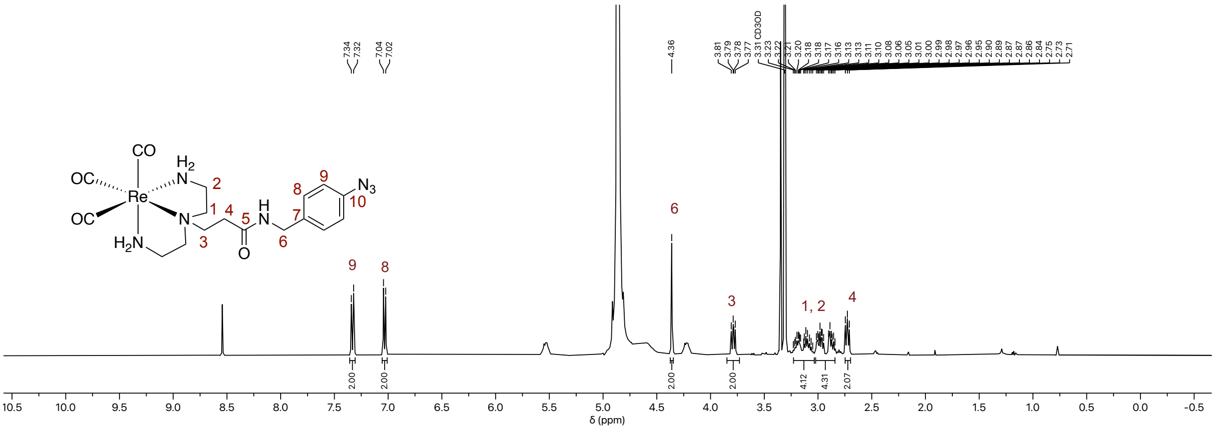

Figure S34. $^{13}C\{^{1}H\}$ (400 MHz, CD_3OD, 298 K) NMR spectrum of $^{nat}Re-L1^{+}$.

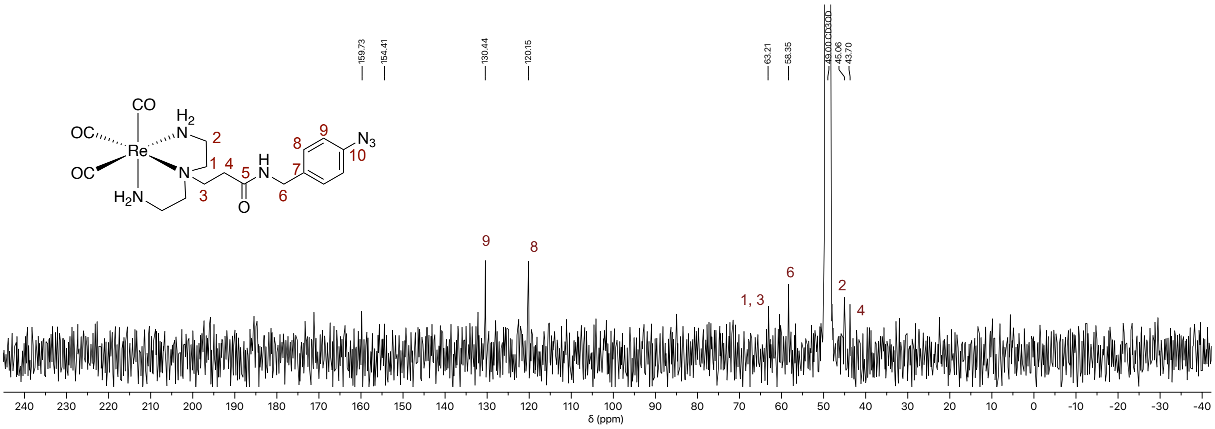

Figure S35. $^{13}C\{^{1}H\}$-DEPT-135 (101 MHz, CD_3OD, 298 K) NMR spectrum of $^{nat}Re-L1^{+}$.

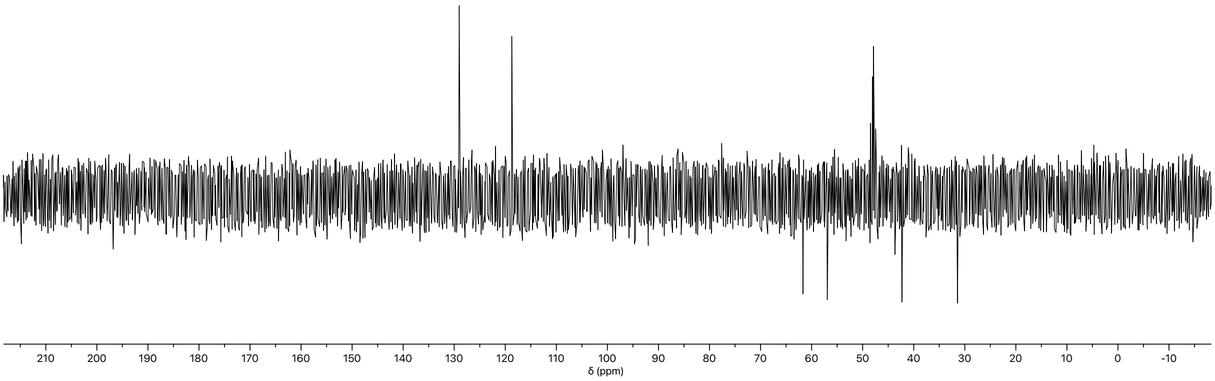

Figure S36. Reverse-phase analytical HPLC chromatogram of ^{nat}Re-L1^{+}.

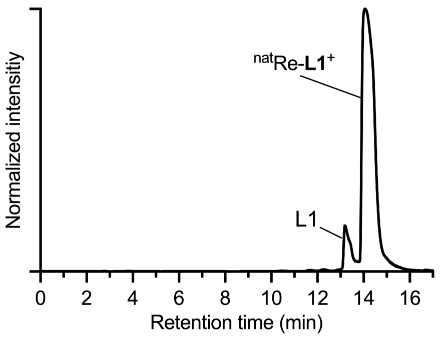

Figure S37. HRMS (ESI+) spectrum of ^{nat}Re-L1^{+}.

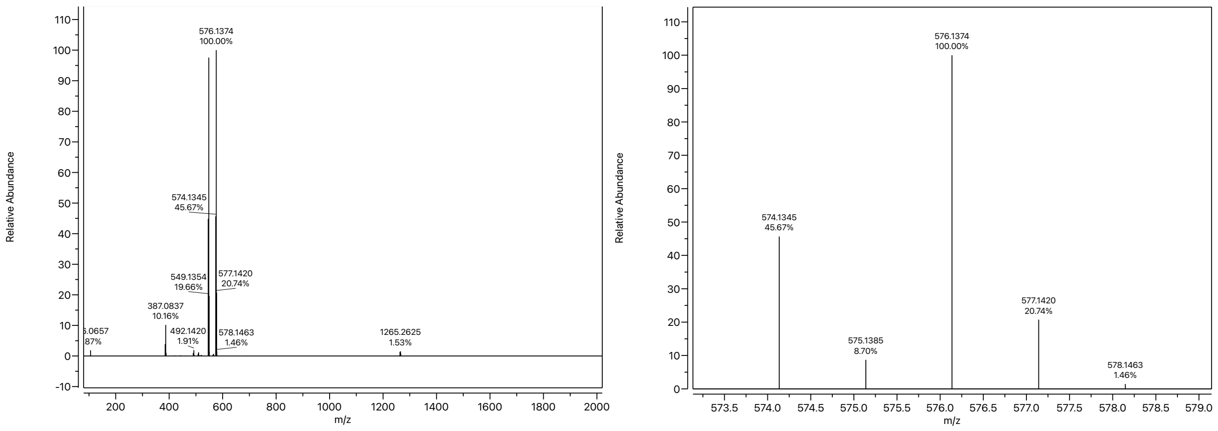

Radiosynthesis of [^{99m}Tc(CO)$_3$L1]$^{+}$ (^{99m}Tc-L1^{+})

Scheme S12. Radiosynthesis of ^{99m}Tc-L1^{+}.

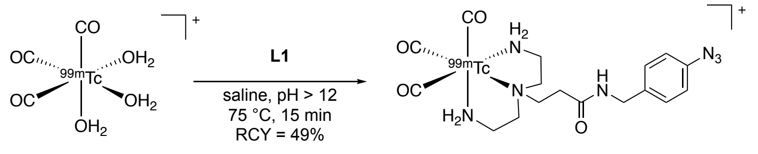

13 ^{99m}Tc-**L1**$^{+}$

Radiolabeling reactions to prepare ^{99m}Tc-L1^{+} were accomplished by the addition of 1 mL of freshly prepared [^{99m}Tc(H_2O)$_3$(CO)$_3$]$^{+}$ stock solution to an aqueous solution of L1 (243 nmol, 10 µL of 10 mg mL^{-1} stock in H_2O) with a total reaction volume of 1.01 mL. The reactions were heated to 75 °C for 15 min. Test reactions were performed with 10 to 50 MBq and reactions for the animal doses with up to 1468 MBq of [^{99m}TcO$_4$]$^{-}$. The reactions were monitored by analytical radio-HPLC and complexation was found to be complete in 15 min at 75 ºC giving a radiochemical conversion (RCC) >90% (R_t = 14.55 min). The excess ligand and side products were removed by passing the mixture through a C8-cartridge (pre-treated with 1 mL of a 1:4 mixture of acetone/ethanol followed by 5 mL of H_2O). First, the pH of the reaction mixture was adjusted to 6-6.5 (protonation of the excess ligand). The pH-adjusted mixture was slowly passed through the cartridge and the eluate discarded. The cartridge was washed with 2 mL of H_2O and blown dry by passing through 10 mL of air. The product was eluted with 1 mL of a 1:3 acetone/ethanol mixture. The solvent was carefully removed via rotary evaporation and reformulated in the desired media. The complex ^{99m}Tc-L1^{+} was obtained in a RCP >99%, RCY >49% (n = 1) and molar activity of

~2.96 MBq/nmol. The total reaction time was 30 min, including the purification. The product was characterized by analytical HPLC following the method described in the general section. Note: the UV-Vis detector and radioactivity detector were arranged serially with an offset time of approximately 0.40 min. The identity of the radiolabeled compound ^{99m}Tc-L1$^+$ was confirmed by co-injection with an authenticated sample of non-radioactive complex ^{nat}Re-L1$^+$.

Figure S38. Radio-HPLC chromatograms for [^{99m}Tc(H_2O)$_3$(CO)$_3$]$^+$, ^{99m}Tc-L1$^+$ and ^{nat}Re-L1$^+$.

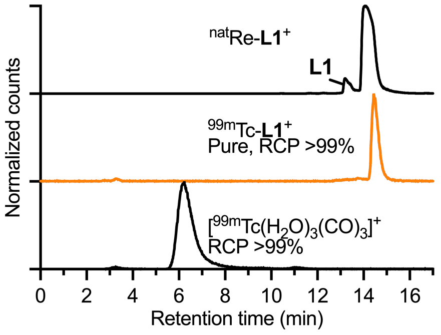

Radiosynthesis of [^{188}Re(CO)$_3$L1]$^+$ (^{188}Re-L1$^+$)

Scheme S13. Radiosynthesis of ^{188}Re-L1$^+$

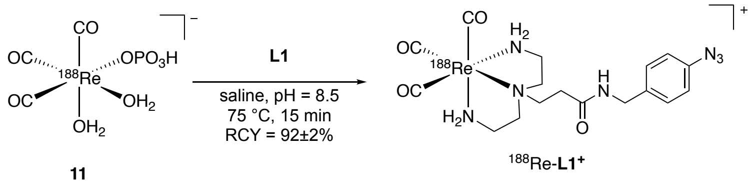

Radiolabeling reactions to prepare ^{188}Re-L1$^+$ were accomplished by the addition of 1 mL of freshly prepared [^{188}Re(HPO_4)(H_2O)$_2$(CO)$_3$]$^-$ stock solution to an aqueous solution of L1 (30 µL of 100 mg mL^{-1} stock in H_2O) under an N_2 atmosphere with a total reaction volume of 1.03 mL. Test reactions were performed with 10 to 50 MBq and reactions for the animal doses with up to 500 MBq of [^{188}Re(HPO_4)(H_2O)$_2$(CO)$_3$]$^-$ (pH8.5). The reactions were heated to 75 °C for 15 min. The reactions were monitored by analytical radio-HPLC and complexation was found to be complete in 15 min at 75 ºC giving a radiochemical conversion (RCC) >95% (R_t = 14.85 min).

The excess ligand and side products were removed as described in the following text and shown in the Supplemental Figure S41. First, the pH of the crude reaction mixture was adjusted to 6-6.5 (protonation of the excess ligand) by addition of 1 M HCl (aq.). The mixture was then, shown in Supplemental Figure S41 (R-1 and UV-1), pushed through a C8-cartridge (pre-treated with 1 mL of a 1:4 mixture of acetone/ethanol followed by 5 mL of H_2O) and the eluate discarded. The cartridge was washed with 2 mL of H_2O and blown dry by passing through 10 mL of air. The radio- and UV-HPLC traces from the eluate from the washing are shown in Supplemental Figure S41 (R-2 and UV-2). The product was then eluted with 1 mL of a 1:3 acetone/ethanol mixture, shown in Supplemental Figure S41 (R-3 and UV-3). The excess ligand could be successfully removed as

shown by comparison with normalized UV-HPLC trace of a blank sample with the UV-HPLC-trace of the acetone/ethanol elution (UV-3 and UV-4). The organic solvent was carefully removed via rotary evaporation and reformulated in the desired media.

The complex ^{188}Re-L1^{+} was obtained in a RCP <99%, RCY 92±2% (n = 2) and molar activity of ~63.1 kBq nmol^{-1} (R_t = 14.85 min). The total reaction time was 30 min, including the purification. The product was characterized by analytical HPLC following the method described in the general section. Note: The UV-Vis detector and radioactivity detector were arranged serially with an offset time of approximately 0.40 min. The identity of the radiolabeled compound (^{188}Re-L1^{+}) was confirmed by co-injection with an authenticated sample of non-radioactive complex ^{nat}Re-L1^{+}.

Figure S39. Radio-HPLC chromatograms for the precursor, ^{188}Re-L1^{+}, the natural Rhenium reference ^{nat}Re-L1^{+} and complex ^{188}Re-L1^{+} after photolysis with 395 nm light for 15 min.

Note: The peak marked with * corresponds to [^{188}Re(H_2O)$_3$(CO)$_3$]$^{+}$.

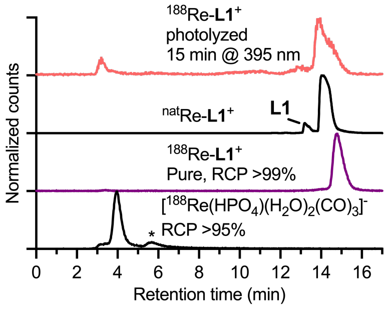

Figure S40. Normalized radio HPLC chromatograms of the eluted fractions from the purification of ^{188}Re-L1^{+} from the labeling reaction of [^{188}Re(HPO_4)(H_2O)$_2$(CO)$_3$]$^{-}$ with the ligand L1.

Note: R → Radiotrace; UV → UV-trace.

Legend: ●: [^{188}ReO_4]$^{-}$, ○: [^{188}Re(HPO_4)(H_2O)$_2$(CO)$_3$]$^{-}$, ■: [^{188}Re(H_2O)$_3$(CO)$_3$]$^{+}$,◆: [^{188}Re(CO)$_3$L1-NH_2]$^{+}$,★: [^{188}Re(CO)$_3$L1]$^{+}$, □: L1.

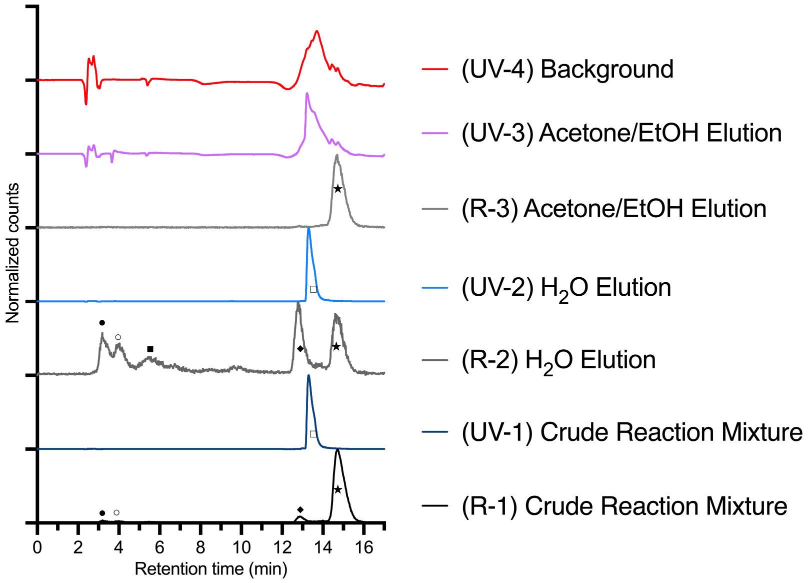

Radiochemical stability and cellular binding of ^{99m}Tc/^{188}Re-L1^{+}

Figure S41. Stability test of ^{99m}Tc-L1^{+} in PBS and challenge with 1 mM His/Cys.

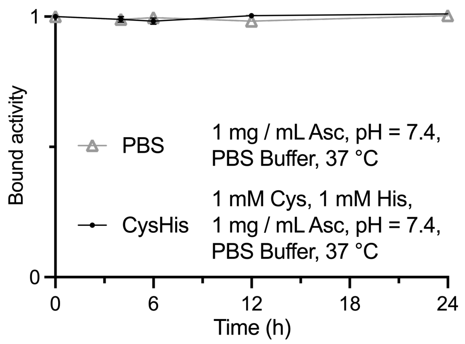

Figure S42. Stability test of ^{188}Re-L1^{+} in PBS and challenge with 1 mM His/Cys

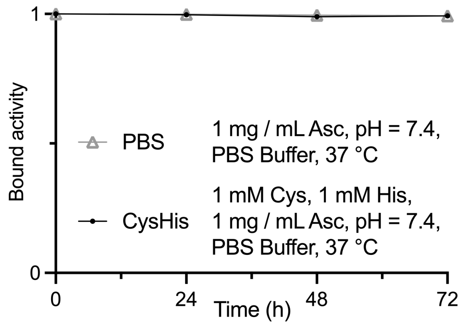

Scheme S14. Synthesis of [^{nat}Re(CO)$_3$(diethylenetriamine)]Br ([^{nat}Re(CO)$_3$(DETA)]Br)

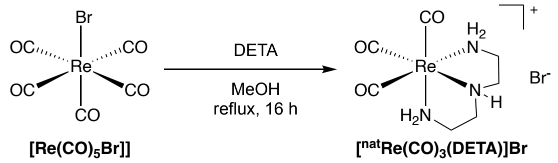

Complex [^{nat}Re(CO)$_3$(diethylentriamine)]Br

[Re(CO)$_5$Br] (100.0 mg, 246 mmol, 1.0 equiv.) was dissolved in MeOH (5.0 mL) and diethylenetriamine (106 µL, 985 mmol, 4.0 equiv.) was added. The solution was refluxed for 48 h. The solvent was removed under reduced pressure and the crude was purified by preparative HPLC (C18, 100% H_2O). Fractions containing the desired compound were combined and the solvent was removed under reduced pressure. The product was obtained as a white solid (20.0 mg, 44.1 mmol, 18%) in a purity of >95% as determined by HPLC (λ = 254 nm). HR-ESI-MS: *m/z* calcd. for $[ReC_7H_{13}N_3O_3]^+$ {M^+} 374.0514, found 374.0503 (100%). FT-IR (ATR): 2015 (CO), 1915 (CO), 1872 (CO).

Figure S43. HPLC chromatogram of compound [$^{nat}Re(CO)_3$(diethylenetriamine)]$^+$.

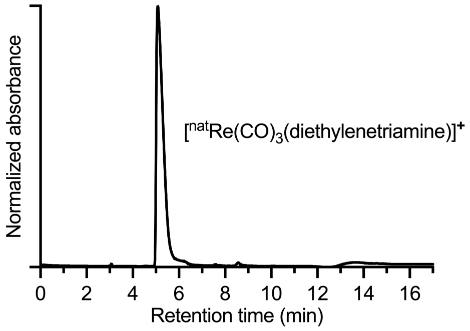

Figure S44. HRMS (ESI+) spectrum of [$^{nat}Re(CO)_3$(diethylenetriamine)]$^+$.

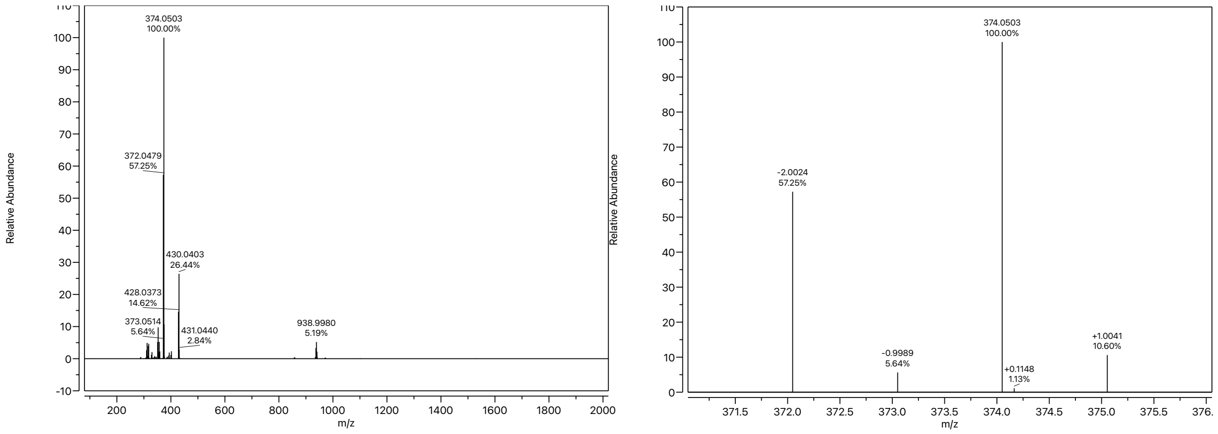

Electronic Absorption (UV-VIS) Spectroscopy:

Figure S45. Electronic absorption spectra of ligand L1 (green), the non-photoactive complex ^{nat}Re-diethylenetriamine (black) and the complex ^{nat}Re-L1$^+$ (blue) (0.1 mM, 30 °C, MeOH).

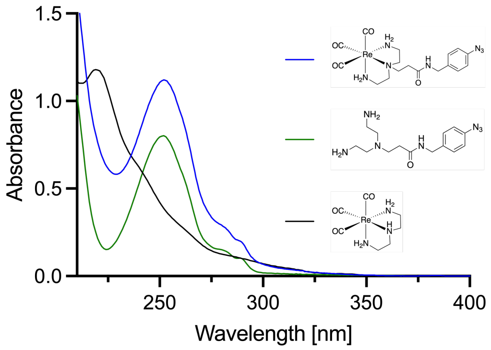

Figure S46. Electronic absorption spectra of a dilution series of ligand L1 (30 °C, MeOH).

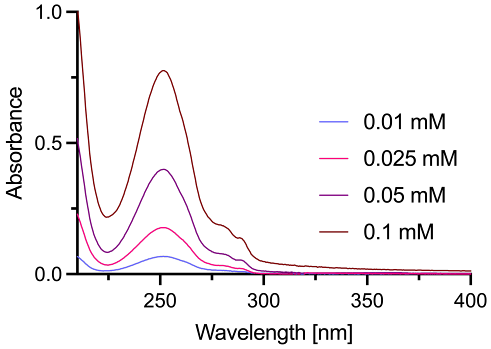

Figure S47. Electron absorption spectra of a dilution series of the complex ^{nat}Re-L1$^+$ (30 °C, MeOH).

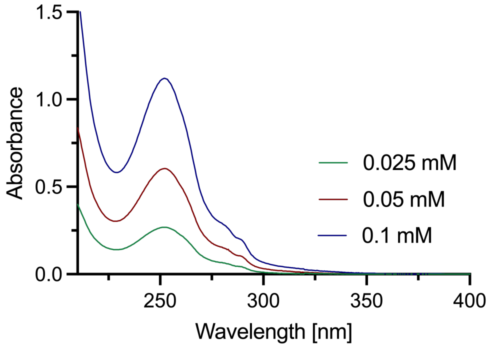

Figure S48. Molar absorption coefficients of L1 (30 °C, MeOH) at selected wavelengths.

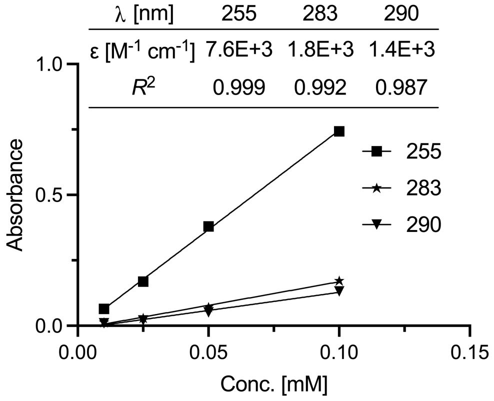

λ [nm]	255	283	290
ε [M^{-1} cm^{-1}]	7.6E+3	1.8E+3	1.4E+3
R^2	0.999	0.992	0.987

Figure S49. Molar absorption coefficients of ^{nat}Re-L1$^+$ (30 °C, MeOH).

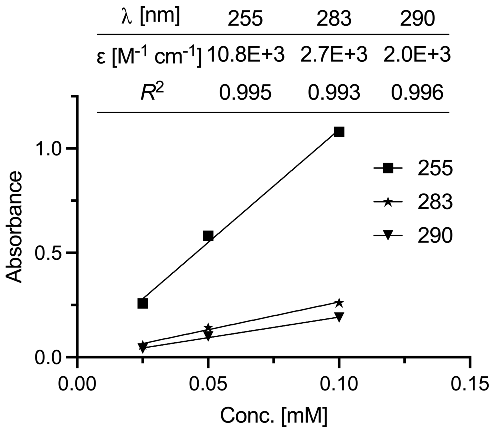

Figure S50. Plot of the relative change in concentration of complex ^{nat}Re-L1$^+$ and the ligand L1 *versus* time during photolysis at 395 nm, rate constant determined by first order rate law (0.1 mM, 25 °C, H_2O, n = 3).

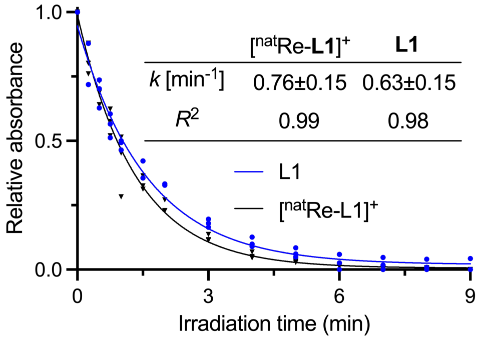

Radiosynthesis of [^{188}Re(CO)$_3$(diethylenetriamine)]$^+$

Scheme S15. Radiosynthesis of [^{188}Re(CO)$_3$(diethylenetriamine)]$^+$

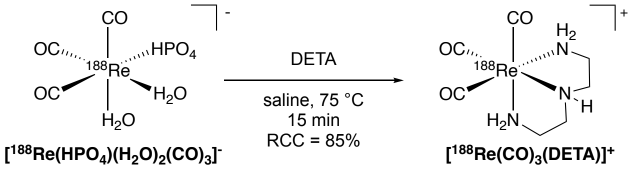

Radiolabeling reactions to prepare [^{188}Re(CO)$_3$(diethylenetriamine)]$^+$ were accomplished by the addition of 1 mL of freshly prepared [^{188}Re(HPO$_4$)(H$_2$O)$_2$(CO)$_3$]$^-$ stock solution to an aqueous solution of diethylenetriamine (DETA) (30 µL of 100 mg mL^{-1} stock in H_2O) under an N_2 atmosphere with a total reaction volume of 1.03 mL. Test reactions were performed with 10 to 50 MBq. The reactions were heated to 75 °C for 15 min. The reactions were monitored by analytical radio-HPLC and complexation was found to be complete in 15 min at 75 ºC giving a radiochemical conversion (RCC) >95% (R_t = 14.55 min). The complex [^{188}Re(CO)$_3$(diethylenetriamine)]$^+$ was obtained in a RCP >99%, a decay-corrected RCY >92±2% (n = 2), and molar activity of ~63.1 kBq nmol^{-1} (R_t = 14.85 min). The total reaction time was 15 min. The product was characterized by analytical HPLC following the

method described in the general section. Note: The UV-Vis detector and radioactivity detector were arranged serially with an offset time of approximately 0.40 min. The identity of the radiolabeled compound (^{188}Re-L1$^+$) was confirmed by co-injection with an authenticated sample of non-radioactive complex ^{nat}Re-L1$^+$.

Figure S51. Radio-HPLC chromatograms for [^{188}Re(HPO$_4$)(H$_2$O)$_2$(CO)$_3$]$^-$, [^{188}Re(CO)$_3$(diethylenetriamine)]$^+$ and [^{nat}Re(CO)$_3$(diethylenetriamine)]$^+$.

Note: The peak marked with * corresponds to [^{188}Re(H$_2$O)$_3$(CO)$_3$]$^+$.

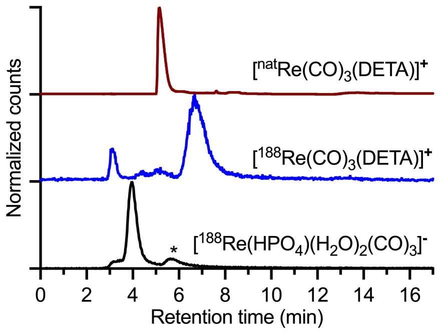

Figure S52. Analysis of the photolysis of [^{188}Re(CO)$_3$(diethylenetriamine)]$^+$ by HPLC.

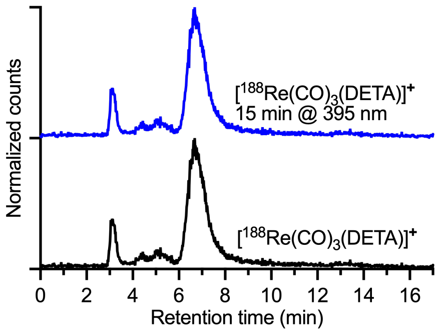

The bottom trace shows complex [^{188}Re(CO)$_3$(diethylenetriamine)]$^+$ and the top trace the complex after 15 min irradiation with 395 nm light for 15 min.

Figure S53. Analysis of the photolysis of [^{nat}Re(CO)$_3$(diethylenetriamine)]$^+$ by HPLC.

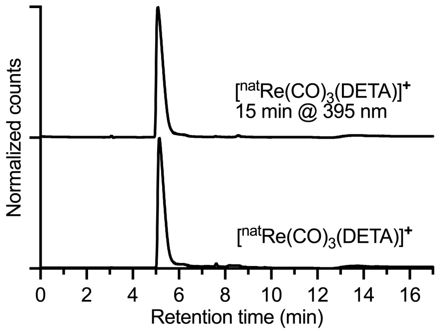

The bottom trace shows complex [^{nat}Re(CO)$_3$(diethylenetriamine)]$^+$ and the top trace the complex after 15 min irradiation with 395 nm light for 15 min.

Photoradiosynthesis of [^{188}Re(CO)$_3$L1-azepin]-HSA (^{188}Re-HSA)

Scheme S16. Radiosynthesis of ^{188}Re-HSA.

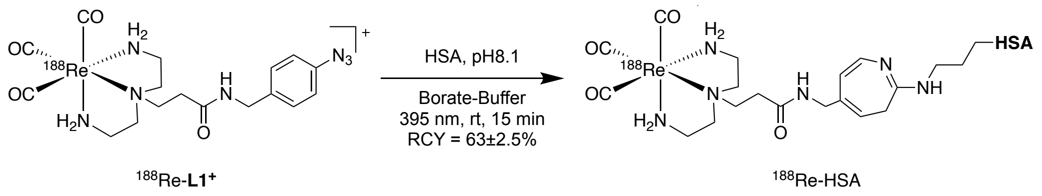

Photochemical conjugation reactions were performed in accordance with the following general procedure. ^{188}Re-L1$^+$ (9.961 MBq) in Borate buffer (0.025 M, pH=8.0-8.1, 250 mL) and HSA (60.0 mg mL^{-1}, MW = 66470 g mol^{-1}, PBS pH7.4, 100 µL, 6.0 mg, 90 nmol) was added to a 2 mL glass MS-vial at 23 °C (total volume reaction 350 µL, pH8.1). The initial chelate-to-mAb ratio was 81 to 1. The excess ligand is removed as described in the radiosynthesis of ^{188}Re-L1$^+$ and shown in Supplemental Figure S41. The reaction mixture was gently stirred and irradiated at 100% LED intensity for 15 min at 395 nm. Aliquots of the crude reaction mixtures were retained for analysis. The remaining material was purified by preparative PD-10 SEC (collecting the 1.4–3.0 mL high molecular weight fraction using sterile PBS as an eluent). Crude and purified aliquots were analyzed by using analytical radio-iTLC, PD-10 SEC, and SEC-HPLC. The isolated decay-corrected radiochemical yield (RCY) of ^{188}Re-HSA was 63±3 % (n = 3) and the lower limit of the molar activity of the product (estimated by assuming no protein losses) was ~0.073 MBq nmol^{-1} of protein, with an activity concentration of 4.109 MBq mL^{-1}. The radiochemical purity of the purified samples of ^{188}Re-HSA was estimated to be >99% (measured by PD-10 SEC and SEC-HPLC).

Figure S54. Radio-iTLC chromatogram of ^{188}Re-HSA.

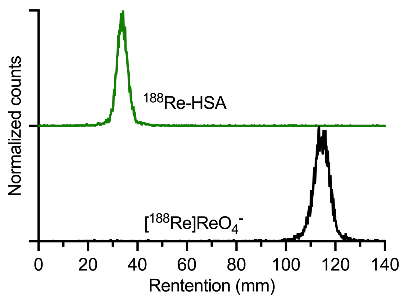

Figure S55. Preparative PD-10 SEC profile for the purification of ^{188}Re-HSA.

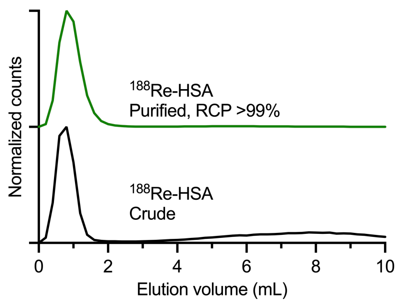

Figure S56. Preparative PD-10 SEC profile with the full elution of the crude ^{188}Re-HSA.

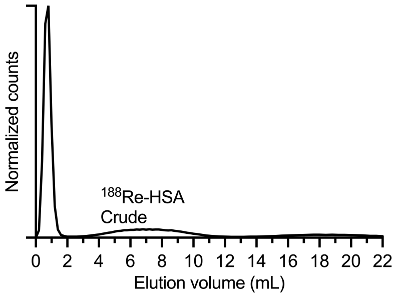

Figure S57. Size-exclusion (HPLC) chromatogram of ^{188}Re-HSA.

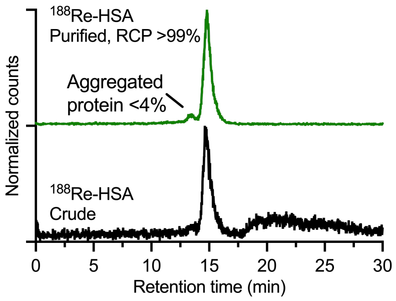

Figure S58. Preparative PD-10 SEC profile of the dark control reaction of the complex ^{188}Re-L1$^+$, ^{188}Re-L1$^+$ with HSA (dark control), and ^{188}Re-HSA after photolysis.

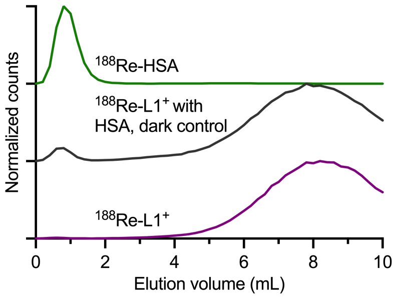

Figure S59. Preparative PD-10 SEC profile of ^{188}Re-L1$^+$ and the photolyzed complex ^{188}Re-L1-azepin-2-ol.

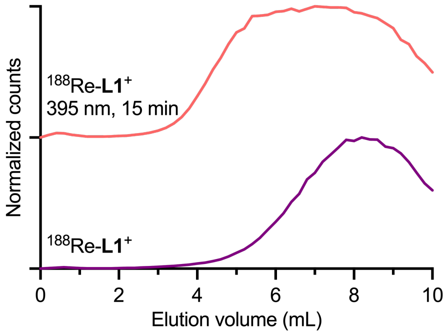

Figure S60. Preparative PD-10 SEC profile of [$^{99m}TcO_4]^-$ and [$^{188}ReO_4]^-$.

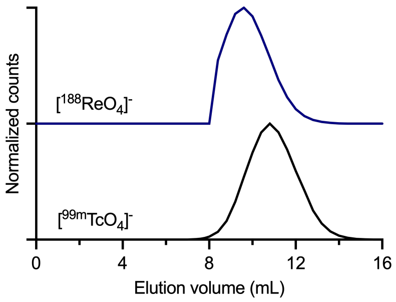

Figure S61. Stability test of ^{188}Re-HSA in (A) PBS, and (B) analysis of the small molecule fraction after 24 h by radio-HPLC.

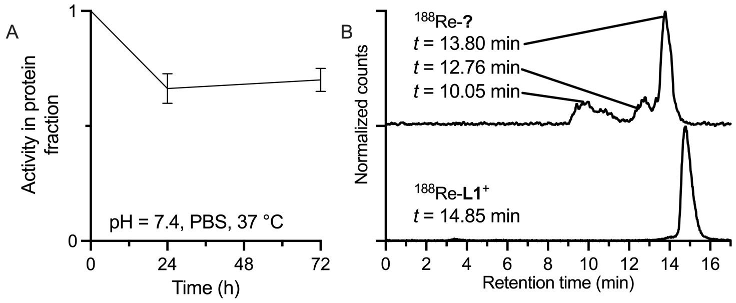

Photograph S1. Reaction setup of the photoradiolabeling reaction.

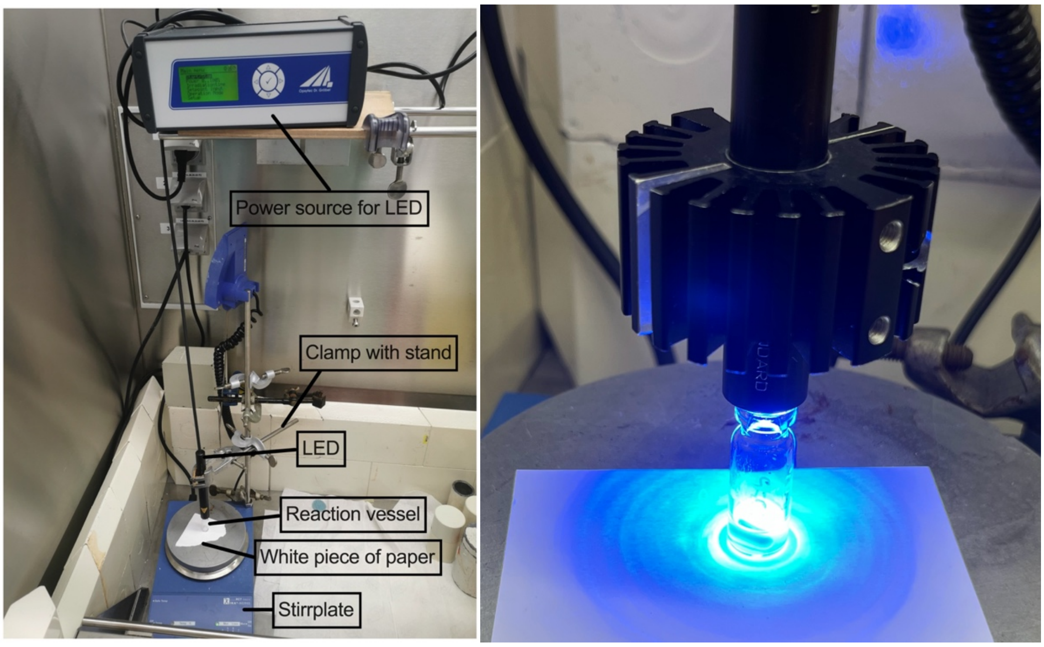

Photoradiosynthesis of [^{99m}Tc(CO)$_3$L1]-azepin-onartuzumab (^{99m}Tc-onartuzumab).

Scheme S15. Radiosynthesis of ^{99m}Tc-onartuzumab.

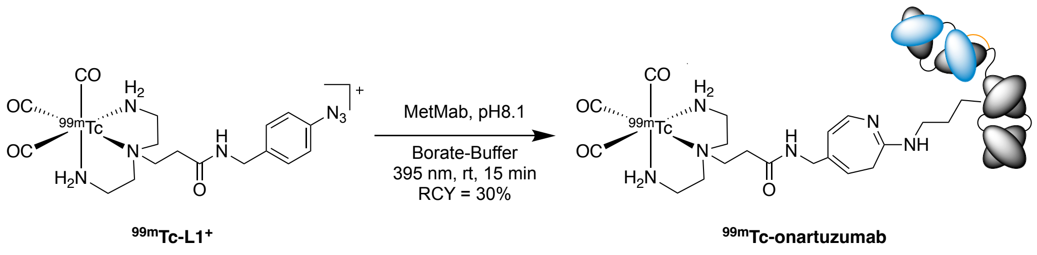

Photochemical conjugation reactions were performed in accordance with the following general procedure. ^{99m}Tc-L1^{+} (418.8 MBq, 243 nmol of L1) in sodium borate buffer (0.1 M, pH8.0-8.1, 150 mL) and onartuzumab (60.0 mg mL^{-1}, MW = 99160 g mol^{-1}, PBS pH7.4, 50 µL, 3 mg, 30 nmol) was added to a 2 mL glass MS-vial at 23 °C (total volume reaction 150 µL, pH8.1). The initial chelate-to-mAb ratio was 8 to 1. The final protein concentration was 15 mg mL^{-1}. The reaction mixture was gently stirred and irradiated at 100% LED intensity for 15 min at 395 nm. Aliquots of the crude reaction mixtures were retained for analysis. The remaining material was purified by preparative PD-10 SEC (collecting the 0–1.6 mL high molecular weight fraction using sterile PBS as an eluent). Crude and purified aliquots were analyzed by using analytical radio-iTLC, manual PD-10 SEC, and automated SEC-HPLC. The isolated decay-corrected radiochemical yield (RCY) of [^{99m}Tc(CO)$_3$L1]-azepin-onartuzumab was 30% (n = 1) and the lower limit of the molar activity of the product (estimated by assuming no protein losses) was ~4.146 MBq nmol^{-1} of protein, with an activity concentration of 77.74 MBq mL^{-1}. The radiochemical purity of the purified samples of [^{99m}Tc(CO)$_3$L1]-azepin-onartuzumab was estimated to be >99% (measured by PD-10 SEC and SEC-HPLC).

Figure S62. iTLC chromatogram of ^{99m}Tc-onartuzumab.

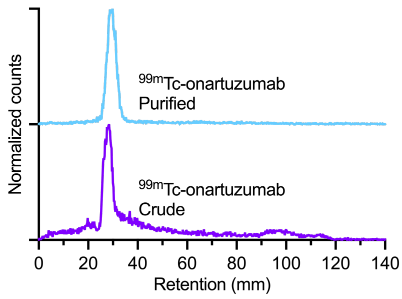

Figure S63. Preparative PD-10 SEC profile for the purification of ^{99m}Tc-onartuzumab.

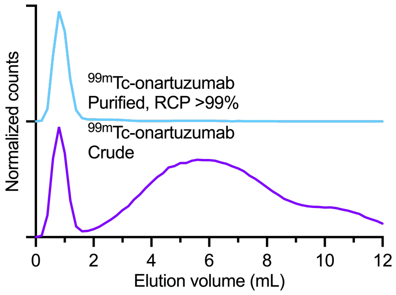

Figure S64. Size-exclusion chromatogram of ^{99m}Tc-onartuzumab.

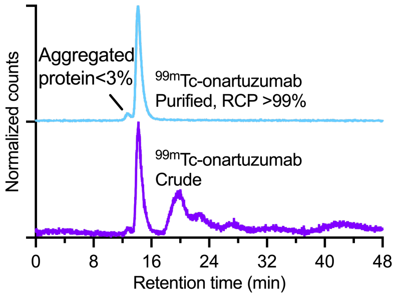

Photoradiosynthesis of [^{188}Re(CO)$_3$L1]-azepin-onartuzumab (^{188}Re-onartuzumab)

Scheme S16. Radiosynthesis of ^{188}Re-onartuzumab.

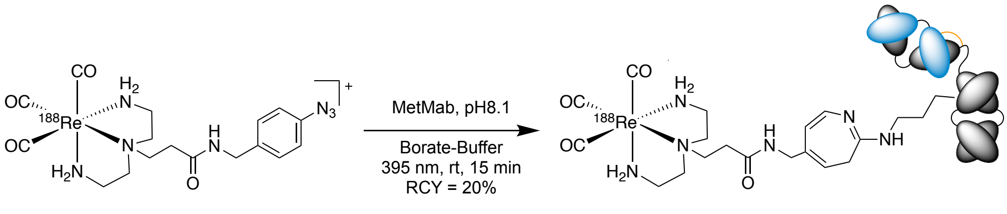

Photochemical conjugation reactions were performed in accordance with the following general procedure. ^{188}Re-L1$^+$ (91.0 MBq, 7.29 µmol of L1) in Borate buffer (0.1 M, pH=8.0-8.1, 100 mL) and onartuzumab (60.0 mg mL^{-1}, MW = 99160 g mol^{-1}, PBS pH7.4, 30 µL, 1.8 mg, 18 nmol) was added to a 2 mL glass MS-vial at 23 °C (total volume reaction 130 µL, pH8.1). The initial chelate-to-mAb ratio was 405 to 1. The final protein concentration was 14 mg/ml. The reaction mixture was gently stirred and irradiated at 100% LED intensity for 15 min at 395 nm. Aliquots of the crude reaction mixtures were retained for analysis. The remaining material was purified by preparative PD-10 SEC (collecting the 0–1.6 mL high molecular weight fraction using sterile PBS as an eluent). Crude and purified aliquots were analyzed by using analytical radio-iTLC, PD-10 SEC, and SEC-HPLC. The isolated decay corrected radiochemical yield (RCY) of ^{188}Re-onartuzumab was 20% (n = 1) and the lower limit of the molar activity of the product (estimated by assuming no protein losses) was ~1.026 MBq nmol^{-1} of protein, with an activity concentration of 11.54 MBq mL^{-1}. The radiochemical purity of the purified samples of ^{188}Re-onartuzumab was estimated to be >95% (measured by PD-10 SEC and SEC-HPLC).

Figure S65. iTLC chromatogram of ^{188}Re-onartuzumab.

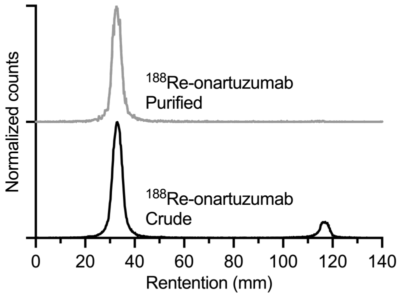

Figure S66. Preparative PD-10 SEC profile for the purification of ^{188}Re-onartuzumab.

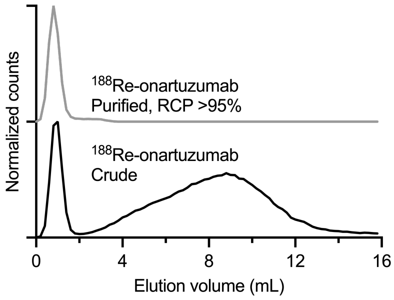

Figure S67. Size-exclusion chromatogram of ^{188}Re-onartuzumab.

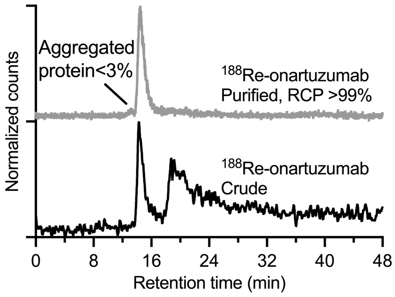

Radiochemical stability and cellular binding of ^{99m}Tc/^{188}Re-onartuzumab

Figure S68. Stability test of ^{99m}Tc-onartuzumab in (A) PBS, and (B) analysis of the small molecule fraction after 24 h by radio-HPLC.

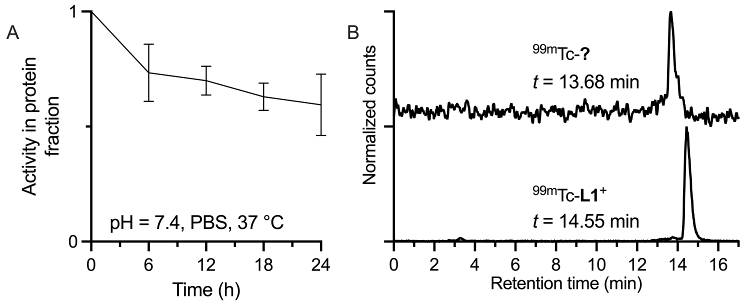

Figure S69. Stability test of ^{188}Re-onartuzumab in PBS.

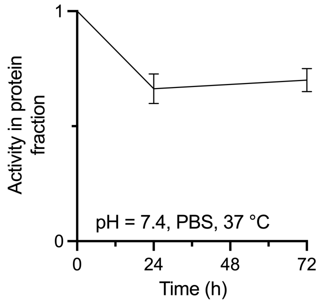

Figure S70. Cellular binding assay of ^{99m}Tc-onartuzumab on 15 × 10^6 MKN-45 cells.

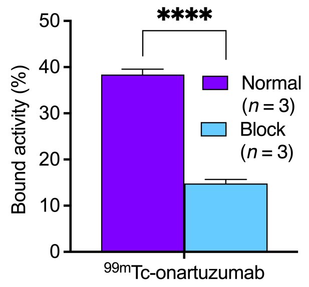

Figure S71. Cellular binding assay of ^{188}Re-onartuzumab on 15 × 10^6 MKN-45 cells.

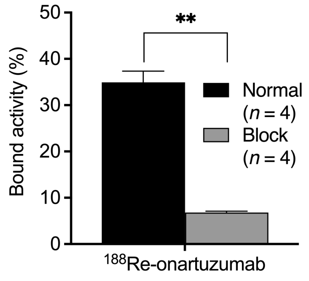

Animal studies and preparation of M-onartuzumab (M = ^{99m}Tc or ^{188}Re) doses for injection in mice

An aliquot of the purified and formulated sample of [M(CO)$_3$L1]-azepin-onartuzumab (M-onartuzumab) was added to a sterile vial and diluted with sterile PBS to a final volume of 900 mL. The tail of each mouse was warmed gently by using a warm water bath (~30 ºC) immediately before administering M-onartuzumab. Two groups of animals were used and each mouse in the normal group (*n* = 4 mice) received a dose (50 µg of protein, in 150 µL sterile PBS) *via* intravenous (i.v.) tail-vein injection (*t* = 0 h). For the competitive inhibition (blocking) experiments, a separate aliquot of M-onartuzumab was added to a sterile vial. Then an aliquot of the stock solution of MetMAb (fully formulated; 60.0 mg mL^{-1}, 100 µL, 6.0 mg of protein) was added to reduce the molar activity (total ~1.05 mg of protein/mouse) and the mixture was diluted with sterile PBS to a final volume of 900 mL. Animals in the blocking group (*n* = 4 mice) each received an i.v. dose (in 150 µL sterile PBS).

Table S1. Doses for the animal experiments.

	[^{99m}Tc(CO)$_3$L1]-azepin-onartuzumab 24 h, *n* = 4		[^{188}Re(CO)$_3$L1]-azepin-onartuzumab 24 h, *n* = 4		[^{188}Re(CO)$_3$L1]-azepin-onartuzumab 72 h, *n* = 4	
	normal	block	normal	block	Normal	block
Volume of stock M-onartuzumab [µL]	194	194	265	265	236	236
Activity total [MBq]	10.750	10.430	2.887	2.880	3.468	3.763
Activity per mouse [MBq]	1.750-1.811	1.686-1.762	0.556-0.562	0.571-0.629	0.621-0.712	0.601-0.699

Planar γ-scintigraph images

Figure S72. Maximum intensity projection (MIP) ^{188}Re-onartuzumab planar g-scintigraph images of a representative mouse. T = tumor.

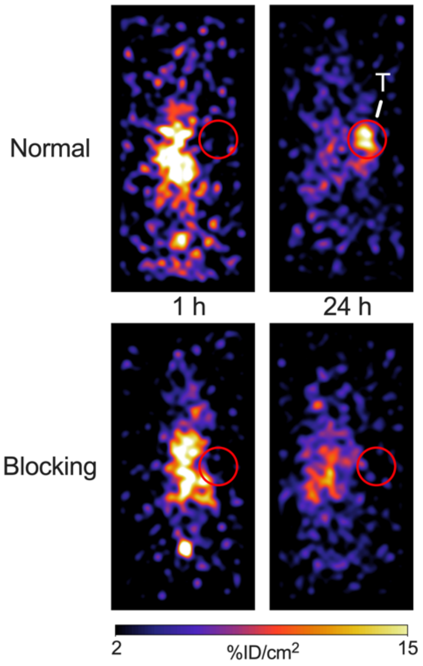

Figure S73. Maximum intensity projection (MIP) ^{99m}Tc-onartuzumab planar γ-scintigraph images of a representative mouse. T = tumor.

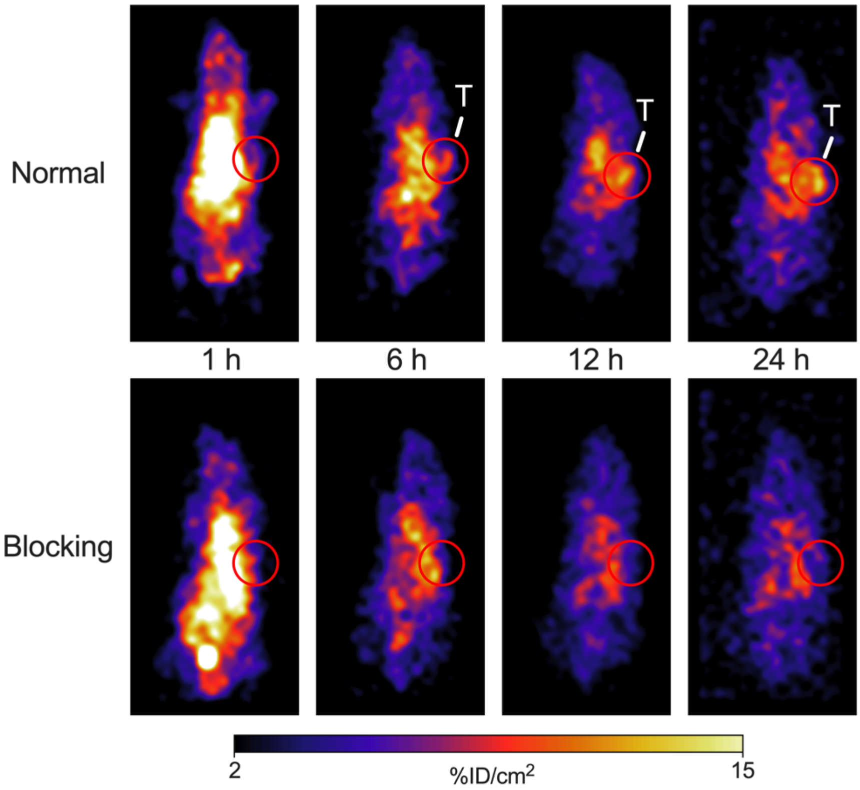

Biodistribution results

Figure S74. Comparison of the biodistribution results expressed as tissue uptake (%ID/g).

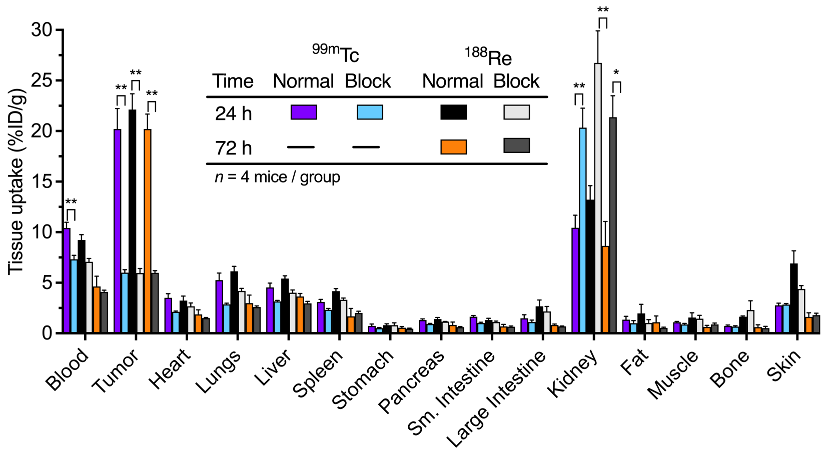

Figure S75. Comparison of the biodistribution results expressed as tissue uptake (SUV).

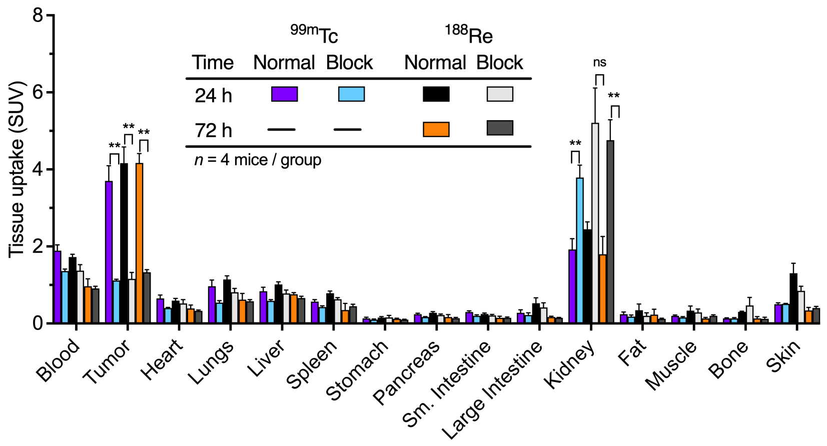

Figure S76. Comparison of the tumor-to-tissue contrast ratio based on values reported in %ID g^{-1}.

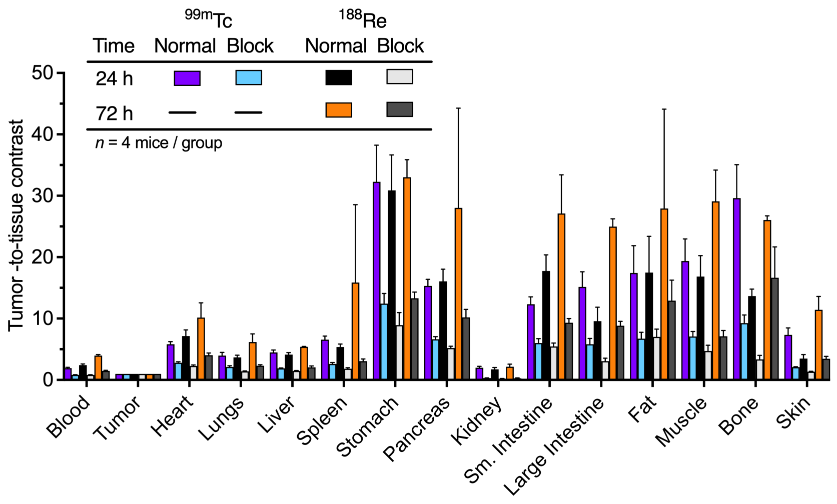

Table S2. *Ex vivo* biodistribution data measured for ^{99m}Tc- and ^{188}Re-onartuzumab in the normal groups.

Tissue	[^{99m}Tc(CO)$_3$L1]-azepin-onartuzumab 24 h, (Normal group, *n* = 4)		[^{188}Re(CO)$_3$L1]-azepin-onartuzumab 24 h, (Normal group, *n* = 4)		[^{188}Re(CO)$_3$L1]-azepin-onartuzumab 72 h, (Normal group, *n* = 4)	
	Uptake / %ID g^{-1} ± S.D.[a]	Tumor-to-tissue contrast ratio ± S.D.[b]	Uptake / %ID g^{-1} ± S.D.[a]	Tumor-to-tissue contrast ratio ± S.D.[b]	Uptake / %ID g^{-1} ± S.D.[a]	Tumor-to-tissue contrast ratio ± S.D.[b]
Blood	10.41 ± 1.15	1.93 ± 0.25	9.23 ± 1.04	2.41 ± 0.39	4.64 ± 1.02	3.96 ± 0.17
Tumor	20.20 ± 4.06	1.00 ± 0.00	22.13 ± 3.11	1.00 ± 0.00	20.21 ± 1.47	1.00 ± 0.00
Heart	3.51 ± 0.81	5.82 ± 0.83	3.24 ± 0.90	7.16 ± 1.98	1.88 ± 0.45	10.18 ± 2.40
Lungs	5.26 ± 1.41	3.97 ± 1.07	6.14 ± 0.96	3.67 ± 0.73	2.98 ± 0.81	6.17 ± 1.33
Liver	4.55 ± 0.86	4.48 ± 0.83	5.41 ± 0.55	4.12 ± 0.65	3.65 ± 0.28	5.39 ± 0.07
Spleen	3.10 ± 0.52	6.56 ± 1.15	4.18 ± 0.49	5.36 ± 1.01	1.68 ± 0.79	15.86 ± 12.69
Stomach	0.73 ± 0.40	32.3 ± 11.97	0.80 ± 0.30	30.9 ± 11.56	0.55 ± 0.12	33.02 ± 2.86
Pancreas	1.32 ± 0.21	15.3 ± 2.18	1.42 ± 0.31	16.1 ± 3.94	0.82 ± 0.29	28.03 ± 16.24
Kidney	10.43 ± 2.50	1.98 ± 0.46	13.22 ± 2.75	1.74 ± 0.48	8.65 ± 2.41	2.17 ± 0.40
Sm. Int.	1.65 ± 0.23	12.3 ± 2.39	1.32 ± 0.32	9.59 ± 5.28	0.70 ± 0.19	27.11 ± 6.31
Large Int.	1.48 ± 0.70	15.2 ± 4.94	2.67 ± 1.26	9.69 ± 4.55	0.83 ± 0.09	24.98 ± 1.26
Fat	1.35 ± 0.66	17.4 ± 8.86	1.98 ± 1.79	9.23 ± 11.82	1.09 ± 0.64	27.95 ± 16.16
Muscle	1.09 ± 0.19	19.4 ± 7.18	1.56 ± 0.94	13.10 ± 6.79	0.64 ± 0.15	29.09 ± 5.11
Bone	0.73 ± 0.20	29.6 ± 10.90	1.64 ± 0.15	3.01 ± 2.27	0.61 ± 0.23	26.04 ± 0.68
Skin	2.78 ± 0.41	7.33 ± 2.30	6.91 ± 2.49	2.57 ± 1.25	1.62 ± 0.41	11.42 ± 2.19

[a] Uptake data are expressed as the mean %ID g^{-1} ± one standard deviation (S.D. / %ID g^{-1}).

[b] Errors for the tumor-to-tissue ratios are calculated as the standard deviations based on ratios from dependent pairs.

Table S3. *Ex vivo* biodistribution data measured for ^{99m}Tc- and ^{188}Re-onartuzumab in the blocking groups.

Tissue	[^{99m}Tc(CO)$_3$L1]-azepin-onartuzumab 24 h, (Blocking group, *n* = 4)		[^{188}ReCO)$_3$L1]-azepin-onartuzumab 24 h, (Blocking group, *n* = 4)		[^{188}Re(CO)$_3$L1]-azepin-onartuzumab 72 h, (Blocking group, *n* = 4)	
	Uptake / %ID g^{-1} ± S.D.[a]	Tumor-to-tissue contrast ratio ± S.D.[b]	Uptake / %ID g^{-1} ± S.D.[a]	Tumor-to-tissue contrast ratio ± S.D.[b]	Uptake / %ID g^{-1} ± S.D.[a]	Tumor-to-tissue contrast ratio ± S.D.[b]
Blood	7.33 ± 0.76	0.82 ± 0.07	7.08 ± 0.65	0.48 ± 0.05	4.09 ± 0.37	1.48 ± 0.21
Tumor	6.01 ± 0.57	1.00 ± 0.00	5.97 ± 0.87	1.00 ± 0.00	5.98 ± 0.44	1.00 ± 0.00
Heart	2.14 ± 0.13	2.82 ± 0.28	2.67 ± 0.68	2.28 ± 0.29	1.50 ± 0.14	4.04 ± 0.68
Lungs	2.87 ± 0.23	2.12 ± 0.37	4.20 ± 0.51	1.14 ± 0.09	2.60 ± 0.24	2.32 ± 0.32
Liver	3.17 ± 0.16	1.90 ± 0.15	4.02 ± 0.55	1.48 ± 0.13	2.97 ± 0.40	2.06 ± 0.46
Spleen	2.32 ± 0.29	2.62 ± 0.44	3.31 ± 0.37	1.82 ± 0.33	2.02 ± 0.35	3.05 ± 0.74
Stomach	0.51 ± 0.13	12.44 ± 3.30	0.81 ± 0.47	8.95 ± 4.06	0.46 ± 0.11	13.32 ± 2.00
Pancreas	0.92 ± 0.14	6.62 ± 0.88	1.15 ± 0.07	5.19 ± 0.60	0.61 ± 0.14	10.19± 2.58
Kidney	20.34 ± 3.86	0.30 ± 0.05	26.75 ± 6.32	0.22 ± 0.02	21.83 ± 4.23	0.29 ± 0.09
Sm. Int.	1.03 ± 0.15	6.00 ± 1.46	111 ± 0.21	5.47 ± 1.06	0.66± 0.14	9.31 ± 1.40
Large Int.	1.12 ± 0.37	5.81 ± 1.93	2.16 ± 1.01	3.07 ± 0.99	0.69 ± 0.08	8.83 ± 1.42
Fat	1.00 ± 0.50	6.73 ± 2.04	1.02 ± 0.68	7.03 ± 2.48	0.53 ± 0.16	12.91 ± 6.71
Muscle	0.88 ± 0.19	7.08 ± 1.61	1.45 ± 0.67	4.73 ± 1.82	0.89 ± 0.27	7.13 ± 1.87
Bone	0.68 ± 0.14	9.27 ± 2.64	2.31 ± 1.82	3.35 ± 1.32	0.52 ± 0.36	16.65 ± 10.04
Skin	2.85 ± 0.18	2.10 ± 0.09	4.39 ± 0.67	1.63 ± 0.08	1.80 ± 0.37	3.45 ± 0.78

[a] Uptake data are expressed as the mean %ID g^{-1} ± one standard deviation (S.D. / %ID g^{-1}).

[b] Errors for the tumor-to-tissue ratios are calculated as the standard deviations based on ratios from dependent pairs.

Effective and biological half-life measurements

The effective $t_{1/2}$(eff) and biological half-life $t_{1/2}$(biol) of ^{99m}Tc- and ^{188}Re-onartuzumab were measured in female athymic nude mice. Total internal radioactivity was measured as a function of time by using a dose calibrator.

Figure S77. One-phase model (Prism 10, 'One-phase' model) for the experimentally measured effective half-life of (A) ^{188}Re-onartuzumab (0 h to 22 h, n = 4), and (B) ^{188}Re-onartuzumab (17 h to 72 h, n = 4).

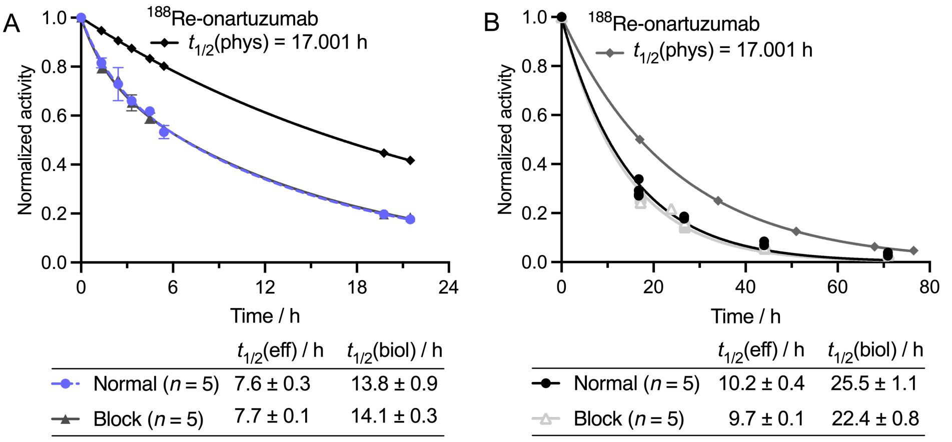

Figure S78. One-phase model for the experimentally measured effective half-life of ^{99m}Tc-onartuzumab (n = 4)

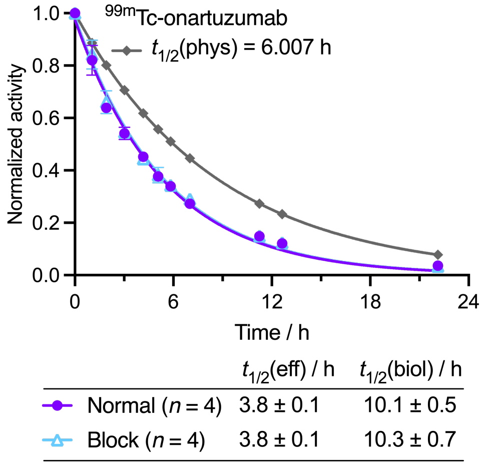

References

1 H. Xiang, B. C. Bender, A. E. Reyes, M. Merchant, N. L. Shasha Jumbe, M. Romero, T. Davancaze, I. Nijem, E. Mai, J. Young, A. Peterson and L. A. Damico-Beyer, *Clinical Cancer Research*, 2013, 19, 5068–5078.

2 M. Georgiou, E. Fysikopoulos, K. Mikropoulos, E. Fragogeorgi and G. Loudos, *Mol Imaging Biol*, 2017, 19, 398–407.

3 M. H. Jeong, Y. Choi, Y. H. Chung, T. Y. Song, J. H. Jung, K. J. Hong, B. J. Min, Y. S. Choe, K. H. Lee and B. T. Kim, *Phys Med Biol*, 2004, 49, 4961.

4 A. R. Pradipta, M. Taichi, I. Nakase, E. Saigitbatalova, A. Kurbangalieva, S. Kitazume, N. Taniguchi and K. Tanaka, *ACS Sens*, 2016, 1, 623–632.
